# Dual Role of Necroptosis in Cervical Cancer: Promoting Tumor Aggression and Modulating the Immune Microenvironment via the JAK2-STAT3 Pathway

**DOI:** 10.7150/jca.98738

**Published:** 2024-08-13

**Authors:** Fangfang Xu, Yingjun Ye, Yueqing Gao, Shaohua Xu

**Affiliations:** Shanghai Key Laboratory of Maternal Fetal Medicine, Shanghai Institute of Maternal-Fetal Medicine and Gynecologic Oncology, Shanghai First Maternity and Infant Hospital, School of Medicine, Tongji University, Shanghai 200092, China.

**Keywords:** Necroptosis, Cervical cancer, JAK2-STAT3 signaling pathway, Bevacizumab, Tumor immune microenvironment, T cells

## Abstract

In the dynamic landscape of cervical cancer (CC) pathophysiology, this study aimed to elucidate the role of necroptosis in modulating tumor proliferation, invasion, and the immune microenvironment in CC. In this study, the impact of necroptosis on CC was evaluated through a series of bioinformatical analyses and experimental approaches. The impact of necroptosis on CC was illustrated by analyzing its effects on tumor aggression, immune responses, and the JAK2-STAT3 signaling pathway. Bevacizumab, a monoclonal antibody targeting vascular endothelial growth factor (VEGF), was also evaluated for its potential induction of necroptosis in CC cells and its interaction with necroptosis inhibitors. Additionally, the study assessed the influence of necroptosis on the immune microenvironment, particularly in T-cell-related pathways and the expression of tumor suppressor genes in CC. Necroptosis was found to enhance VEGFA expression through the activation of the JAK2-STAT3 pathway, promoting tumor proliferative and invasive capabilities in CC. Bevacizumab induced necroptosis in CC cells, potentially leading to resistance to therapy. The combination of bevacizumab with necroptosis inhibitors attenuated VEGFA expression, suggesting a novel therapeutic strategy. Additionally, necroptosis activated T-cell-related pathways and promoted the infiltration and activation of Jurkat T cells. CD3D—a tumor suppressor gene in CC—was identified as a critical marker and its expression could be upregulated by necroptosis via the JAK2-STAT3 pathway in Jurkat T cells. Treatment of CC cells with supernatants from necroptosis-induced Jurkat cells resulted in reduced tumor cell proliferation and invasion. This study reveals a complex interaction between necroptosis, tumor progression, and the immune response in CC. The findings propose a nuanced approach to leveraging necroptosis for therapeutic interventions, highlighting the potential of combining necroptosis inhibitors with existing therapies to improve treatment outcomes in CC.

## Introduction

Cervical cancer (CC) ranks among the most frequently diagnosed malignancies in women. An estimated 604,127 females worldwide received a new diagnosis of CC in 2020, with up to 341,831 of them passing away as a result of the condition 1, posing a tremendous burden on healthcare systems. Radical hysterectomy is commonly used for early-stage CC, which has achieved great success 2. In general, patients diagnosed with early-stage CC have a favorable prognosis 3, while outcomes for locally advanced stages, recurrent and metastatic cases are not as promising 4. For patients with locally advanced stages, metastatic and recurrent CC, conventional treatment options include surgery, radiotherapy and chemotherapy, but these treatment options exert limited effects, the 5-year survival rate for metastatic or recurrent patients is no more than 20% 5,6. In recent years, immunotherapy especially immune checkpoint inhibitors (ICIs) has emerged to be a promising strategy to treat different human malignancies. Anti-programmed cell death (PD-1) and anti-cytotoxic T lymphocyte-associated antigen 4 (CTLA-4) are two targets for immunotherapy under research in a range of malignancies including CC 7-9. Dishearteningly, the therapeutic effects of the common ICIs including PD-L1 and PD-1 in CC are limited, with a poor response rate of 10-25% 10. Furthermore, even though the angiogenesis inhibitor bevacizumab has been employed in combination with the anti-programmed cell death ligand-1 (PD-L1) antibody atezolizumab to enhance the efficacy of CC therapy, the combination therapy only results in a modest improvement 11. So, it is of great significance to explore more potential immunotherapy targets and cell interactions within the tumor microenvironment to improve the immunotherapy effectiveness for CC patients.

Necroptosis, a form of regulated cell death, is mainly mediated by receptor interacting protein kinase 1 (RIPK1), RIPK3, and mixed lineage kinase domain-like pseudokinase (MLKL) 12,13. For the past few years, an accumulating body of research has indicated that necroptosis plays a dual role in modulating tumorigenesis and tumor progression 14. For one thing, necroptosis is suggested to act as a contributing factor in cancer metastasis and cancer progression 15,16, for another, necroptosis is also reported to serve as a tumor suppressor in many malignancies 17,18, either promoting or defending against tumor depends on cancer subtypes and the context in which tumor cells are located. Therefore, a comprehensive investigation into necroptosis may prove beneficial in developing a framework for personalized treatment plans for patients. What's more, necroptosis plays a role in the regulation of tumor immune microenvironment and its significance in cancer immunity has been increasingly appreciated. It was demonstrated that cancer cells undergoing necroptosis can activate cytotoxic CD8+ T cells and trigger anti-tumor immunity 19,20. In addition to the regulation of T cells, necroptosis also affects other immune cells, as a key regulator of necroptosis, it was reported that the decreased level of RIPK3 was relevant to the accumulation of myeloid-derived suppressor cells (MDSCs) in the tumor microenvironment (TME) 21. Additionally, necroptosis is suggested to play a negative regulatory role in tumor immunity. It was proved that pancreatic epithelial cells undergoing necroptosis induce C-X-C Motif Chemokine Ligand 1 (CXCL1), leading to the suppression of adaptive immunity and pancreatic oncogenesis 22. To sum up, necroptosis exerts a complex regulatory influence on cancer biology and has potential applications as a source of novel drug targets. However, there are few studies on necroptosis in the immune microenvironment of CC. So, it is urgently required to investigate the impact of necroptosis in CC, which may provide valuable insights into patient stratification and precision immune treatment.

This study seeks to elucidate the role of necroptosis in CC pathogenesis and its influence on the tumor immune microenvironment. Through integrated bioinformatics analysis of the TCGA-CESC cohort and functional in vitro assays, we aimed to characterize the dual role of necroptosis in CC, which on the one hand promotes the proliferation of CC cells and on the other hand can exert anti-tumor effects through eliciting activation of T cells. Its pro-cancer and anti-cancer effects are achieved through the JAK2-STAT3 signaling pathway. Firstly, it exerts an anti-tumor effect by activating the JAK2-STAT3 pathway, which upregulates CD3D and then promotes T-cell activation. Secondly, it exerts a pro-cancer effect by activating the JAK2-STAT3 pathway, which upregulates VEGFA. Ultimately, CD3D was determined as a biomarker for immune activation and therapeutic responsiveness in CC patients, and the role of CD3D as a biomarker of treatment response was described by exploring the relationship between CD3D expression and clinical characteristics and immune microenvironment of CC. The study extends to the potential interaction between necroptosis and bevacizumab, revealing a potential resistance mechanism of bevacizumab and offering a new perspective on personalized treatment plans for CC patients.

## Materials and methods

### Public datasets acquisition

The gene expression profiles and clinical data for CC cases were downloaded from The Cancer Genome Atlas (TCGA) database, accessible through https://portal.gdc.cancer.gov/. Necroptosis-related genes (NRGs) were retrieved from MSigDB (https://www.gsea-msigdb.org/) via the search term “Necroptosis”. In addition, genes related to the CD8 pathway were downloaded from MSigDB by searching the keyword “CD8”. CC samples were grouped by the expression level of NRGs and defined as “high-necroptosis” and “low-necroptosis” cohorts. Subsequently, the identification of differentially expressed genes (DEGs) between two cohorts was performed using the “limma” package in R, applying the following filtering criteria: false discovery rate (FDR) < 0.05 and absolute log2 fold change (FC) > 1. Then, the intersection of necroptosis-related differentially expressed genes, CD8 pathway-related genes, and prognostic-related genes was identified through the R package “VennDiagram”.

### Immunity evaluations

The ESTIMATE algorithm implemented in R software 23 was utilized to evaluate the Immune Score, Stromal Score, and overall ESTIMATE Score for each CC sample, indicating the extent of immune cell and stromal cell infiltration within the TME. Single-sample Gene Set Enrichment Analysis (ssGSEA) was applied to quantify the presence of immune cells and the enrichment of immune-related pathways via the “GSVA” R package 24. The CIBERSORT algorithm was utilized to determine the composition of 22 immune cell types within the TME of each CC sample 25. Immune checkpoint genes (ICPs) were sourced from the TISIDB database (http://cis.hku.hk/TISIDB) 26 and prior research papers 27-29. The difference in immune components between different groups was visualized using R packages “limma”, “ggplot” and “ggplot2”.

### Functional enrichment analyses and GSEA

Gene Ontology (GO) and Kyoto Encyclopedia of Genes and Genomes (KEGG) analyses were conducted to investigate the biological function of NRGs through the R package “clusterProfiler” 30, with the outcomes visualized using the R packages “enrichplot” and “ggplot2”. A significant threshold of p-value <0.05 and q-value <0.05 was utilized. Additionally, gene set enrichment analysis (GSEA) was conducted between high- and low-necroptosis clusters using the HALLMARK gene sets and C2, CP, and KEGG v7.2 symbols. This analysis was performed via the gsea software (version: 4.0.3) with a significant threshold of 5% for NOM p-value and FDR q-value.

### Survival analysis

R packages “survival” and “survminer” 31 were employed to delimit the cut-off value of all survival analyses. The survival curves were generated using the Kaplan-Meier (KM) method to display all results, with statistical significance calculated using the log-rank test. A significance level of p<0.05 was considered to be of notable importance. The impact of gene expression on survival was also assessed using the KM Plotter online database (http:// kmplot.com).

### Necroptosis induction and inhibition

Necroptosis was induced by treating cells with a combination of TNFα (MCE, HY-P7058) and Z-VAD-FMK (MCE, HY-16658B) at concentrations of 20 μm and 50 μm. This treatment regimen aimed to inhibit caspase activity and initiate necroptotic cell death. To inhibit necroptosis, cells were exposed to necrosulfonamide (NSA) (MCE, HY-100573) or necrostatin-1 (NEC-1) (MCE, HY-15760) at concentrations of 10 μm, 20 μm, and 30 μm. NSA targets the effector protein MLKL to prevent its translocation to the plasma membrane, while NEC-1 disrupts the formation of the RIPK1-RIPK3 complex crucial for necroptotic signaling. These interventions were instrumental in elucidating the molecular mechanisms underlying necroptosis regulation in our experimental model.

### Cell culture and real-time qPCR (RT-qPCR)

The SiHa cell lines, sourced from ATCC (Manassas, VA, USA), were cultured in high-glucose Dulbecco's modified Eagle medium (DMEM; Servicebio, China) supplemented with 10% fetal bovine serum (FBS, Biological Industries, Israel) and 1% antibiotic (penicillin/streptomycin; New Cell & Molecular Biotech Co., China) at 37°C in a 5% CO2 incubator. Human Jurkat Clone E6-1 T cells were procured from Procell Life Science & Technology Co., Ltd (#CL-0129; Wuhan, China) and maintained in RPMI-1640 medium (Servicebio, China) with 10% FBS and 1% penicillin/streptomycin.

Total RNA was isolated from SiHa and human Jurkat Clone E6-1 T cells using TRIzol reagent (Invitrogen, USA), followed by quantification of RNA concentration and purity. Reverse transcription to generate cDNA was performed using the 5× ALL-IN-One RT Master Mix kit (Applied Biological Materials Inc., Canada). RT-qPCR was carried out using the TB Green Premix Ex Taq kit (Takara, Japan), with GAPDH expression used as an internal control. The relative mRNA expression levels were determined using the 2^-△Ct^ method 32. Table S1 lists all the primers used in this research.

### Clinical CC samples and immunohistochemistry (IHC)

Ten cervical squamous cell carcinoma specimens, consisting of five early-stage and five late-stage samples, were obtained from Shanghai First Maternity and Infant Hospital. These clinical specimens underwent pathological verification according to the latest International Federation of Gynecology and Obstetrics (FIGO) guidelines. Prior to collection, informed consent was obtained from the patients, and the study was approved by the Medical Ethics Committee of Shanghai First Maternity and Infant Hospital (Approval notice: KS21264).

Following fixation, paraffin embedding, dewaxing, rehydration, and antigen retrieval, all tumor tissue sections were processed for staining with a primary antibody targeting CD3D (A9770, Abclonal, China) at 4°C overnight. Subsequently, the slides underwent incubation with a secondary antibody (#PK-8501, Vector Lab, USA) for 1 hour at 37°C, followed by detection and visualization of the DAB complex using the Rabbit IgG mini-PLUS Kit (#PK-8501, Vector Lab, USA) and hematoxylin for nuclear counterstaining. Photomicrographs were captured using an optical microscope at 100× magnification.

### Western blotting (WB)

Cell lysis was performed by adding RIPA buffer containing complete protease and phosphatase inhibitors (TargetMol, America). Protein concentrations were determined using the BCA method (Beyotime, China). The extracted proteins were subjected to a thermal treatment at 99℃ for a period of 20 minutes. Subsequently, they were separated by 10% SDS-PAGE (Servicebio, China) and transferred to polyvinylidene fluoride membranes. Next, the membranes were blocked with a 5% lipid-free milk solution for 1.5 hours. Following this, they were incubated with primary antibodies (Table S2) overnight at 4°C. Afterward, the membranes were washed and incubated with secondary antibodies (HuaBio, China) for 2 hours at room temperature. Ultimately, protein bands were visualized using an enhanced chemiluminescence reagent (Epizyme, China).

### Cell Counting Kit-8 (CCK-8) assay

CCK-8 reagent (Vazyme, China) was utilized to detect the cell viability. CC cells and human Jurkat Clone E6-1 T cells were initially diluted to a density of 2*10^4^ cells/ml and plated in 100 µl aliquots into each well of 96-well microtiter plates (Coring, NY, USA) for 96 hours. At 24-hour intervals, 10 µl of CCK-8 reagent was added to each well, followed by a 2-hour incubation period. Subsequently, optical density (OD) was measured at a wavelength of 450 nm.

### Transwell assay

To evaluate cell migration, we initiated the procedure by adjusting the cell concentration to 2 × 10^5 cells/mL. Then, 150 μL of this cell suspension was delicately added to the upper chamber of the Transwell apparatus, while the lower chamber of each well received 800 μL of DMEM medium supplemented with 20% FBS. This setup was then placed in a controlled environment at 37°C with 5% CO2 for 24 hours. After the designated incubation period, non-migrating cells on the upper membrane surface were gently removed using sterile cotton swabs. Following this, the migrated cells were fixed with methanol for 15 minutes and then stained with crystal violet for 30 minutes. Subsequently, thorough rinsing with PBS ensured the removal of excess dye. The inserts were then subjected to microscopic examination. Five random fields of view were selected for imaging, facilitating the enumeration of migrated cells. These images provided a basis for quantifying and comparing the migratory capacities of the transfected cells.

### Wound healing test

To conduct the wound healing assay, CC cells were first grown in six-well plates with complete medium until reaching 95% confluence over a 24-hour period. Subsequently, a scratch was made using a 200 µl sterile pipette tip, followed by washing with phosphate-buffered saline (PBS) to eliminate cell debris. The adherent cells were then cultured in serum-free DMEM and images were captured at 0, 12 and 24 h post-scratching to assess the wound area as an indicator of cell invasion ability.

### 5-Ethynyl-20-deoxyuridine (EdU) assay

The EdU assays were performed using the BeyoClickTM EdU Cell Proliferation Kit with Alexa Fluor 555 (Beyotime, China). EdU was introduced into the cell culture medium and allowed to incubate for 2 hours. Subsequently, CC cells were fixed with 4% paraformaldehyde and permeabilized with 0.3% Triton X-100 (Servicebio, China). The Click solution was then prepared according to the manufacturer's instructions and used to incubate the cells for 30 minutes in the absence of light. The cells were stained with Hoechst 33342 and visualized using an inverted fluorescence microscope (Carl Zeiss, Germany).

### Statistical analysis

Statistical analyses were performed utilizing R software (version 4.0.5), GraphPad Prism 8 (GraphPad, La Jolla, CA, USA), and SPSS 26.0 (IBM, SPSS, Chicago, USA). Unpaired Student's t-tests were employed for comparing data between two groups, with statistical significance set at a threshold of P <0.05. Each experiment was replicated a minimum of three times.

## Results

### Necroptosis promotes the proliferation and invasion of CC cells

**Figure 1:**
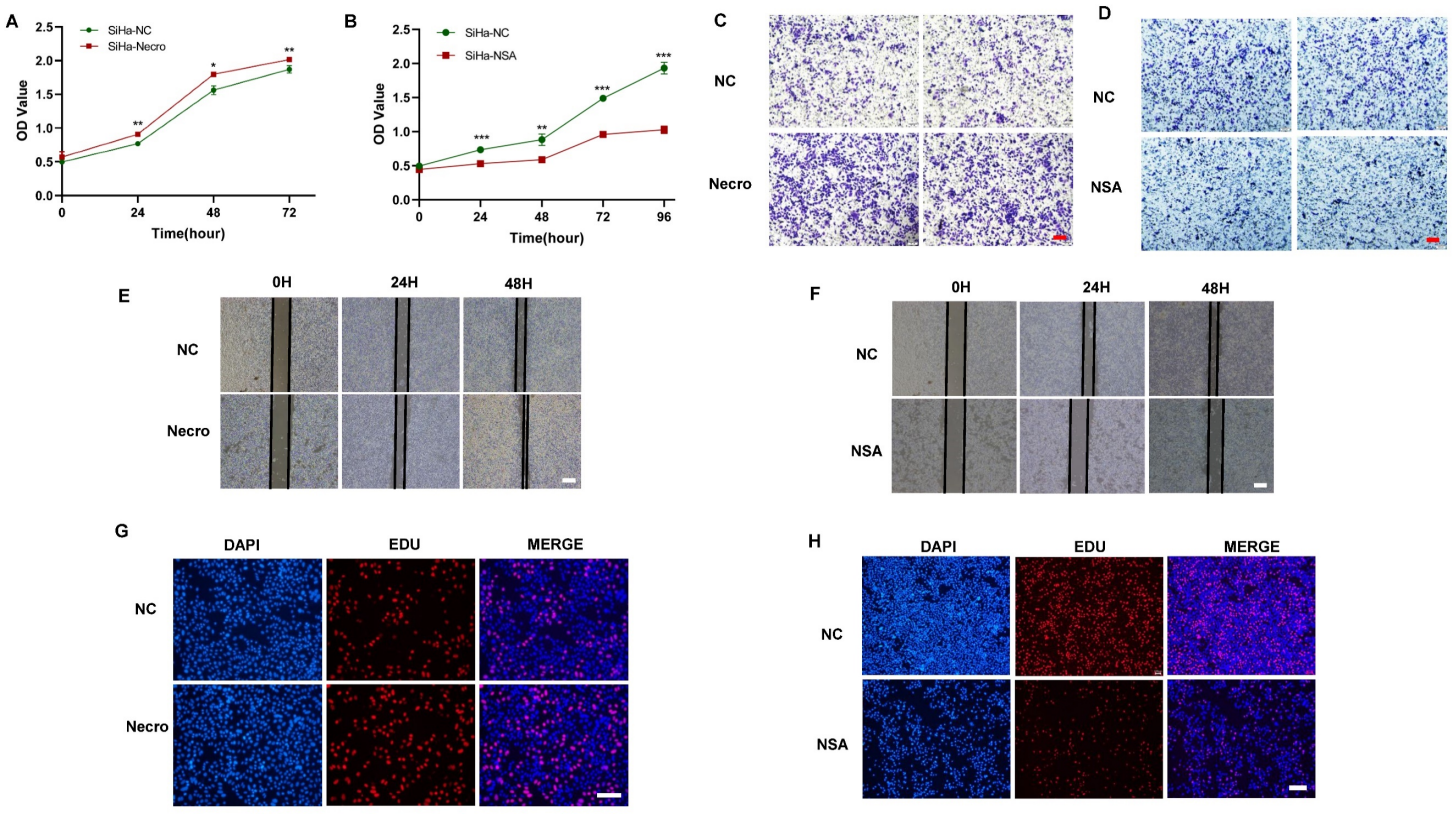
Necroptosis promotes the proliferation and invasion of CC cells. **(A)** Results of CCK-8 assays showed that at times of 24, 48 and 72h, the SiHa cells living in the necroptosis group are always significantly greater than the normal control group (p-value: ** p < 0.01, * p <0.05). **(B)** Results of CCK-8 assays showed that at times of 24, 48, 72h and 96h, the SiHa cells living in the normal control group are always significantly greater than the NSA group (p-value: *** p < 0.001, ** p < 0.01). **(C, D)** Transwell assay was performed to determine the cell migration ability of each group of cells. Scale bar, 100 μm. **(E)** Wound healing assays accessed the differences in the migration ability of SiHa cells between the necroptosis group and the normal control group. Scale bar, 100 μm. **(F)** Wound healing assays to access the differences in the migration ability of SiHa cells between the NSA group and the normal control group. Scale bar, 100 μm. **(G, H)** EdU assays were used to detect the proliferation ability of each group of SiHa cells. Scale bar, 200 μm.

We first investigated the effect of necroptosis on the proliferation and migration of CC cells by in vitro experiments. As shown by the CCK-8 results, CC cells induced to undergo necroptosis showed a higher viability compared to the normal control group (**Figure 1A**), whereas there was a decreased state with the treatment of necroptosis inhibitor (**Figure 1B**). Transwell assays were performed to assess the migration ability of CC cells and the results showed a greater migration potential in the necroptosis-inducing group (**Figure 1C**). Consistently, compared with the normal control group, necroptosis-inhibiting group exhibited a decreased migration state (**Figure 1D**). Wound healing assays were utilized to assess the migration ability of CC cells. As shown in **Figure 1E, F**, the necroptosis-inducing group tended to have a significantly increased migration ability while a weaker condition was found in the necroptosis-inhibiting group. Additionally, as depicted by the EdU results, CC cells induced into necroptosis displayed elevated proliferation ability relative to the normal control group (**Figure 1G**), while treatment with a necroptosis inhibitor led to decreased proliferation (**Figure 1H**). All these results indicated necroptosis contributed to the proliferation and invasion of CC cells. Furthermore, the intricate relationships between necroptosis and key cellular pathways were explored, shedding light on how necroptosis influences various facets of CC progression. Specifically, necroptosis disrupted DNA damage repair mechanisms, resulting in the downregulation of crucial DNA damage-related genes (PARP2, MSH2, MSH6, ERCC2, ERCC3, and POLB), culminating in genomic instability. This instability, in turn, has fueled the proliferation and invasion of tumor cells (**Figure S1A**). Moreover, necroptosis exerted its influence on cell cycle regulation, leading to the upregulation of genes such as CCND2 and CCND3 that propel cell cycle progression (**Figure S1B**). Furthermore, the induction of EMT-related genes like CLDN1 and KRT17 by necroptosis enhanced the invasive properties of CC, promoting immune evasion and resistance to therapy (**Figure S1C**). Our findings underscore the multidimensional role of necroptosis in driving the onset and advancement of CC.

### Necroptosis up-regulates VEGFA via the JAK2-STAT3 signaling pathway in CC cells

**Figure 2:**
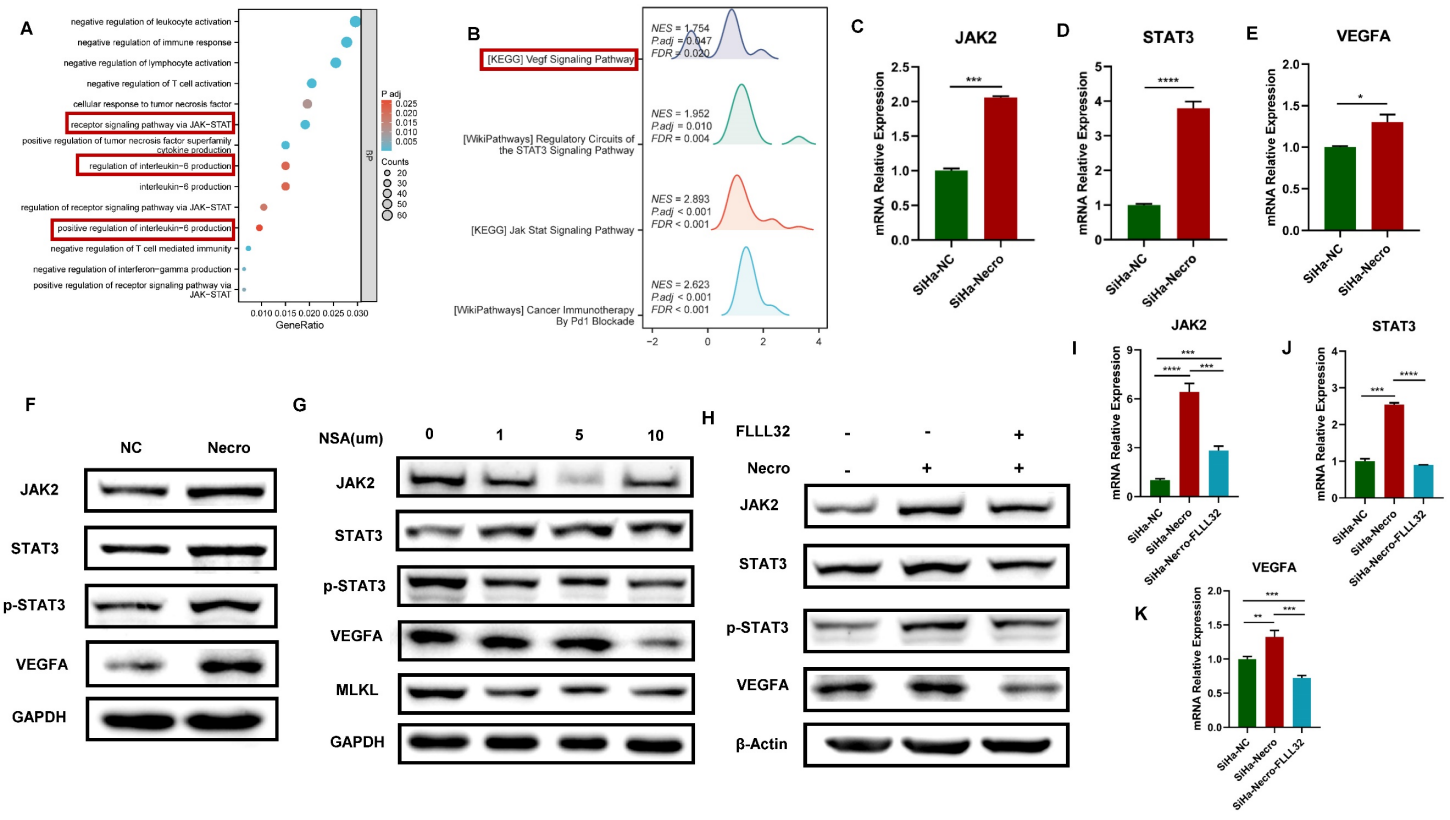
Necroptosis up-regulates VEGFA via the JAK2-STAT3 signaling pathway in CC cells. **(A)** The result from GO enrichment analysis of NRGs. **(B)** The result from KEGG analysis of NRGs. C-E RT-qPCR results indicated the up-regulation of JAK2 **(C)**, STAT3 **(D)** and VEGFA **(E)** respectively with the induction of necroptosis. “SiHa-NC” means normal control, “SiHa-Necro” means treated with necroptosis inducer at a concentration of 20 μm (p-Value: **** p < 0.0001, *** p < 0.001, * p <0.05). **(F)** Results of the WB assay showed an elevated protein level of JAK2, STAT3, p-STAT3 and VEGFA with the induction of necroptosis. “NC” means normal control, “Necro” means treated with necroptosis inducer at a concentration of 20 μm. **(G)** Results of the WB assay showed a decreased protein level of JAK2, STAT3, p-STAT3, VEGFA and MLKL with the treatment of a necroptosis inhibitor NSA. “NC” means normal control, “NSA” means treated with NSA. **(H)** Results of the WB assay indicated the up-regulation of JAK2, STAT3, p-STAT3 and VEGFA was eliminated by the specific inhibitor of JAK2-STAT3 pathway FLLL32. I-K RT-qPCR analysis revealed that the induction of necroptosis led to an increase in the expression levels of JAK2 **(I)**, STAT3 **(J)**, and VEGFA **(K)**, which were subsequently normalized upon the introduction of FLLL32. “SiHa-NC” means normal control, “SiHa-Necro” means treated with necroptosis inducer at a concentration of 20 μm, “SiHa-Necro-Flll32” means treated with necroptosis inducer (20 μm) and FLLL32 (10 μm) (p-Value: **** p < 0.0001, *** p < 0.001, ** p < 0.01).

GO enrichment analysis and KEGG analysis were performed to further explore the mechanism of necroptosis in CC cells. Results of GO enrichment analysis revealed functions of the biological process of NRGs including receptor signaling pathway via JAK-STAT and positive regulation of interleukin-6 (IL-6) production (**Figure 2A**). Results of KEGG showed NRGs were enriched in vascular endothelial growth factor (VEGF) signaling pathway and JAK-STAT signaling pathway as well (**Figure 2B**). Notably, researches have demonstrated that activation of IL-6/IL-6R up-regulates VEGF, thereby promoting tumor angiogenesis 33,34. Moreover, it was verified that the inhibition of STAT3 resulted in a decrease in VEGF 35. In this study, the WB assay showed a significant elevation of RIPK1, phosphorylated RIPK1 (p-RIPK1), RIPK3, p-RIPK3, MLKL and p-MLKL (**Figure S2A**), indicating CC cells were undergoing necroptosis with the addition of necroptosis inducer. Results of RT-qPCR also demonstrated a similar elevation in the expression levels of RIPK1 and MLKL in CC cells in the presence of the necroptosis inducer (**Figure S2B**). Besides, it was noteworthy that CC cells undergoing necroptosis exhibited an elevated protein level of VEGFA (**Figure S2C**). Results of bioinformatic analyses indicated the expression level of VEGFA was higher in advanced CC samples (**Figure S2D**) and the high expression group demonstrated a tendency towards a worse primary therapy outcome (**Figure S2E**). In addition, CC patients with lower expression of VEGFA tended to possess a better outcome, with longer disease-free survival (DFS), overall survival (OS) and progress-free survival (PFS) (**Figure S2F-H**). Furthermore, CC patients with high expression level of VEGFA and high necroptosis were more likely to experience unfavorable outcomes (**Figure S2I**). Subsequently, the mechanism of necroptosis up-regulating VEGFA was further explored through in vitro experiments. Results of RT-qPCR showed with the induction of necroptosis, the expression levels of JAK2, STAT3 and VEGFA got significantly elevated (**Figure 2C-E**). Consistently, as results of the WB assay presented, the protein levels of JAK2, STAT3, p-STAT3 and VEGFA were up-regulated with the induction of necroptosis (**Figure 2F**) whereas they were found to decrease with the treatment of NSA (**Figure 2G**). FLLL32 (MCE, HY-100544) was a specific inhibitor of the JAK2-STAT3 pathway, JAK2, STAT3 and p-STAT3 were found to be inhibited in CC cells at a concentration of 10 μm (**Figure S2J**). Results of in vitro experiments demonstrated that the up-regulation of JAK2, STAT3 and VEGFA by necroptosis inducer was eliminated by FLLL32 (**Figure 2H-K**). Therefore, it could be concluded that necroptosis up-regulates VEGFA via the JAK2-STAT3 signaling pathway.

### Bevacizumab induces the necroptosis of CC cells and activates the JAK2-STAT3 signaling pathway

**Figure 3:**
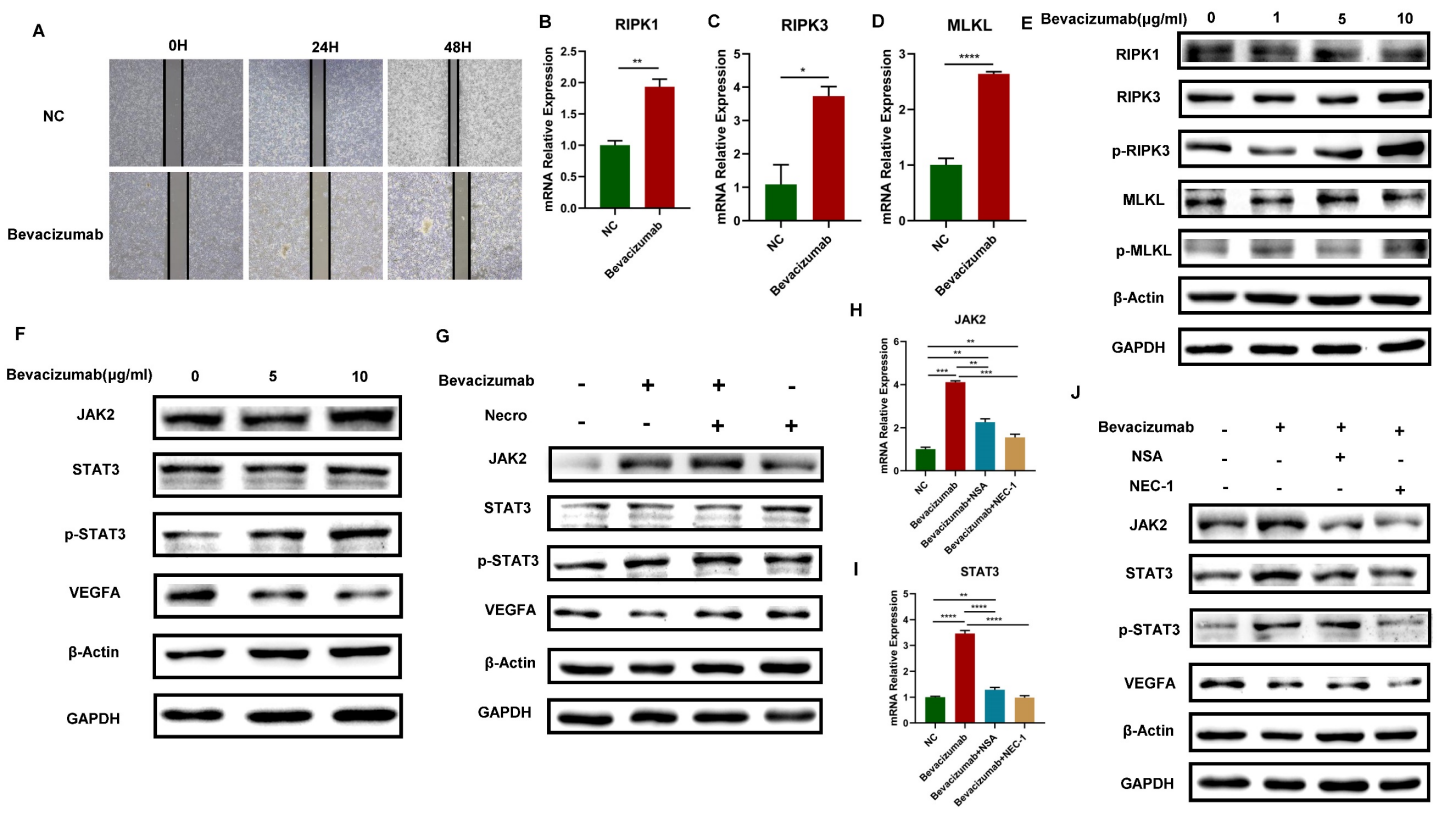
Bevacizumab induces the necroptosis of CC cells and activates the JAK2-STAT3 signaling pathway. **(A)** Wound healing assays accessed the differences in the migration ability of SiHa cells between the normal control group and the Bevacizumab group. Scale bar, 100 µm. “NC” means normal control, “Bevacizumab” means treated with bevacizumab (10 µg/ml). **(B-D)** RT-qPCR results indicated an elevated level of RIPK1 **(B)**, RIPK3 **(C)** and MLKL **(D)** respectively in the Bevacizumab (10 µg/ml) group. **(E)** Result of the WB assay indicated an increased protein level of RIPK1, RIPK3, p-RIPK3, MLKL and p-MLKL with the treatment of bevacizumab. **(F)** Result of WB showed an increased protein level of STAT3, p-STAT3 and a decreased protein level of VEGFA with the treatment of bevacizumab. **(G)** WB assay demonstrated the inhibition of VEGFA by bevacizumab (10 µg/ml) was eliminated by necroptosis inducer. **(H, I)** RT-qPCR showed the up-regulation of JAK2 **(H)** and STAT3 **(I)** by bevacizumab (10 µg/ml) was eliminated by the necroptosis inhibitor NSA or NEC-1 (p-Value: **** p < 0.0001, *** p < 0.001, ** p < 0.01). J WB indicated an up-regulation protein level of JAK2, STAT3 and p-STAT3 by bevacizumab (10 µg/ml) was eliminated by the necroptosis inhibitor NSA or NEC-1, bevacizumab and necroptosis inhibitor showed synergistic effect in the inhibition of VEGFA.

Bevacizumab is a monoclonal antibody that can specifically target VEGF. It blocks the binding of VEGF and its receptor, inhibiting tumor neovascularization and thus playing an anti-tumor role. In this study, it has been proved that necroptosis up-regulates VEGFA via the JAK2-STAT3 signaling pathway, we then further explore the interaction of necroptosis, bevacizumab and JAK2-STAT3 signaling pathway. The results of the wound healing test demonstrated that bevacizumab (Selleck, A2006) exhibited a notable inhibitory effect on the migratory capacity of CC cells (**Figure 3A**). The expression levels of RIPK1, RIPK3, MLKL and the protein levels of p-RIPK3 and p-MLKL exhibited an elevated trend when bevacizumab was added in CC cells (**Figure 3B-E**), indicating that bevacizumab induces the necroptosis of CC cells. Moreover, with the addition of bevacizumab, results of the WB assay verified the VEGFA was successfully inhibited, accompanied by a significant increase in the protein levels of JAK2, STAT3 and p-STAT3 (**Figure 3F**). The elevated protein levels of JAK2, STAT3, and p-STAT3 were observed in CC cells treated with bevacizumab alone or in combination with necroptosis inducer, however, the inhibitory effect of bevacizumab on VEGFA was eliminated in combination with necroptosis inducer (**Figure 3G**). Results of RT-qPCR showed the expression level of JAK2 and STAT3 got up-regulated with the addition of bevacizumab and the effect was reversed by necroptosis inhibitor NSA or NEC-1 (**Figure 3H-I**). Consistently, the impact of bevacizumab on JAK2, STAT3 and p-STAT3 was eliminated by NSA or NEC-1. A synergistic inhibition effect on VEGFA was observed while treated with bevacizumab in combination with NSA or NEC-1 (**Figure 3J**). All these results demonstrated that bevacizumab induced the necroptosis of CC cells, thus activating the JAK2-STAT3 signaling pathway and up-regulated VEGFA in combination with necroptosis inducer, suggesting a potential mechanism of bevacizumab resistance.

### Comprehensive bioinformatical analyses revealed necroptosis could enhance T cell signaling pathways in CC

**Figure 4:**
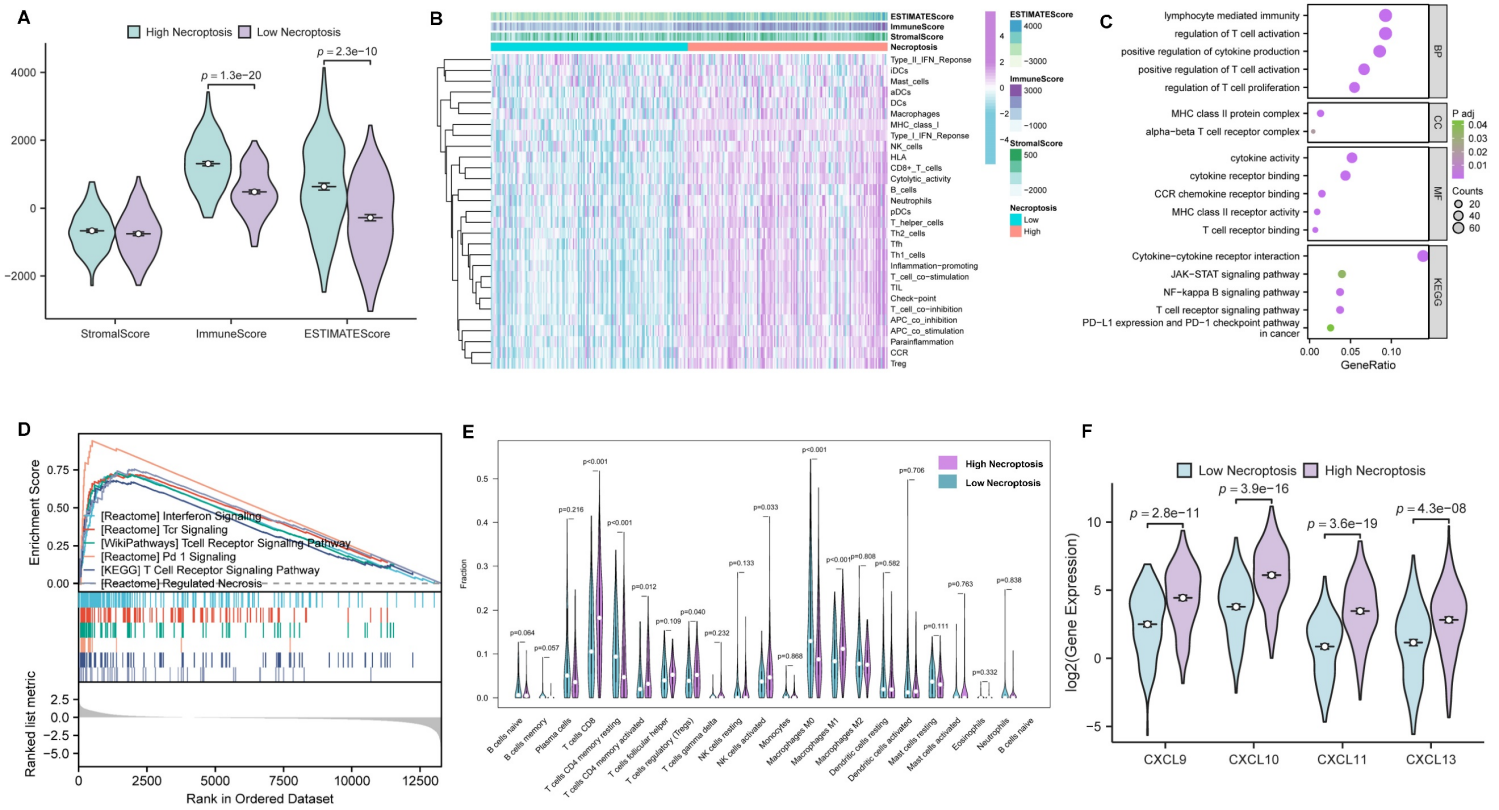
Comprehensive bioinformatical analyses revealed necroptosis could enhance T cell signaling pathways in CC. **(A)** Box plot showed the distributions of ESTIMATE-related scores between CC tumor samples with high or low expression relative to the median of NRGs expression, and Wilcoxon rank sum was applied for the significance test. **(B)** Heatmap for immune responses based on ssGSEA among NRGs high- and low-expression group. **(C)** The result from GO and KEGG analyses of NRGs. **(D)** GSEA analysis was done under the high-expression cluster. **(E)** The CIBERSORT result was shown in the comparison between low- and high-expression groups by violin plot. **(F)** Chemokine genes including CXCL9, CXCL10, CXCL11 and CXCL13 with significantly expressed differences are expressed higher in the cluster with high expression of NRGs, Wilcoxon rank sum was applied for the significance test.

In order to further investigate the role of necroptosis in TME, CC cases were first clustered into high- and low- necroptosis cohorts, with the median expression of NRGs serving as the cut-off point. Then, the correlations of TME components between the two clusters were evaluated by the ESTIMATE algorithm (**Figure 4A**). As results presented, ImmuneScore exhibited the most remarkable difference, implying CC patients with higher necroptosis enjoy a heater immune microenvironment. For investigating the correlation between necroptosis and immunologic functions as well as immune cell infiltration, ssGSEA was conducted and the results demonstrated that most immune-related functions showed a remarkable difference between high- and low- necroptosis groups (**Figure 4B**). In addition, GO enrichment analysis and KEGG analysis (**Figure 4C**) were utilized to evaluate the function of necroptosis, and results indicated that genes' functions about the biological process (BP), cellular component (CC), molecular function (MF) were all tightly centered around T cell regulation. Notably, results of KEGG revealed that necroptosis was not only involved in the T cell receptor signaling pathway but also related to the JAK-STAT signaling pathway. Moreover, GSEA was performed to investigate differences in enriched pathways between these two groups. In the high necroptosis cluster, pathways were significantly enriched in immunological functions including T cell receptor signaling pathway (**Figure 4D**). CIBERSORT was utilized to compare the difference of tumor-infiltrating cells proportion between different clusters. Notably, as the result exhibited, the high-necroptosis cohort tends to own a higher infiltration level of CD8+ T cells (**Figure 4E**). Moreover, anti-tumor chemokine genes including CXCL9, CXCL10, CXCL11 and CXCL13 expressed higher in the high-necroptosis cohort (**Figure 4F**). All these results suggested that necroptosis could elicit immune responses and dynamically change the TME of CC, particularly T cell signaling pathway.

### Necroptosis could facilitate T cell activation, reduce T cell depletion and increase T cell infiltration in vitro

**Figure 5:**
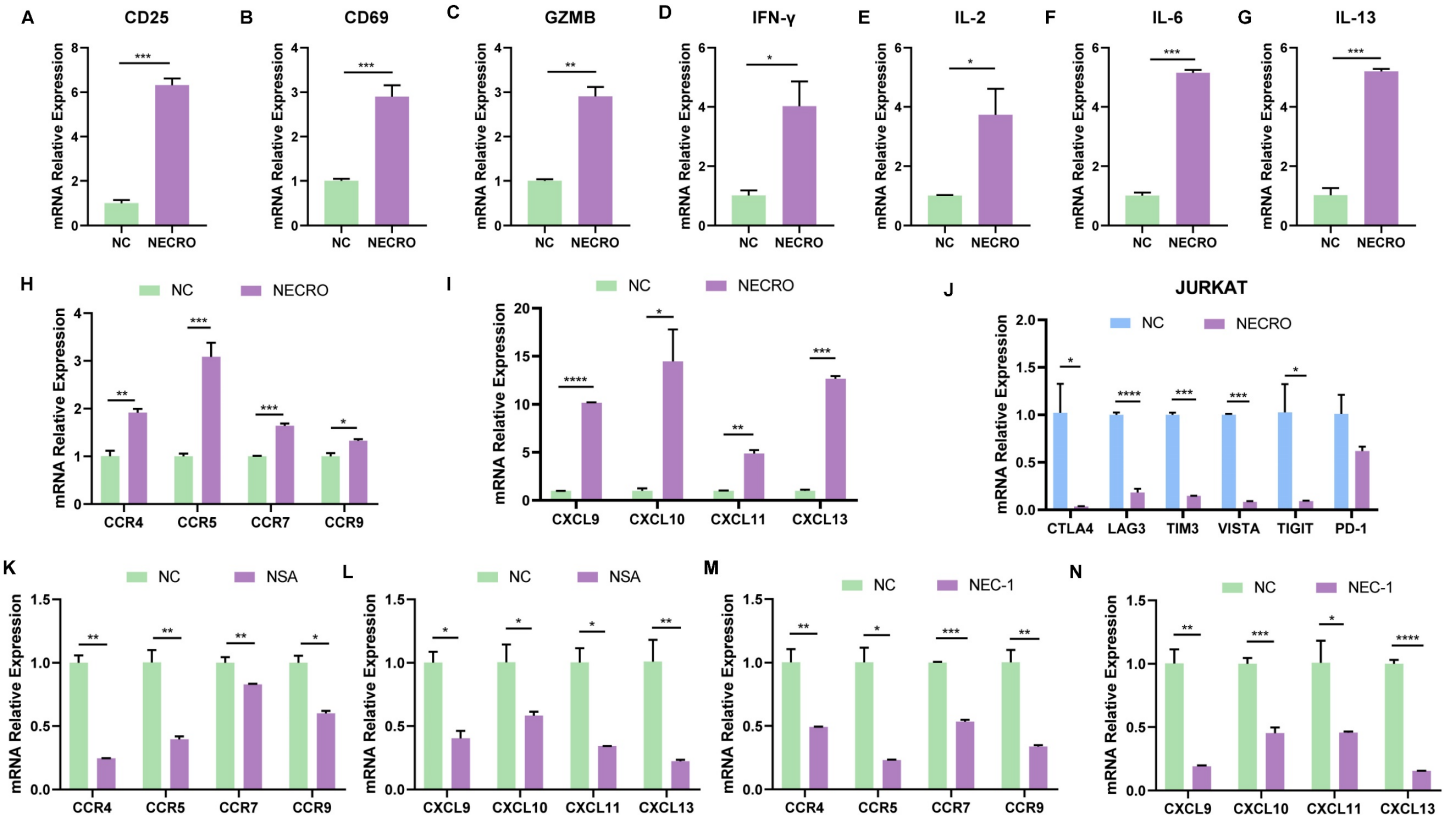
Necroptosis could facilitate T cell activation, reduce T cell depletion and increase T cell infiltration in vitro. **(A-D)** Inferred from RT-qPCR, T cell activation markers CD25 **(A)**, CD69 **(B)**, GZMB **(C)** and IFN-γ **(D)** showed correspondingly high expression with the induction of necroptosis. “NC” means normal control, “NECRO” means treated with necroptosis inducer at a concentration of 20 µm (p-Value: *** p < 0.001, ** p < 0.01, * p <0.05, ns p > 0.05). **(E-G)** RT-qPCR results showed cytokine genes IL-2 **(E)**, IL-6 **(F)** and IL-13 **(G)** were up-regulated with the induction of necroptosis (p-Value: *** p < 0.001, ** p < 0.01, * p <0.05, ns p > 0.05). **(H-J)** Treated with the induction of necroptosis, RT-qPCR results showed the expression of chemokine receptor genes CCR4, CCR5, CCR7, CCR9 **(H)** and chemokine genes CXCL9, CXCL10, CXCL11, CXCL13 **(I)** got elevated (p-Value: *** p < 0.001, ** p < 0.01, * p <0.05, ns p > 0.05). **(J)** RT-qPCR results indicated that cultured with the induction of necroptosis, immune checkpoint CTLA4, LAG3, TIM3, VISTA, TIGIT and PD-1 were down-regulated (p-Value: *** p < 0.001, ** p < 0.01, * p <0.05, ns p > 0.05). **(K, L)** Results showed the expression of chemokine receptor genes CCR4, CCR5, CCR7, CCR9 **(K)** and chemokine genes CXCL9, CXCL10, CXCL11, CXCL13 **(L)** were decreased in the condition of Necrosulfonamide (NSA) at a concentration of 10 µm. “NC” means normal control, “NSA” means treated with NSA (p-Value: *** p < 0.001, ** p < 0.01, * p <0.05, ns p > 0.05). **(M, N)** Results showed the expression of chemokine receptor genes CCR4, CCR5, CCR7, CCR9 **(M)** and chemokine genes CXCL9, CXCL10, CXCL11, CXCL13 **(N)** were decreased in the condition of Necrostatin-1 (NEC-1) at a concentration of 10 µm. “NC” means normal control, “NEC-1” means treated with NEC-1 (p-Value: *** p < 0.001, ** p < 0.01, * p <0.05, ns p > 0.05).

After identifying that necroptosis was linked to immunological processes especially CD8+ T cell regulation based on bioinformatical analyses, a series of in vitro experiments were conducted to confirm it. T cells are rapidly activated after encountering antigen stimulation and undergo a series of cytological changes such as the expression of some membrane surface molecules and synthesizing cytokines. CD69 is the earliest membrane molecule expressed by activated T cells, which can be measured and reach a peak soon after half an hour of activation, thus, it can be the earliest activation marker for T cells 36. The interleukin-2 receptor alpha subunit (IL-2Rɑ, CD25) is upregulated within 24 h of stimulation and remains elevated for several days, which can serve as a mid-late activation marker. Besides, CD25 plays a crucial role in the reactivity to IL-2, allowing T lymphocyte activation and further IL-2 production 37. Activated CD8+ T cell secretes granzyme B (GZMB), interferon-gamma (IFN-γ) which can meditate target cell lysis and apoptosis 38,39, therefore, GZMB and IFN-γ are also important indicators of T cell activation. To verify whether necroptosis influences T cell activation, RT-qPCR was performed to evaluate the changes of T cell activation markers with or without the induction of necroptosis in Jurkat cells (**Figure 5A-D**). It was obvious that the expression levels of CD25, CD69, GZMB and IFN-γ in Jurkat cells got significantly elevated with the induction of necroptosis, suggesting necroptosis facilitates T cell activation. Moreover, cytokines secreted by T cells are involved in controlling many different cellular functions, including proliferation, differentiation and cell apoptosis. In our study, results of RT-qPCR (**Figure 5E-G**) illustrated that cytokines including IL-2, IL-6, and IL-13 were in a higher expression of Jurkat cells induced with a necroptosis-inducing agent, implying that necroptosis modulated T cell proliferation and differentiation. Chemokines play a crucial role in guiding immune cell migration, which is essential to initiate and deliver an effective anti-tumor immune response. Here, we found Jurkat cells undergoing necroptosis exhibited an up-regulated expression level of chemokine receptor genes including C-C Motif Chemokine Receptor 4 (CCR4), CCR5, CCR7, CCR9 and chemokine genes including CXCL9, CXCL10, CXCL11, CXCL13 were up-regulated (**Figure 5H, I**). We also evaluated the relationship between the expression of immune checkpoints and necroptosis, results showed CTLA4, LAG3, TIM3, VISTA, TIGIT and PD-1 were down-regulated in Jurkat cells with the induction of necroptosis (**Figure 5J**), demonstrating that necroptosis might reduce T cell depletion. In contrast, while treated with necroptosis inhibitor NSA or NEC-1, the expression of CCR4, CCR5, CCR7, CCR9, CXCL9, CXCL10, CXCL11 and CXCL13 were obviously decreased in Jurkat cells (**Figure 5K-N**). It was remarkable that the viability of Jurkat cells was not affected whether treated with necroptosis inducer or necroptosis inhibitor NEC-1 and NSA according to the results of CCK-8 experiments (**Figure S3A-C**), indicating necroptosis affected functions of Jurkat cells but not proliferation. In contrast to its tumor-promoting role in CC cells, the aforementioned results provide compelling evidence to support the anti-tumor potential of necroptosis, which is achieved through the modulation of T-cell activation, depletion and infiltration. In conclusion, it can be posited necroptosis plays a dual role in CC.

### CD3D was identified as a hub gene and emerged as a protective factor in CC

**Figure 6:**
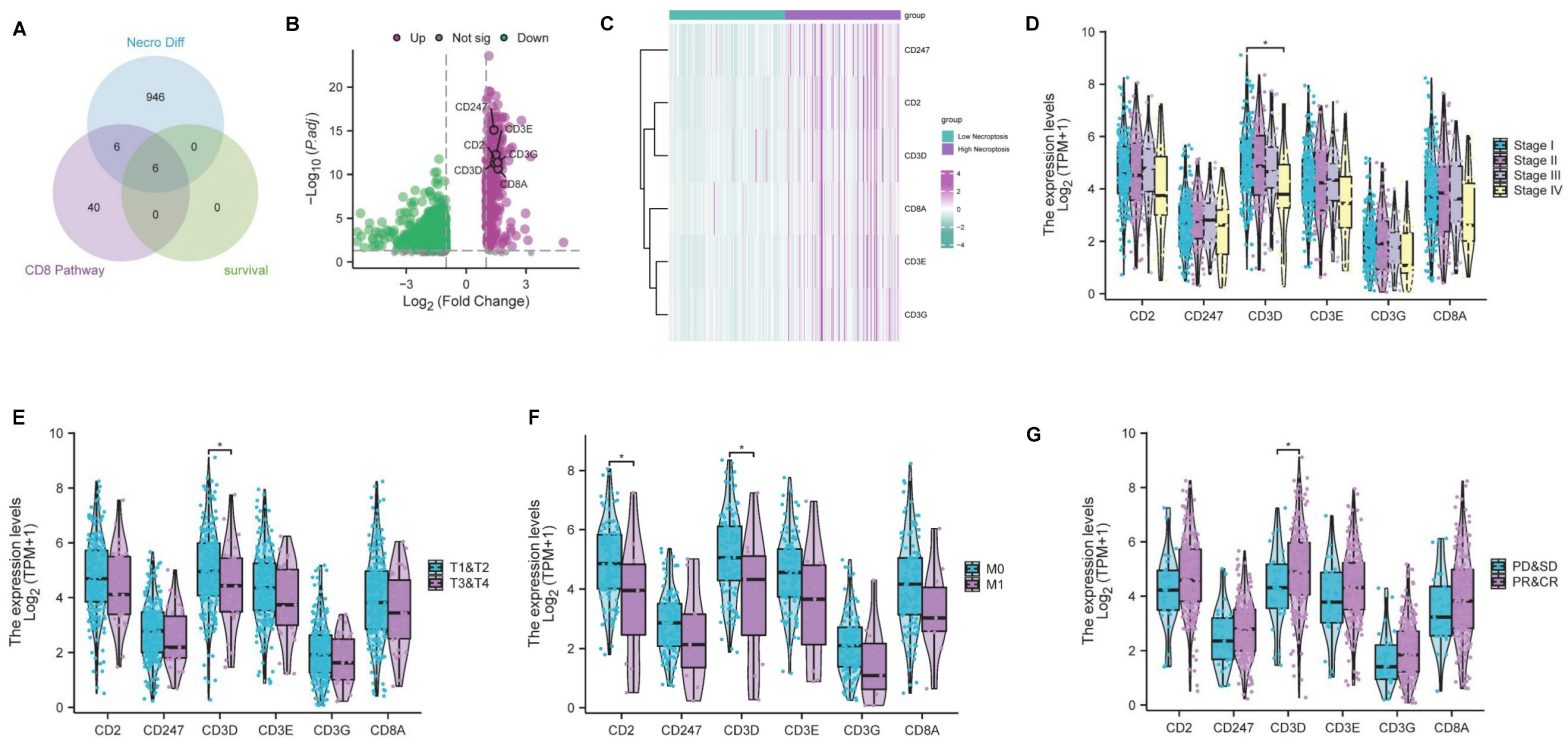
CD3D was identified as a hub gene and emerged as a protective factor in CC. **(A)** Venn plot showing six genes in the intersection analysis of differentially expressed NGRs, CD8 Pathway and prognostic-related genes. **(B)** Volcano plot for differentially expressed genes (DEGs). The green and purple dots represented the significantly downregulated and upregulated genes, respectively. FDR < 0.05, |log2 FC|> 1, and p < 0.05. **(C)** Heatmap showing the expression of six intersected genes in low necroptosis and high necroptosis clusters. **(D)** Distribution of gene expression in stage, by Kruskal-Wallis rank sum test (p-Value: *** p < 0.001, ** p < 0.01, * p <0.05, ns p > 0.05). **(E)** Distribution of gene expression in T classification, by Kruskal-Wallis rank sum test (p-Value: *** p < 0.001, ** p < 0.01, * p <0.05, ns p > 0.05). **(F)** Distribution of gene expression in M classification, by Kruskal-Wallis rank sum test (p-Value: *** p < 0.001, ** p < 0.01, * p <0.05, ns p > 0.05). **(G)** The correlation of gene expression with primary therapy response, by Kruskal-Wallis rank sum test (p-Value: *** p < 0.001, ** p < 0.01, * p <0.05, ns p > 0.05).

As previously mentioned, bioinformatic analyses and experimental investigations identified that necroptosis has played a role in the regulation of CD8+ T cells. Subsequently, we conducted a comprehensive analysis to select genes associated with both the 'CD8 Pathway' and prognosis from the pool of 958 differentially expressed NRGs (**Table S3**). This analysis led to the identification of 6 key genes (CD2, CD247, CD3D, CD3E, CD3G, CD8A) through the intersection analysis (**Figure 6A**). Remarkably, all six genes exhibited a higher expression level in the high-necroptosis cluster (**Figure 6B, C**). Subsequent correlation analysis of the expression levels of these 6 genes with clinicopathological characteristics revealed that CD3D expression was significantly linked to stage classification, grade classification, and response to primary therapy (**Figure 6D-G**). Notably, our findings indicated a negative correlation between CD3D expression level and staging as well as grading. CC patients with a high expression level of CD3D tended to be more sensitive to the primary therapy. Hence, CD3D appears to be a promising protective indicator in CC patients.

A pan-cancer analysis of CD3D expression level across various cancer types using data from TCGA was performed to investigate whether the protective effect of CD3D was limited to CC or applicable to other malignancies (**Figure S4A**). The results revealed that CD3D expression was higher in tumor cases than in normal ones across most types of cancer, suggesting its potential as a therapeutic target. Besides, survival analysis was performed to assess the prognostic significance of CD3D in CC. Results demonstrated that CC patients with high expression of CD3D tended to possess a better outcome, with longer OS, DFS and PFS (**Figure S4B-D**). Consistently, online database analysis also indicated better OS, DFS and PFS for CC patients with high CD3D levels compared to those with low levels (**Figure S4E-G**). To further explore the correlation between CD3D protein levels and clinical stage, we collected five early-stage and five late-stage CC specimens for immunohistochemical detection of CD3D protein levels. IHC results presented in Fig. S4H indicated a decrease in CD3D expression with advanced-stage classification. Consistently, correlation analysis using public databases between CD3D expression and clinicopathological characteristics revealed a lower expression level of CD3D in advanced stage and grade cases (**Figure S4I-K**). These results indicate that CD3D may function as a biomarker for predicting prognosis and stage classification in CC patients. Additionally, it was noted that individuals exhibiting elevated CD3D expression levels generally experienced more favorable outcomes following primary therapy (**Figure S4L**). These above results demonstrate that CD3D was a protective factor and potential therapeutic target in CC, which could forecast prognosis and foretell immunotherapy effects.

### Necroptosis up-regulates CD3D via the JAK2-STAT3 signaling pathway in Jurkat cells

**Figure 7:**
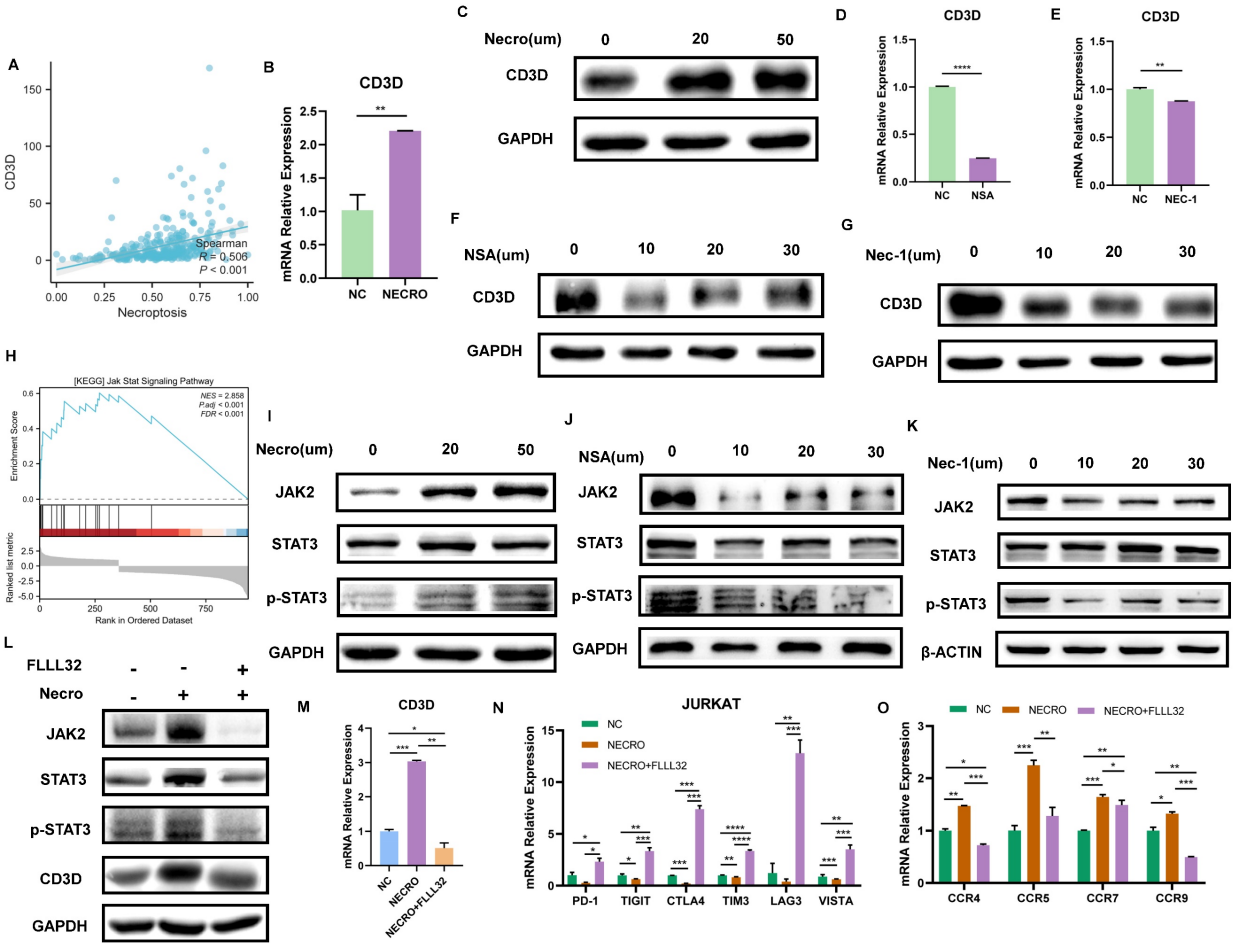
Necroptosis up-regulates CD3D via the JAK2-STAT3 signaling pathway in Jurkat cells. **(A)** The correlation analysis of CD3D expression with necroptosis level. **(B)** The expressing amount of CD3D differed in the untreated control group and necroptosis inducer treated group with a p-value <0.01(**). **(C)** The protein level of CD3D is much stronger with the induction of necroptosis and showed an increasing trend with the increase of dose. **(D, F)** Treated with NSA, Jurkat cells exhibited a lower level of CD3D evaluated by RT-qPCR, with p-value <0.0001(****) **(D)** and WB **(F)**. **(E, G)** Treated with Nec-1, Jurkat cells exhibited a lower level of CD3D evaluated by RT-qPCR, with p-value <0.01(**) **(E)** and WB **(G)**. **(H)** GSEA analysis revealed that the high necroptosis group was enriched in the JAK-STAT signaling pathway. **(I)** The protein level of JAK2, STAT3 and p-STAT3 is much stronger with the induction of necroptosis. **(J, K)** The protein level of JAK2, STAT3 and p-STAT3 exhibits to decrease in the condition of NSA **(J)** or Nec-1 **(K)**. **(L)** The protein level of JAK2, STAT3, p-STAT3 and CD3D was much stronger with the induction of necroptosis and this effect was eliminated by FLLL32. **(M)** RT-qPCR results show CD3D was up-regulated by necroptosis and this effect was inhibited by FLLL32 at a concentration of 10 µm (p-Value: *** p < 0.001, ** p < 0.01, * p <0.05, ns p > 0.05). **(N)** RT-qPCR showed the down-regulation of PD-1, TIGIT, CTLA4, TIM3, LAG3 and VISTA in the condition of TNFɑ + Z-VAD-FMK and this effect was inhibited by FLLL32 (p-Value: **** p < 0.0001, *** p < 0.001, ** p < 0.01, * p <0.05, ns p > 0.05). **(O)** RT-qPCR showed the up-regulation of CCR4, CCR5, CCR7 and CCR9 with the treatment of TNFɑ + Z-VAD-FMK and this effect was eliminated by FLLL32 (p-Value: *** p < 0.001, ** p < 0.01, * p <0.05, ns p > 0.05).

We then evaluated the relationship between CD3D and necroptosis via bioinformatical analyses and in vitro experiments. Firstly, CD3D expression was positively correlated with necroptosis, and the high expression group was found to exhibit a higher level of necroptosis, as is shown in **Figure 7A**. Given that CD3D is expressed in T cells, Jurkat cells, a T cell line, was used to conduct in vitro experiments. Consistently, with the induction of necroptosis, Jurkat cells exhibited a higher expression of CD3D compared with untreated control as the RT-qPCR result presented in **Figure 7B**, and the protein expression level of CD3D emerged higher with the increase of concentrations as the result of WB displayed in **Figure 7C**. Conversely, while treated with NSA (**Figure 7D, F**) or NEC-1 (**Figure 7E, G**), a decrease in CD3D expression was observed in Jurkat cells. These results provide valuable insights into the intricate interplay between CD3D expression and necroptosis dynamics, shedding light on potential therapeutic targets in CC patients.

As described above, our study revealed that Jurkat cells exhibited increased CD3D expression levels upon induction of necroptosis. Subsequent mechanistic investigations were conducted, with GSEA analysis indicating enrichment of the JAK-STAT signaling pathway in the high necroptosis group (**Figure 7H**). In vitro experiments were then performed to elucidate the interplay between CD3D, the JAK-STAT signaling pathway, and necroptosis in Jurkat cells. The protein levels of JAK2, STAT3, and p-STAT3 were upregulated in response to necroptosis induction, as demonstrated by our results (**Figure 7I**), while their levels decreased in the presence of NSA (**Figure 7J**) or NEC-1 (**Figure 7K**), suggesting that necroptosis may activate the JAK2-STAT3 signaling pathway. Furthermore, western blot results (**Figure 7L**) confirmed that the protein levels of JAK2, STAT3, p-STAT3, and CD3D increased in response to necroptosis induction, and this effect was attenuated by FLLL32. RT-qPCR analysis (**Figure 7M**) indicated that CD3D expression was upregulated by necroptosis induction, an effect that was inhibited by FLLL32. Therefore, our findings suggest that necroptosis upregulates CD3D via the JAK2-STAT3 signaling pathway. Interestingly, we also observed that necroptosis downregulated the expression of common ICPs, including PD-1, TIGIT, CTLA4, TIM3, LAG3, and VISTA in Jurkat cells, an effect that was reversed by FLLL32 (**Figure 7N**). Additionally, the expression of chemokine receptors such as CCR4, CCR5, CCR7, and CCR9 in Jurkat cells was upregulated in response to necroptosis, and this effect was also abrogated by FLLL32 (**Figure 7O**). These results demonstrated that necroptosis could induce upregulation of CD3D in Jurkat cells via activation of the JAK2-STAT3 signaling pathway.

We then further investigated whether the above experimental phenomenon could be found in activated T cells. Jurkat cells were firstly activated by PMA (20ng/mL, MCE, HY-18739) and lonomycin (500ng/mL, MCE, HY-13434), which was verified by the protein level of T cell activation marker CD69 using WB, and CD69 exhibited a higher level upon the induction of necroptosis (**Figure S5A**), indicating necroptosis also facilitated T cell activation even though it's in activated T cells. Besides, the protein level of JAK2, STAT3, p-STAT3 and CD3D was found to increase in activated T cells with the induction of necroptosis (**Figure S5B**), consistent with the inactivated T cells. Conversely, while treated with NSA (**Figure S5C**) or NEC-1 (**Figure S5D**), the protein level of JAK2, STAT3, p-STAT3, CD3D and CD69 in activated T cells was found to decrease. Additionally, RT-qPCR results demonstrated that CD3D was up-regulated in activated T cells with the treatment of necroptosis inducer and this effect can be inhibited by FLLL32 (**Figure S5E**). WB results shown in Fig. S4F illustrated that in activated T cells, the protein level of JAK2, STAT3, p-STAT3, CD3D and CD69 was found to increase with the induction of necroptosis and this effect was eliminated by FLLL32. Therefore, it could be concluded that necroptosis up-regulates CD3D via the JAK2-STAT3 signaling pathway whether in activated or inactivated T cells.

### Necroptosis of T cells could inhibit the viability, migration and proliferation of CC cells

**Figure 8:**
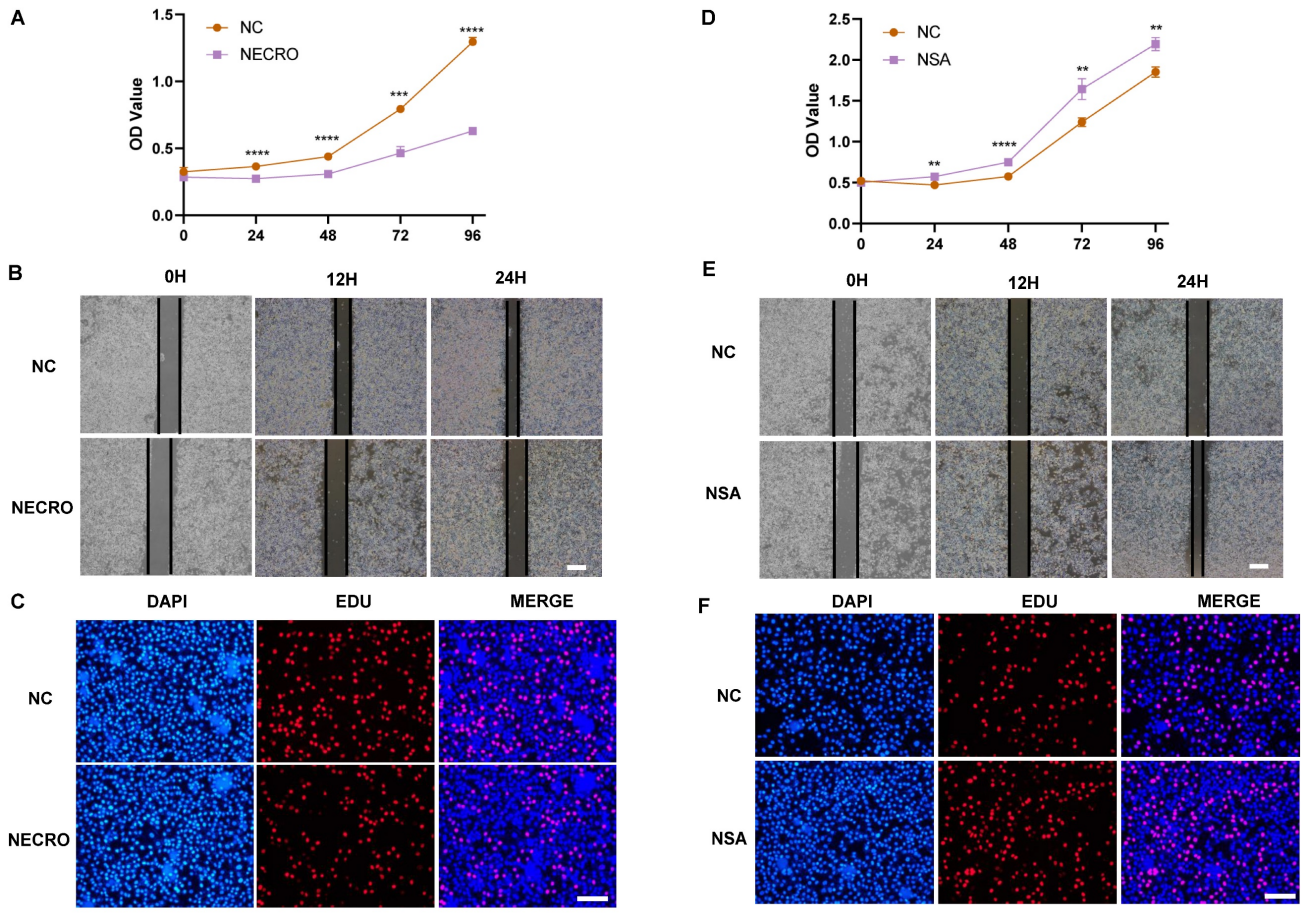
Necroptosis of T cells could inhibit the viability, migration and proliferation of CC cells. **(A)** Results of CCK-8 assays showed that at times of 24, 48, 72 and 96 h, the SiHa cells living in the normal control group are always significantly greater than the necroptosis group (p-value: *** p < 0.001, ****p <0.0001). **(B)** Wound healing assays accessed the differences in the migration ability of SiHa cells between the necroptosis group and the normal control group. Scale bar, 100 µm. **(C)** EdU assays were used to detect the proliferation ability of each group of SiHa cells. Scale bar, 200 µm. **(D)** Results of CCK-8 assays showed that at times of 24, 48, 72 and 96 h, the SiHa cells living in the NSA group are always significantly greater than the normal control group (p-value: **p <0.01, ****p <0.0001). **(E)** Wound healing assays to access the differences in the migration ability of SiHa cells between NSA group and the normal control group. Scale bar, 100 µm. **(F)** EdU assays were used to detect the proliferation ability of each group of SiHa cells. Scale bar, 200 µm.

Knowing that necroptosis facilitates T cell activation, we sought to investigate the influence of T cells undergoing necroptosis on CC cells. Jurkat cells were firstly treated with or without the induction of necroptosis and the supernatant was extracted to culture SiHa CC cells respectively. The outcomes of CCK-8 assays showed a significant suppression in the viability of SiHa CC cells cocultured with the supernatant of Jurkat cells (pretreated with TNFɑ + Z-VAD-FMK at a concentration of 20 μm the day before), as evaluated by the OD values (**Figure 8A**). As illustrated by the wound healing assays (**Figure 8B**), the necroptosis group exhibited a significant suppression of migration ability in comparison to the normal control group. Additionally, the outcomes of EdU assays further confirmed that SiHa CC cells coculturing with the supernatant of Jurkat cells (pretreated with TNFɑ + Z-VAD-FMK the day before) exhibited a decreased proliferation (**Figure 8C**). Conversely, while cocultured with the supernatant of Jurkat cells (pretreated with NSA at a concentration of 10 μm the day before), the viability, migration ability and proliferation of SiHa CC cells got elevated as demonstrated by CCK-8 (**Figure 8D**), wound healing (**Figure 8E**), and EdU assays (**Figure 8F**), respectively.

We then explored whether CC cells undergoing necroptosis would have some effects on T cells. We first treated SiHa CC cells with or without the induction of necroptosis and the supernatant was extracted to culture Jurkat cells respectively. As the RT-qPCR results shown in **Figure S6A-C**, the expression of key molecules in the necroptosis pathway including MLKL, RIPK1 and RIPK3 was found to be significantly elevated in Jurkat cells cocultured with the supernatant of SiHa CC cells (pretreated with TNFɑ + Z-VAD-FMK at a concentration of 20 μm the day before) compared with the normal control group. And the protein levels of RIPK1, p-RIPK1, RIPK3 and p-RIPK3 were also much higher (**Figure S6D**). Also, the results of RT-qPCR presented in Fig. S6E-G demonstrated that the expression of CD3D, JAK2 and STAT3 was up-regulated in the necroptosis group of Jurkat cells. Besides, the protein level of CD3D, JAK2, STAT3 and p-STAT3 detected by WB exhibit an increasing trend in the necroptosis group (**Figure S6H**). In sum, it could be inferred that CC cells undergoing necroptosis induced the expression of CD3D and up-regulated the JAK2-STAT3 signaling pathway in Jurkat cells, which might facilitate T cell activation and trigger anti-tumor immunity.

### The combination of bevacizumab with necroptosis inhibitors demonstrated an inhibitory effect on T cell function

**Figure 9:**
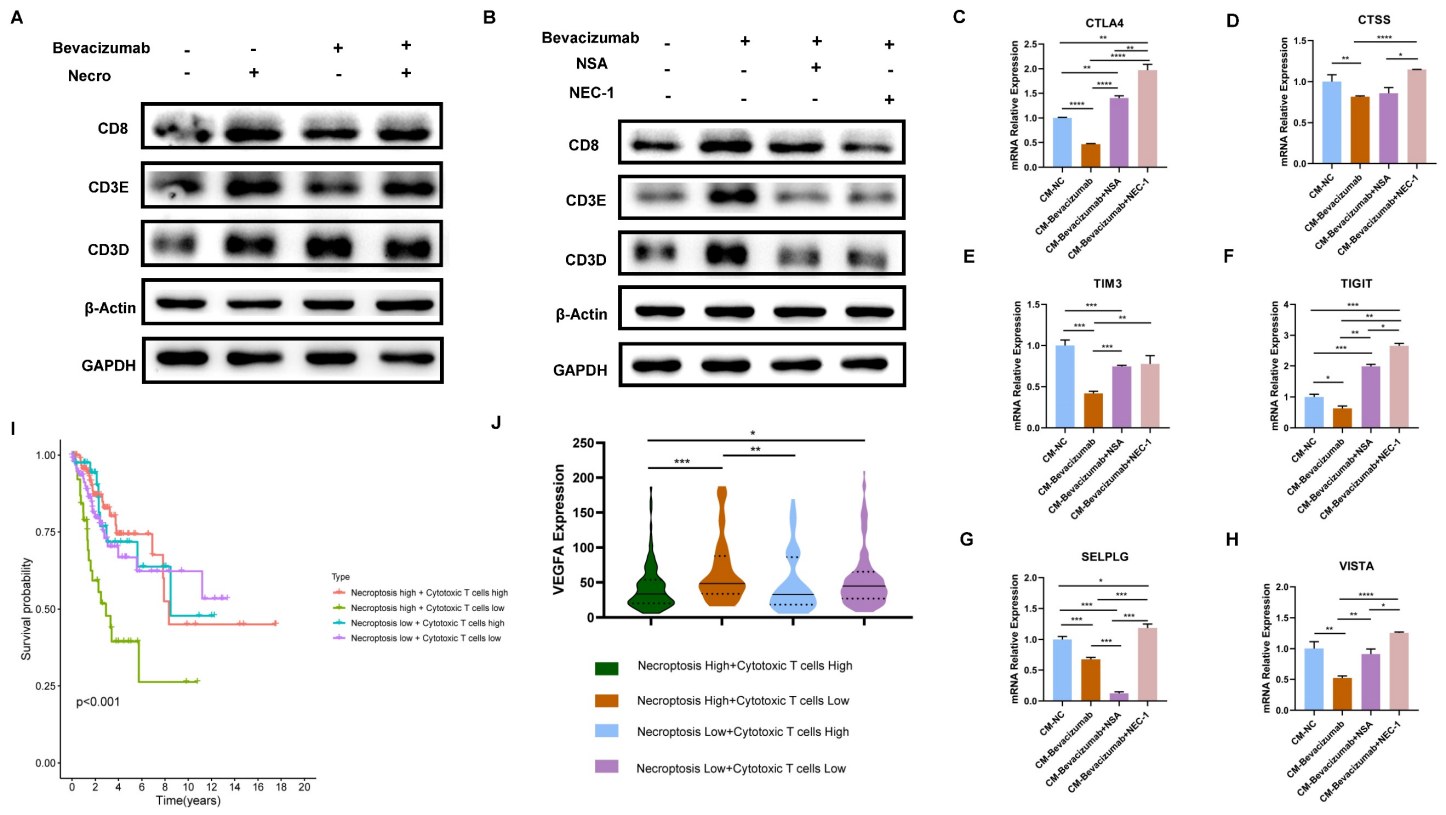
The combination of bevacizumab with necroptosis inhibitors demonstrated an inhibitory effect on T cell function. **(A)** The protein level of CD8, CD3E and CD3D was stronger with the treatment of necroptosis inducer alone or bevacizumab (10 µg/ml) alone or treated with necroptosis inducer in combination with bevacizumab. **(B)** WB assay indicated the elevation of CD8, CD3E and CD3D by bevacizumab was mitigated by the necroptosis inhibitor NSA or NEC-1. **(C-H)** RT-qPCR showed the inhibition effect of bevacizumab on CTLA4 **(C)**, CTSS **(D)**, TIM3 **(E)**, TIGIT **(F)**, SELPLG **(G)** and VISTA **(H)** was abrogated by the necroptosis inhibitor NSA or NEC-1 (p-Value: **** p < 0.0001, *** p < 0.001, ** p < 0.01, * p <0.05, ns p > 0.05). **(I)** Survival analysis revealed CC patients with high necroptosis level and low infiltration of cytotoxic T cells tended to have the worst outcome. **(J)** Bioinformatic analysis indicated the high necroptosis level and low infiltration of cytotoxic T cells group owns the highest expression level of VEGFA.

As previously demonstrated, necroptosis is a key driver of T cell activation and bevacizumab induces the necroptosis of CC cells. Subsequently, the impact of necroptosis inducer/inhibitor in conjunction with bevacizumab on TME was evaluated. In view of the fact that T cells represent the principal anti-tumor force in TME, they were selected for the following experiments. Results of the WB assay indicated in comparison to bevacizumab alone, the expression of T cell activation markers including CD8 and CD3E were found to be elevated when combined with the necroptosis inducer (**Figure 9A**). In contrast, CD8, CD3E and CD3D were observed to down-regulated in combination with necroptosis inhibitor NSA or NEC-1 (**Figure 9B**). We then treated Jurkat cells with supernatant extracted from CC cells (pretreated with bevacizumab or bevacizumab in combination with necroptosis inhibitor respectively the day before). Results of RT-qPCR showed markers of T cell inhibition and depletion including CTLA4, CTSS, TIM3, TIGIT, SELPLG and VISTA exhibited a significant elevation in the combination group (bevacizumab in combination with necroptosis inhibitor) (**Figure 9C-H**), indicating the combination of bevacizumab with necroptosis inhibitors may present a challenge in the creation of an inhibitory immune microenvironment. As the survival analysis shows, CC patients with a high necroptosis level and a low cytotoxic T cell infiltration tended to possess the poorest outcome (**Figure 9I**). Besides, this group of CC patients exhibited the highest expression level of VEGFA (**Figure 9J**). All these findings offer new insights into the potential for individualized treatment of CC. Patients with a high level of necroptosis and low infiltration of cytotoxic T cells are more likely to benefit from the bevacizumab and necroptosis inhibitor combination therapy.

## Discussion

CC appears to be one of the most lethal forms of cancer in females. Despite significant advancements in immunotherapy in recent years, the prognosis for individuals with advanced or metastatic CC continues to be unfavorable 11,40. Immunotherapy represented by ICIs exerted limited effects in advanced or metastatic CC partly caused by the immunosuppressive TME 41. Angiogenesis is a prominent characteristic of malignancies and the utilization of bevacizumab represents a novel therapeutic approach for a spectrum of advanced cancers with a dismal prognosis including advanced and metastatic CC. The combination of bevacizumab and PD-L1 inhibitor atezolizumab offers a new option for CC treatment. Nevertheless, the utilization of bevacizumab is constrained by individual variations, the emergence of drug resistance, and alterations in the TME 42-44, so there is an immediate requirement to identify reliable biomarkers to stratify patients in order to guide personalized treatment and to convert this immune “cold” tumor into a “hot” tumor that responds better to targeted therapy.

**Figure 10:**
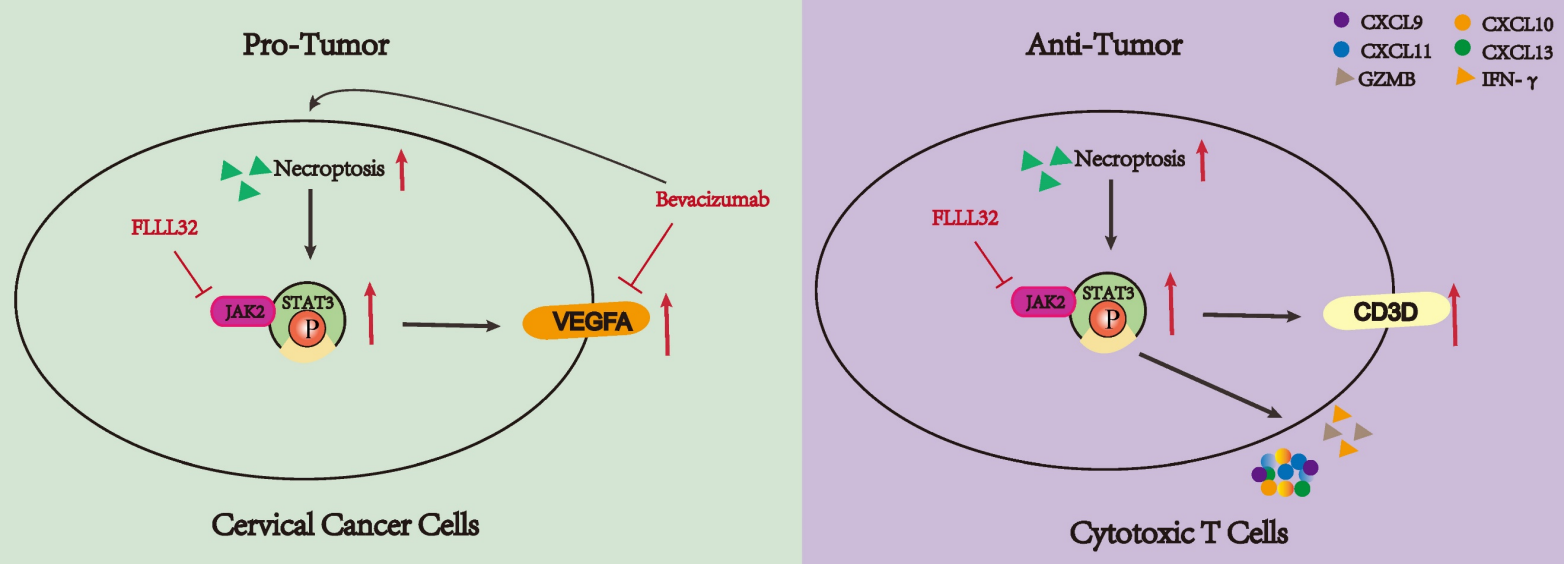
The dual functionality of necroptosis in CC patients. Necroptosis plays a dichotomous role in the progression of CC. On one hand, necroptosis facilitates the proliferation of CC cells by upregulating the JAK2-STAT3 signaling pathway and augmenting VEGFA expression. Treatment with bevacizumab in CC cells induces necroptosis, and concurrent administration of necroptosis inhibitors synergistically attenuates VEGFA expression. Conversely, necroptosis elicits T cell activation and bolsters anti-tumor immune responses through the activation of the JAK2-STAT3 signaling pathway.

Current research has revealed that the deregulation of cell death was a key factor in tumor development and progression 45. As an indispensable type of programmed cell death, necroptosis emerged as an important event that modulates tumorigenesis. Necroptosis and apoptosis are distinct yet interconnected programmed cell death pathways. Apoptosis is caspase-dependent, leading to cell shrinkage and DNA fragmentation, while necroptosis is caspase-independent, causing cell swelling and membrane rupture. Their interplay is mediated by regulators like RIPK1 and caspase-8, with caspase-8 inhibition promoting necroptosis through RIPK1-RIPK3 interaction, and its activation steering cells towards apoptosis 46-48. Ferroptosis, an iron-dependent cell death characterized by lipid peroxidation and oxidative stress, involves different regulators such as glutathione and GPX4. Oxidative stress can influence necroptosis mediators, and shared pathways like ROS and cellular redox status link ferroptosis and necroptosis, revealing a complex regulatory network 49-51. An increasing number of studies have demonstrated many compounds exhibited antitumor effects by inducing or manipulating necroptosis-related molecules 52-56. Moreover, the character of necroptotic cells shown to elicit adaptive immunity makes it a potential target for immunotherapy 57. Besides, its dual role in tumors makes it possible to stratify patients and provide an important basis for individualized treatment. Therefore, a greater understanding of necroptosis might have great use to create new strategies to control cancer. However, there are few studies on the relationship between CC and necroptosis, the role of necroptosis in the TME of CC remains unclear. Our study indicated the dual role of necroptosis in CC, promoting tumor aggression by enhancing cell proliferation through JAK2-STAT3 pathway activation while also activating the T cell activity to stimulate anti-tumor immune responses (**Figure 10**).

Based on our in vitro experiment results, necroptosis contributes to the invasion of CC cells, reflecting its pro-cancer side. The interplay between necroptosis and DNA repair processes in CC drives genomic instability, cell cycle dysregulation, and EMT induction, promoting tumor progression and therapy resistance. Targeting these interactions may offer novel therapeutic opportunities for combating CC. Enrichment analyses revealed NRGs were involved in the VEGF and JAK-STAT signaling pathway. With regard to VEGF, it has been found to be up-regulated in numerous malignant neoplasms and its combination with VEGFR has been shown to contribute to tumor development and metastasis 58. The JAK-STAT pathway, a well-studied signaling pathway, has been shown to play a crucial role in regulating cell proliferation, differentiation, and apoptosis. Furthermore, this pathway is implicated in the development of immune disorders and tumorigenesis 59. The primary role of STAT3 is transcriptional regulation, modulating angiogenesis and tumorigenesis 60. Results of the experiments indicated that necroptosis could up-regulate VEGFA via the JAK2-STAT3 signaling pathway, thereby elucidating the potential mechanism underlying its role in cancer promotion. This finding aligned with previous research, which demonstrated that STAT3 is directly bound to the VEGFA promoter and enhanced its transcriptional activity. This interaction led to increased VEGFA expression in cancer cells, thereby promoting angiogenesis and tumor growth 61-63. Interestingly, in this study, it was confirmed that bevacizumab can induce necroptosis of CC cells and subsequently activate the JAK2-STAT3 signaling pathway. The combination of bevacizumab with the necroptosis inducer resulted in a reduction in the inhibitory effect of bevacizumab on VEGFA, which may be indicative of a resistance mechanism to bevacizumab. In contrast, when bevacizumab was combined with the necroptosis inhibitor, the inhibition of VEGFA was enhanced, suggesting a new drug combination strategy: for patients resistant to bevacizumab, the combination of bevacizumab and necroptosis inhibitors could be a better therapeutic strategy, for which could reduce VEGFA expression and reduce potential resistance due to necroptosis.

In the case of tumor cells, necroptosis exerts a pro-cancer effect. However, it demonstrates a cancer-inhibiting role by activating T cells in TME. According to bioinformatic analyses, the high necroptosis group is mainly enriched in the immune response pathway, especially the T cell regulation signaling pathway. In vitro experiments verified Jurkat cells undergoing necroptosis emerged to express an elevated level of cytokine-related genes including CXCL9, CXCL10, CXCL11, CXCL13, IFN-γ, IL-2, IL-6 and IL-13. As for chemokines, the CXCL9/10/11-CXCR3 axis has been proven to promote the recruitment of immune cells into the tumor, remodeling the tumor immune microenvironment 64,65, the up-regulation of CXCL9/10/11 implies necroptosis may exert an anti-tumor effect by enhancing the infiltration of immune cells in the TME. Both IL-2, a key factor of T cell proliferation 66, and IL-6, an indispensable role in the differentiation of T cells into cytotoxic cells (CTLs) 67 were up-regulated in Jurkat cells undergoing necroptosis, indicating necroptosis plays a positive regulatory role on T cells. As the main force of anti-tumor immunity, in addition to increasing T cell infiltration, it is also crucial to promote T cell activation and reduce exhaustion. Immune checkpoints including PD-1, TIGIT, LAG3, CTLA4, TIM3 and VISTA have been affirmed to inhibit T cell activation and induce T cell depletion 68-71. According to our results, Jurkat cells undergoing necroptosis exhibited a decreasing expression level of ICPs, implying necroptosis can defend against tumors by reducing T cell depletion. And as mentioned above, T cell activated markers including CD69, CD25, IFN-γ and GZMB got significantly elevated in Jurkat cells undergoing necroptosis. Therefore, necroptosis might facilitate T cell activation, reduce T cell depletion and increase T cell infiltration, bringing heat to TME. Besides, in our research, in vitro experiments demonstrated CC cells co-cultured with supernatant from Jurkat cells undergoing necroptosis exhibited less viability and less ability to proliferate and migrate, while necroptosis exerted less effect on Jurkat cell viability. Therefore, it could be inferred that necroptosis plays a dual role in CC, which makes it a potential indicator to classify patients in order to perform precise treatment.

In this study, we obtained 6 differentially expressed NRGs related to both 'CD8 pathway' and prognosis. All these genes are characterized by the presence of cancer-suppressing properties. Among 6 intersected genes, CD247, CD3E, CD3G and CD3D were suggested to be involved in T cell development and the transduction of T cell activation signals 72. As for CD2, its encoded protein interacts with LFA3 (CD58), involving in the antigen recognition process 73. It was found that CD8a was a key molecule for the activation of cytotoxic T-lymphocytes (CTLs) 74. Therefore, all these genes exhibit a positive regulatory role in the immune response, which might explain their downward trend in advanced grade and stage. Furthermore, the results of our study demonstrated that the high necroptosis group exhibited a higher expression level of all these genes, suggesting necroptosis plays a vital role in immunity. Among these 6 selected genes, we found CD3D was most closely related to clinical features and therapy response of CC, so further analyses were around the relationship between CD3D and necroptosis.

CD3D is a component of the TCR/CD3 complex and plays an indispensable role in T cell activation signals 72. Recent research has suggested that CD3D could be a predictive biomarker of the prognosis in some cancers 75,76. In our study, bioinformatical analyses revealed CD3D was positively correlated with necroptosis, and a higher necroptosis level tended to own a “hotter” TME, therefore CD3D could be used as an indicator for dynamic detection of the immune microenvironment. Additionally, its expression was found to have a negative correlation with both grade and stage, and survival analyses have suggested that patients had a better prognosis when it was highly expressed. Besides, patients in the CD3D high expression cluster showed more sensitivity to immunotherapy. All these results indicated that CD3D acted as a protective signature in CC and it could be a reliable biomarker of prognosis as well as grade and stage classification. Simultaneously, our study indicated that the high necroptosis group was found to be enriched in the JAK-STAT signaling pathway via GSEA analysis. In addition to tumorigenesis, STAT3 has been proven to play a key role in the development of late-stage T cells and the formation of memory T cells 77,78. Another important finding in our study was that Jurkat cells undergoing necroptosis exhibited a higher level of CD3D expression and activated the JAK2-STAT3 signaling pathway. When the JAK2-STAT3 signaling pathway was specifically inhibited by FLLL32, elevated CD3D expression via necroptosis could be rescued in Jurkat cells. Thus, we conceived that necroptosis played a positive role in immunoregulation by up-regulating CD3D through activation of the JAK2-STAT3 pathway, and novel immunotherapy strategies that target this mechanism could be developed in order to improve treatment effectiveness, especially in patients who are not sensitive to conventional therapies.

An altered microenvironment is vital for the prognosis of CC patients. Previous studies have proved that VEGF can enhance the function and accumulation of MDSCs, thereby contributing to the formation of an immunosuppressive microenvironment 79,80. In this study, it was found that bevacizumab alone or in combination with necroptosis inducer induced the expression of activation markers including CD8, CD3D and CD3E in T cells. Furthermore, co-cultured with supernatant extracted from CC cells (pretreated with bevacizumab the day before), T cells exhibited a decreased expression in depletion and inhibition markers including CTLA4, CTSS, TIM3, TIGIT, SELPLG and VISTA, indicating blocking VEGF helps activate T cells thus creates a “hot” TME in CC. On the contrary, it was observed that T cells showed a decreased expression in CD8, CD3D and CD3E while treated with bevacizumab in combination with necroptosis inhibitor NSA or NEC-1. Additionally, T cells exhibited an elevation of CTLA4, CTSS, TIM3, TIGIT, SELPLG and VISTA co-cultured with supernatant extracted from CC cells (pretreated with bevacizumab in combination with necroptosis inhibitor NSA or NEC-1 the day before). The synthesis of the above results suggests that the addition of the necroptosis inhibitor could enhance the effect of bevacizumab on VEGFA blocking. However, the combination of bevacizumab and the necroptosis inhibitor might result in an immunosuppressive condition. The dual role of necroptosis foretells a potential individualized treatment strategy, CC patients with a high extent of necroptosis or an immune-depletion state are more likely to benefit from the combination of bevacizumab and the necroptosis inhibitor. Besides, both bevacizumab and necroptosis inhibitors can affect the immune microenvironment, therefore dynamic monitoring of the immune microenvironment condition is necessary, while CD3D can serve as an indicator of the degree of necroptosis and foresee the state of the immune microenvironment.

While our study highlights the potential of necroptosis as a therapeutic target in cervical cancer, several limitations and challenges need to be addressed to fully assess its feasibility and safety. First of all, the absence of in vivo validation of necroptosis in CC progression and immune modulation is one of the limitations. Future research should focus on establishing animal models to investigate these effects, elucidating the precise mechanisms underlying necroptosis-mediated CD3D upregulation, the interaction between necroptosis and bevacizumab, and the potential effects of necroptosis inhibitors combined with bevacizumab. Another significant concern is the potential for off-target effects, where the induction of necroptosis in non-cancerous tissues could lead to unintended cell death and associated toxicities. This necessitates the development of highly specific necroptosis-inducing agents that can selectively target cancer cells while sparing normal tissues. Additionally, the possibility of resistance mechanisms emerging in cancer cells poses a challenge. Tumor cells may adapt to necroptosis-based therapies through genetic or epigenetic modifications, thereby reducing treatment efficacy over time. Further research is required to understand these mechanisms and develop combination strategies that can prevent or overcome resistance. Lastly, the systemic effects of necroptosis induction, particularly on the immune system and other critical physiological processes, must be carefully evaluated. This includes assessing the impact on immune cell function and the potential for exacerbating inflammatory responses. Addressing these challenges through rigorous preclinical and clinical investigations will be crucial for translating necroptosis-targeted therapies into safe and effective treatment options for cervical cancer patients.

## Conclusion

To sum up, this study delves into the intricate role of necroptosis in the development of CC, elucidating how it stimulates an anti-tumor immune response while facilitating tumor proliferation and invasion. Moreover, a necroptosis-related molecule CD3D was identified as a protective biomarker to forecast prognosis and foretell the therapeutic effects of CC. It was also demonstrated that the T cell undergoing necroptosis could elicit an immune response by up-regulating CD3D through activation of the JAK2-STAT3 pathway, which might provide a new perspective for the regulation of the immune microenvironment of CC and might be helpful in creating novel strategies for controlling cancer. More importantly, this study proposed the potential mechanism of drug resistance to bevacizumab by exploring the interaction between necroptosis and bevacizumab, and the combination of bevacizumab with necroptosis inhibitors is expected to improve the efficacy of drug-resistant patients, providing new insights for the treatment of CC.

## Supplementary Material

Supplementary figures and tables.

## Abbreviations

CC: Cervical cancer
CTLA-4: Cytotoxic T lymphocyte-associated antigen 4
CXCL1: C-X-C Motif Chemokine Ligand 1
CCR: C-C Motif Chemokine Receptor
CCK8: Cell Counting Kit-8
CTLs: Cytotoxic T-lymphocytes
DEGs: Differentially expressed genes
DFS: Disease-free survival
FDR: False discovery rate
FC: Fold change
FIGO: International Federation of Gynecology and Obstetrics
GO: Gene Ontology
GSEA: Gene set enrichment analysis
GZMB: Granzyme B
ICIs: Immune checkpoint inhibitors
ICPs: Immune checkpoints
IHC: Immunohistochemistry
IFN-γ: Interferon gamma
KEGG: Kyoto Encyclopedia of Genes and Genomes
KM: Kaplan-Meier
MLKL: Mixed lineage kinase domain-like pseudokinase
MDSCs: Myeloid-derived suppressor cells
NRGs: Necroptosis-related genes
NSA: Necrosulfonamide
NEC-1: Necrostatin-1
OD: Optical density
OS: Overall survival
PBS: Phosphate-buffered saline
PD-1: Programmed cell death
PD-L1: Programmed cell death ligand-1
PFS: Progress free survival
RIPK1: Receptor interacting protein kinase 1
RIPK3: Receptor interacting protein kinase 3
ssGSEA: Single-sample Gene Set Enrichment Analysis
TME: Tumor microenvironment
TCGA: The Cancer Genome Atlas
VEGF: Vascular endothelial growth factor
WB: Western blotting
