# Supplementary material for: Dual Role of Necroptosis in Cervical Cancer: Promoting Tumor Aggression and Modulating the Immune Microenvironment via the JAK2-STAT3 Pathway
Source: J Cancer. 2024 Aug 13;15(16):5288–307. doi: 10.7150/jca.98738 (PMC11375541; doi:10.7150/jca.98738)
Supplement: Supplementary file 1 — Supplementary figures and tables. [file jcav15p5288s1.pdf]

Dual Role of Necroptosis in Cervical Cancer: Promoting Tumor Aggression and Modulating the Immune Microenvironment via the JAK2-STAT3 Pathway

Fangfang Xu [1], Yingjun Ye [1], Yueqing Gao [1,*], Shaohua Xu [1,*]

[1] Department of Gynecology, Shanghai Key Laboratory of Maternal Fetal Medicine, Shanghai Institute of Maternal-Fetal Medicine and Gynecologic Oncology, Shanghai First Maternity and Infant Hospital, School of Medicine, Tongji University, Shanghai, China

*Corresponding author:

Shaohua Xu

xushaohua@tongji.edu.cn

Yueqing Gao

gaoyueqing@51mch.com

Fangfang Xu and Yingjun Ye contributed equally to this work.

Supplementary Figures and Tables

Figures S1-6

Tables S1-3

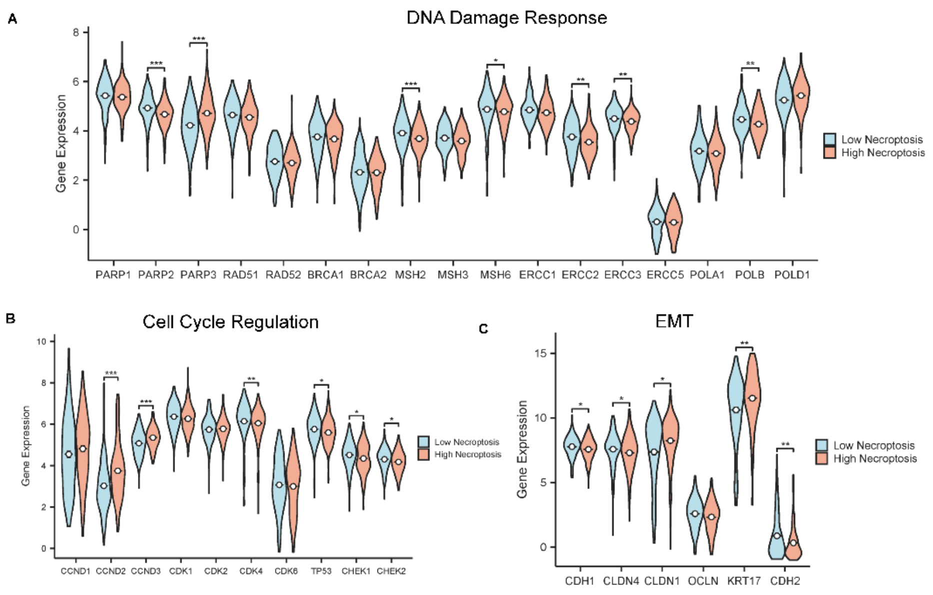

Fig. S1 (A) The expression of DNA damage response-related genes in low necroptosis and high necroptosis group in cervical cancer patients (p-Value: *** p < 0.001, ** p < 0.01, * p <0.05). **(B)** The expression of Cell cycle regulation-related genes in low necroptosis and high necroptosis group in cervical cancer patients (p-Value: *** p < 0.001, ** p < 0.01, * p <0.05). **(C)** The expression of EMT-related genes in low necroptosis and high necroptosis group in cervical cancer patients (p-Value: ** p < 0.01, * p <0.05).

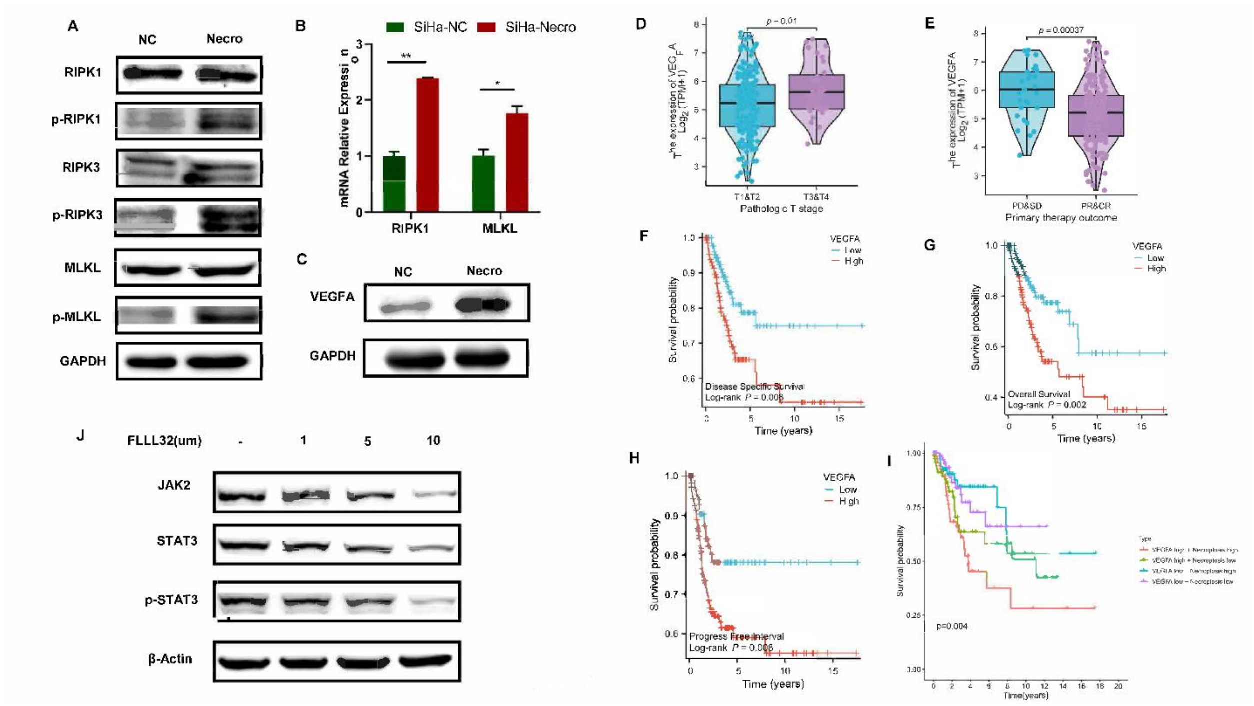

Fig. S2: In vitro experiments confirmed the induction of necroptosis and bioinformatic analyses identified the role of VEGFA in CC. (A) The protein levels of RIPK1, p-RIPK1, RIPK3, p-RIPK3, MLKL and p-MLKL in the necroptosis group were found to be significantly higher than those observed in the normal control group. “NC” means normal control, “Necro” means treated with necroptosis inducer. **(B)** RT-qPCR showed an increased expression level of RIPK1 and MLKL in the necroptosis group (p-Value: ** $p < 0.01$, * $p < 0.05$, ns $p > 0.05$). **(C)** The protein level of VEGFA was significantly elevated in the presence of the necroptosis inducer. **(D)** Distribution of gene expression in T stage classification, by Kruskal-Wallis rank sum test. **(E)** The correlation of gene expression with primary therapy outcome, by Kruskal-Wallis rank sum test. **(F-H)** The prognostic values of VEGFA expression in CC patients. Patients were marked with high expression or low expression depending on comparing with the median expression level, Kaplan-Meier (KM) survival analysis portrayed the effects of VEGFA expression on the disease-free survival (F), overall survival (G) and progress free interval (H) in TCGA-CESC cohort, p-

Value was calculated by log-rank test. **(I)** Survival analysis revealed CC patients with high expression level of VEGFA and high necroptosis were more likely to have the worst outcome. **(J)** The protein level of JAK2, STAT3 and p-STAT3 exhibited a decreased trend with the addition of FLLL32.

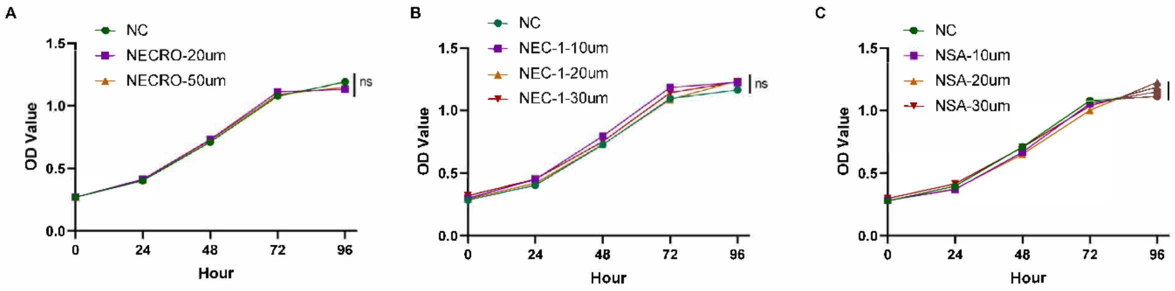

Fig. S3: Necroptosis had no effect on Jurkat cell proliferation. (A-C) Results of CCK-8 assays showed that at times of 24, 48, 72 and 96 h, the viability of Jurkat cells was not affected whether treated with necroptosis inducer (A) or necroptosis inhibitor NEC-1 (B) and NSA (C) at different concentrations (p-value: ns $p > 0.05$). "NC" means normal control, "NECRO" means treated with necroptosis inducer, "NEC-1" means treated with NEC-1, "NSA" means treated with NSA.

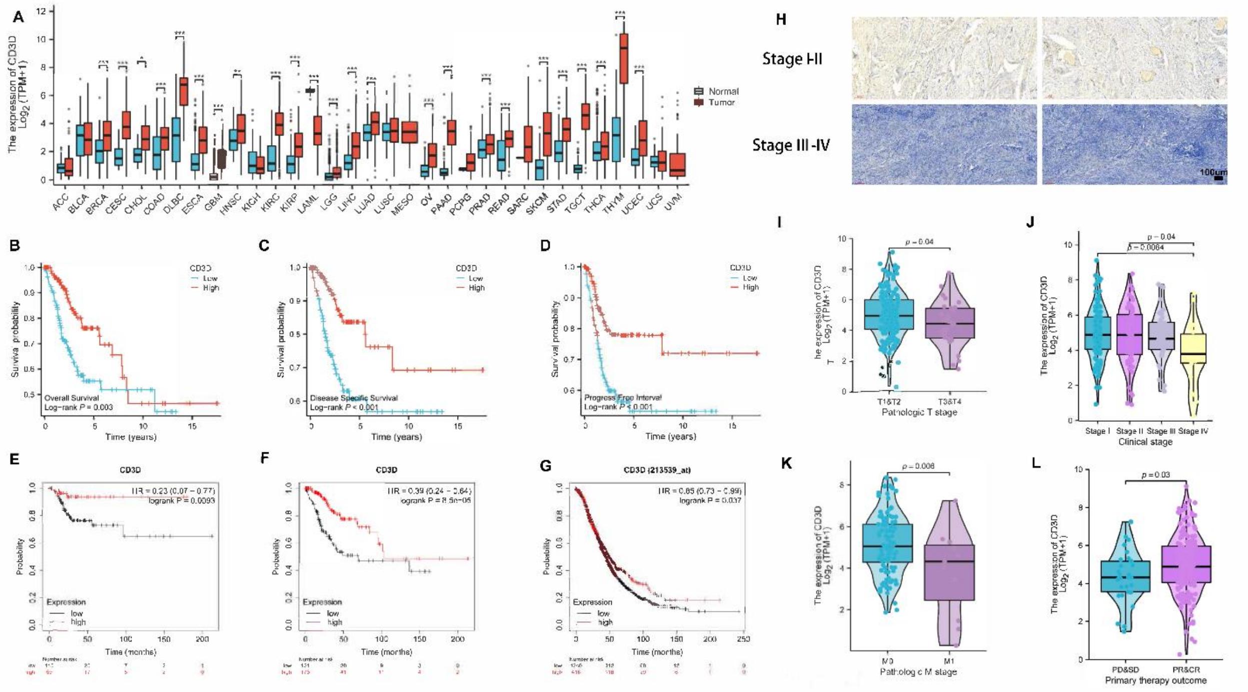

Fig. S4: Bioinformatic analyses revealed CD3D emerged as a protective factor in CC patients. **(A)** Pan-cancer analysis of CD3D expression in tumorous and normal samples were displayed (p-Value: *** p < 0.001, ** p < 0.01, * p < 0.05, ns p > 0.05). **(B-D)** The prognostic values of CD3D expression in CC patients. Patients were marked with high expression or low expression depending on comparing with the median expression level, Kaplan-Meier (KM) survival analysis portrayed the effects of CD3D expression on the overall survival (B), disease-free survival (C) and progress free interval (D) in TCGA-CESC cohort, p-Value was calculated by log-rank test. **(E-G)** The correlation of CD3D expression with overall survival using the Kaplan-Meier plotter. **(H)** The expression of CD3D protein in I-II stage and III-IV stage CC tissues was detected by IHC (magnification ×100). **(I-K)** Correlation analysis of CD3D expression with clinicopathological characteristics of CC patients. Pathologic T stage classification (I), Clinical stage classification (J) and Pathologic M stage classification (K) was displayed respectively, by Kruskal-Wallis rank sum

test. **(L)** The correlation of CD3D expression with primary therapy outcomes of CC patients, Wilcoxon rank sum was applied for the significance test.

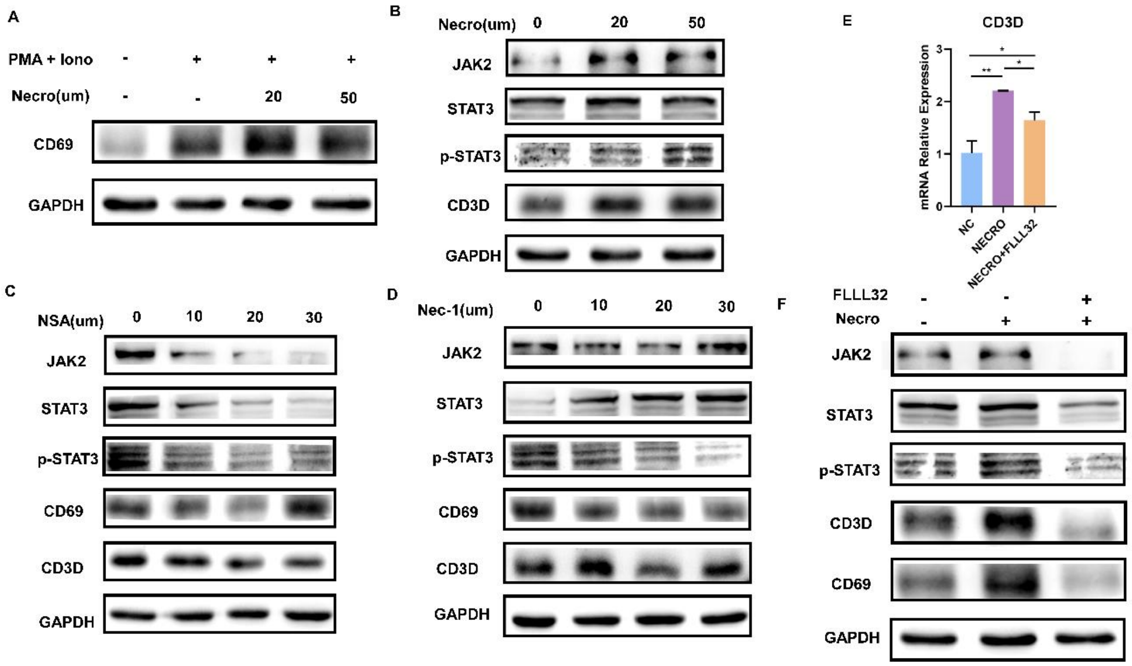

Fig. S5: Necroptosis up-regulates CD3D via JAK2-STAT3 signaling pathway in activated T cells. (A) The protein level of CD69. **(B)** The protein level of JAK2, STAT3, p-STAT3 and CD3D in activated T cells was much stronger with the induction of necroptosis. **(C, D)** The protein level of JAK2, STAT3, p-STAT3, CD3D and CD69 exhibits to decrease in the condition of NSA (C) or Nec-1 (D). **(E)** RT-qPCR results revealed CD3D expression of activated T cells was higher in the treatment of necroptosis inducer and this effect was inhibited by FLLL32 (p-Value: ** $p < 0.01$, * $p < 0.05$, ns $p > 0.05$). **(F)** The protein level of JAK2, STAT3, p-STAT3, CD3D and CD69 exhibits to decrease in the condition of necroptosis and this effect was inhibited by FLLL32.

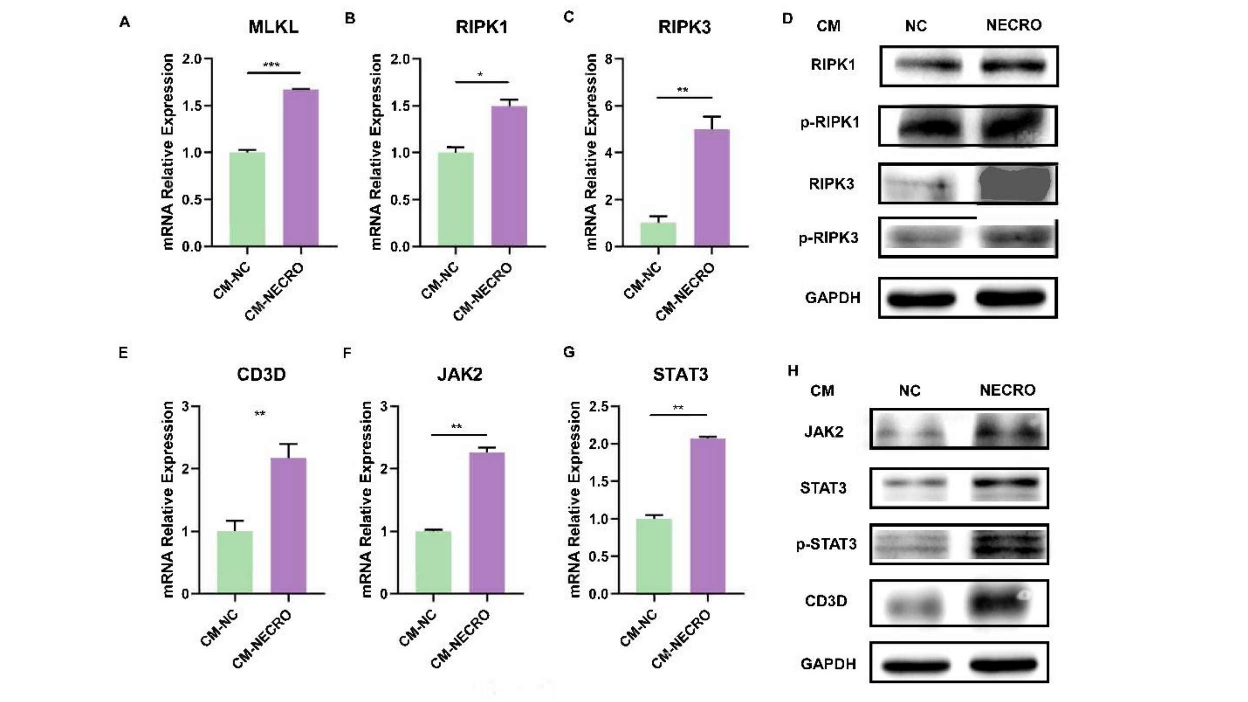

Fig. S6: CC cells undergoing necroptosis activated the necroptosis signals and the JAK2-STAT3 signaling pathway of Jurkat cells. (A-C) RT-qPCR results showed the expressing amount of MLKL (A), RIPK1 (B) and RIPK3 (C) was up-regulated in the treated group (p-Value: *** $p < 0.001$, ** $p < 0.01$, * $p < 0.05$, ns $p > 0.05$). **(D)** The protein level of RIPK1, p-RIPK1, RIPK3 and p-RIPK3 is much stronger in the treated group. **(E-G)** RT-qPCR results showed the expression level of CD3D (E), JAK2 (F) and STAT3 (G) got elevated in the treated group, with p-value <0.01(**). **(H)** The protein level of JAK2, STAT3, p-STAT3 and CD3D exhibited to increase in the treated group. Notes: "CM-NC" means Jurkat cells cocultured with the supernatant of SiHa CC cells (without any treatment), "CM-NECRO" means Jurkat cells cocultured with the supernatant of SiHa CC cells (pretreated with TNFα + Z-VAD-FMK at a concentration of 20 μm the day before).

Table S1. Primer nucleotide sequence of this study.

Gene	Primer nucleotide sequence
GAPDH	Forward: 5'-CTGGGCTACACTGAGCACC-3'
	Reverse: 5'-AAGTGGTCGTTGAGGGCAATG-3'
CD3D	Forward: 5'- ACTGGCTACCCTTCTCTCG-3'
	Reverse: 5'- CCGTTCCCTCTACCCATGTGA-3'
CXCL9	Forward: 5'-TGAGAAAGGGTCGCTGTTCC-3'
	Reverse: 5'-GGGCTTGGGGCAAATTGTTT-3'
CXCL10	Forward: 5'- GTGGCATTCAAGGAGTACCTC-3'
	Reverse: 5'- TGATGGCCTTCGATTCTGGATT-3'
CXCL11	Forward: 5'- GACGCTGTCTTTGCATAGGC-3'
	Reverse: 5'- GGATTTAGGCATCGTTGTCCTTT-3'
CXCL13	Forward: 5'-GCTTGAGGTGTAGATGTGTCC-3'
	Reverse: 5'-CCCACGGGGCAAGATTTGAA-3'
LAG3	Forward: 5'- GCGGGGACTTCTCGCTATG-3'
	Reverse: 5'- GGCTCTGAGAGATCCTGGGG-3'
TIGIT	Forward: 5'- TCTGCATCTATCACACCTACCC-3'
	Reverse: 5'- CCACCACGATGACTGCTGT-3'
TIM3	Forward: 5'- CTGCTGCTGCTGCTGCTACTAC-3'
	Reverse: 5'- TGAGCACCACGTTGCCACATTC-3'

Gene	Primer nucleotide sequence
CTLA4	Forward: 5’- GGAGGAAGTCAGAATCTGGGCA-3’
	Reverse: 5’- ACGGGACTCTACATCTGCAAGG-3’
VISTA	Forward: 5’- ACGCCGTATTCCCTGTATGTC-3’
	Reverse: 5’- TTGTAGAAGGTCACATCGTGC-3’
PD-1	Forward: 5’- CCAGGATGGTTCTTAGACTCCC-3’
	Reverse: 5’- TTTAGCACGAAGCTCTCCGAT-3’
CCR4	Forward: 5’- CCCACGGATATAGCAGACACC-3’
	Reverse: 5’- GTGCAAGGCTTGGGGATACT-3’
CCR5	Forward: 5’- TTCTGGGCTCCCTACAACATT-3’
	Reverse: 5’- TTGGTCCAACCTGTTAGAGCTA-3’
CCR7	Forward: 5’-TGAGGTCACGGACGATTACAT-3’
	Reverse: 5’-GTAGGCCCACGAAACAAATGAT-3’
CCR9	Forward: 5’- ATGTCAGGCAGTTTGCGAG-3’
	Reverse: 5’- TGCAGTACCAGTAGACAAGGAT-3’
IL2	Forward: 5’- AACTCCTGTCTTGCATTGCAC-3’
	Reverse: 5’- GCTCCAGTTGTAGCTGTGTTT-3’
IL6	Forward: 5’- CCTGAACCTTCCAAAGATGGC-3’
	Reverse: 5’- TTCACCAGGCAAGTCTCCTCA-3’
IL13	Forward: 5’-GAAGGCTCCGCTCTGCAAT-3’

Gene	Primer nucleotide sequence
	Reverse: 5'-TCCAGGGCTGCACAGTACA-3'
CD25	Forward: 5'- CGCAGAATAAAAAGCGGGTCA-3'
	Reverse: 5'- ACTTGTTTCGTTGTGTTCCGA-3'
CD69	Forward: 5'- ATTGTCCAGGCCAATACACATT-3'
	Reverse: 5'- CCTCTCTACCTGCGTATCGTTTT-3'
GZMB	Forward: 5'-CCCTGGGAAAACACTCACACA-3'
	Reverse: 5'-GCACAACTCAATGGTACTGTCG-3'
IFNγ	Forward: 5'-TCGGTAACTGACTTGAATGTCCA-3'
	Reverse: 5'-TCGCTTCCCTGTTTTAGCTGC-3'
MLKL	Forward: 5'- AGGAGGCTAATGGGGAGATAGA-3'
	Reverse: 5'- TGGCTTGCTGTTAGAAACCTG-3'
RIPK1	Forward: 5'-GGGAAGGTGTCTCTGTGTTTC-3'
	Reverse: 5'-CCTCGTTGTGCTCAATGCAG-3'
RIPK3	Forward: 5'-ATGTCGTGCGTCAAGTTATGG-3'
	Reverse: 5'-CGTAGCCCCACTTCCTATGTTG-3'
VEGFA	Forward: 5'-AGGGCAGAATCATCACGAAGT-3'
	Reverse: 5'-AGGGTCTCGATTGGATGGCA-3'

Table S2. The information of primary antibodies.

**Antibody**	**Company**	**Dilution**	**Host**	**Catalog**	**kDa**

GAPDH	Proteintech	1:10000	HRP	HRP-60004	36
β-Actin	Abclonal	1:10000	Rabbit	AC026	43
CD3D	Abclonal	1:1000	Rabbit	A9770	23
JAK2	Abmart	1:1000	Rabbit	T55287	130
STAT3	HuaBio	1:1000	Rabbit	ET1607-38	88
p-STAT3(Tyr705)	Santa Cruz	1:500	Mouse	sc-8059	88
CD69	Proteintech	1:1000	Rabbit	10803-1-AP	28/32
CD8	Proteintech	1:1000	Rabbit	66868-1-Ig	32
RIPK1	Abclonal	1:1000	Rabbit	A19580	75
p-RIPK1(S166)	Abclonal	1:1000	Rabbit	AP1230	75
RIPK3	Abclonal	1:1000	Rabbit	A5431	57
p-RIPK3(T231/S232)	Abclonal	1:1000	Rabbit	AP1260	53
VEGFA	Abclonal	1:1000	Rabbit	A12303	27

Table S3. The results of differentially expressed genes (DEGs) between high necroptosis group and low necroptosis group.

Gene	conMean	treatMean	logFC	pValue	FDR
LAG3	2.50099	7.81689	1.64409	0.00000	0.00000
C16orf89	1.94678	0.41451	-2.23162	0.00689	0.02163
IL21R	0.48308	1.48450	1.61965	0.00000	0.00000
ATRNL1	0.16769	0.04224	-1.98899	0.00106	0.00502
SCML4	0.12397	0.36322	1.55089	0.00000	0.00000
CXorf65	0.22703	0.45537	1.00416	0.00000	0.00001
SMTNL1	0.27559	0.66979	1.28118	0.00000	0.00000

Gene	conMean	treatMean	logFC	pValue	FDR
CFAP43	0.41805	0.16663	-1.32699	0.04936	0.09997
SASH3	3.55372	8.33462	1.22979	0.00000	0.00000
CCDC173	0.58133	0.21991	-1.40242	0.00007	0.00055
ARHGDIG	0.16488	0.07154	-1.20459	0.00064	0.00336
MS4A1	0.38423	0.95418	1.31228	0.00030	0.00184
SOSTDC1	3.99059	0.95411	-2.06438	0.00000	0.00000
DMRT2	1.83079	0.75859	-1.27108	0.00009	0.00072
TTC25	1.15406	0.39064	-1.56281	0.00002	0.00017
OAS2	14.36995	33.09746	1.20367	0.00000	0.00000
CD3D	6.81857	18.33032	1.42669	0.00000	0.00000
CNTN1	3.67918	1.20050	-1.61575	0.00000	0.00004
SLC45A1	0.46366	0.23071	-1.00700	0.00000	0.00002
CTLA4	1.21810	2.82003	1.21108	0.00000	0.00000
OPRK1	0.38177	0.02054	-4.21634	0.00000	0.00003
MAGEB17	0.23581	0.02724	-3.11384	0.00025	0.00160
WFIKKN2	0.35968	0.01945	-4.20864	0.00008	0.00064
TMPRSS15	0.20165	0.00463	-5.44326	0.00095	0.00459
HRC	0.23348	0.10678	-1.12866	0.00001	0.00007
BPIFC	0.19599	0.05832	-1.74875	0.00945	0.02773
BEND5	4.50353	0.45947	-3.29300	0.00000	0.00000
CYTH4	1.53989	3.20507	1.05753	0.00000	0.00000
ADGRE1	0.15300	0.49169	1.68418	0.00000	0.00000
NCR1	0.13231	0.37460	1.50144	0.00000	0.00000

Gene	conMean	treatMean	logFC	pValue	FDR
NKAIN2	0.32073	0.13964	-1.19965	0.01396	0.03738
AK5	0.27885	0.07971	-1.80672	0.00077	0.00388
DLGAP1	0.32497	0.04516	-2.84725	0.00008	0.00066
GZMM	1.56109	3.67156	1.23384	0.00000	0.00000
FOXA2	3.33856	1.08812	-1.61739	0.00161	0.00697
SPSB4	0.58437	0.24583	-1.24920	0.00152	0.00670
SV2A	2.90093	0.94047	-1.62506	0.00293	0.01112
C1QB	51.60136	133.29852	1.36918	0.00000	0.00000
SNCG	14.24272	35.21421	1.30593	0.00094	0.00455
NRXN3	0.18148	0.04664	-1.96030	0.00000	0.00005
OBP2A	0.20436	0.04571	-2.16042	0.00777	0.02379
ANXA10	0.48771	1.22367	1.32711	0.00233	0.00929
LRFN2	0.16274	0.04161	-1.96757	0.03819	0.08248
SMTNL2	1.04907	0.15828	-2.72858	0.00007	0.00054
GPC3	16.05802	5.16964	-1.63516	0.00000	0.00001
GPR171	0.86685	2.41208	1.47643	0.00000	0.00000
TNFRSF13B	0.13137	0.29676	1.17569	0.00010	0.00075
RAB6B	2.00059	0.77319	-1.37153	0.00000	0.00002
AADAT	2.63585	0.91705	-1.52319	0.00000	0.00000
C2orf40	0.45568	0.22312	-1.03022	0.00005	0.00042
CD163	2.91851	6.12424	1.06930	0.00000	0.00000
ABCA4	0.71155	0.26525	-1.42362	0.00053	0.00288
TMEM52B	0.39902	1.07208	1.42588	0.00000	0.00000

Gene	conMean	treatMean	logFC	pValue	FDR
EPSTI1	5.99573	15.11298	1.33378	0.00000	0.00000
KLRC2	0.21872	0.61105	1.48219	0.00000	0.00000
DEGS2	6.49774	3.20536	-1.01945	0.00000	0.00001
IFNG	0.42310	1.93961	2.19670	0.00000	0.00000
LEF1	3.83545	1.63380	-1.23117	0.00540	0.01784
SYN2	0.52442	0.10675	-2.29650	0.00178	0.00754
CYP26A1	1.67166	0.21979	-2.92707	0.00000	0.00000
ESRRG	0.21783	0.09370	-1.21713	0.00000	0.00002
CFAP73	2.12858	0.94139	-1.17702	0.00065	0.00339
CLDN11	1.02638	0.47788	-1.10285	0.00169	0.00724
NKX2-1	1.14006	0.10346	-3.46196	0.01517	0.03988
TNFRSF9	0.39623	0.96944	1.29080	0.00000	0.00000
IL2RA	0.95987	2.19748	1.19494	0.00000	0.00000
MAPK8IP1	4.70555	2.09037	-1.17061	0.00000	0.00000
FAM43B	0.27891	0.12612	-1.14497	0.02703	0.06307
SLAMF8	2.96892	6.81261	1.19827	0.00000	0.00000
NXPH3	0.72657	0.30250	-1.26418	0.00483	0.01634
MYO1G	0.91621	1.87031	1.02953	0.00000	0.00000
IL2RB	3.12915	8.32792	1.41219	0.00000	0.00000
IKZF1	0.84451	1.74015	1.04302	0.00000	0.00000
KLRC4-KLRK1	0.05441	0.15060	1.46892	0.00000	0.00000
MYOZ1	0.40665	0.17979	-1.17749	0.00000	0.00002
JAML	1.03042	2.07889	1.01258	0.00000	0.00001

Gene	conMean	treatMean	logFC	pValue	FDR
RASL10B	0.84864	0.36297	-1.22531	0.00002	0.00023
PYHIN1	0.33108	0.98084	1.56682	0.00000	0.00000
FATE1	0.24694	0.57598	1.22187	0.02995	0.06833
C6orf15	23.49413	1.13572	-4.37063	0.00323	0.01199
VSIG8	5.46861	0.37105	-3.88151	0.00003	0.00029
CCDC74B	1.14395	0.52490	-1.12390	0.00000	0.00006
GFRA3	2.95831	0.48636	-2.60468	0.00000	0.00000
JAKMIP1	0.28196	0.70595	1.32408	0.00000	0.00000
BAMBI	12.02651	3.83313	-1.64962	0.00000	0.00002
CKB	76.75024	36.43098	-1.07501	0.00000	0.00000
ADAMTSL4	3.10589	7.45979	1.26413	0.00000	0.00001
CD244	0.33107	1.02932	1.63648	0.00000	0.00000
IFNK	0.11380	0.89828	2.98065	0.00000	0.00000
ASTL	0.24162	0.07880	-1.61649	0.00366	0.01314
ACBD7	1.48224	0.73069	-1.02044	0.02072	0.05126
HLA-DOA	5.31327	11.77549	1.14811	0.00000	0.00000
KRT3	1.13260	0.37373	-1.59958	0.04348	0.09094
P2RY6	1.50218	3.30842	1.13909	0.00000	0.00000
FUT7	0.33012	0.68170	1.04616	0.00000	0.00000
PCDHB4	0.36838	0.18068	-1.02771	0.00108	0.00511
MYL3	0.16322	0.06525	-1.32291	0.00073	0.00372
EPHA3	0.89133	0.22884	-1.96164	0.00027	0.00168
TRPM5	0.27685	0.09997	-1.46962	0.00002	0.00017

Gene	conMean	treatMean	logFC	pValue	FDR
PRPH	0.64842	0.07262	-3.15841	0.00000	0.00000
POMC	4.62126	1.68135	-1.45866	0.02044	0.05068
ITK	0.45522	1.00297	1.13966	0.00000	0.00000
ALKAL1	0.17369	0.03809	-2.18903	0.00180	0.00760
LTA	0.32998	0.81159	1.29836	0.00000	0.00000
SYT8	6.57878	14.11334	1.10117	0.00013	0.00096
APOL1	106.7296	241.80957	1.17991	0.00000	0.00000
FBN3	0.49690	0.22953	-1.11428	0.00005	0.00045
AQP2	0.20325	0.06810	-1.57747	0.00067	0.00351
KRTAP3-1	1.01226	0.07015	-3.85104	0.00023	0.00148
MAGEA10	1.31424	0.65685	-1.00059	0.00681	0.02143
SH2D1A	0.68726	1.93962	1.49685	0.00000	0.00000
ADH1C	15.97489	2.84227	-2.49069	0.00005	0.00046
LRRIQ1	0.42736	0.12994	-1.71761	0.01309	0.03548
COX4I2	3.09597	0.91957	-1.75135	0.03396	0.07542
GPC4	12.62550	4.68859	-1.42912	0.00001	0.00008
REEP2	1.78477	0.57615	-1.63121	0.00000	0.00001
SYNDIG1	0.46025	0.20868	-1.14109	0.00001	0.00012
TVP23A	0.24634	0.51112	1.05300	0.00100	0.00478
ETV1	1.40879	0.50778	-1.47217	0.00014	0.00097
NXPE4	0.01118	0.32881	4.87778	0.00128	0.00583
SLC27A6	0.22049	0.06060	-1.86345	0.00008	0.00062
CHST9	0.83567	0.15170	-2.46173	0.00036	0.00212

Gene	conMean	treatMean	logFC	pValue	FDR
CSDC2	0.71662	0.32311	-1.14918	0.00003	0.00032
CD52	14.74173	30.82307	1.06410	0.00000	0.00000
CLIC6	19.32668	7.51460	-1.36283	0.00479	0.01623
NME9	0.24295	0.12118	-1.00351	0.01174	0.03266
HSD17B2	0.57435	1.22733	1.09553	0.00026	0.00166
IFI44L	4.23496	10.37921	1.29328	0.00000	0.00000
S100A5	0.88259	0.34380	-1.36016	0.00000	0.00000
KL	0.33345	0.16350	-1.02816	0.03832	0.08267
DLX1	0.66307	0.11481	-2.52996	0.00706	0.02209
TNFSF14	0.40573	0.91750	1.17720	0.00000	0.00000
CCDC187	0.61411	0.29066	-1.07916	0.00145	0.00646
MFAP4	17.31536	7.84170	-1.14281	0.00103	0.00490
FCGR1A	0.81341	1.84498	1.18155	0.00000	0.00000
LILRB5	0.17615	0.37023	1.07164	0.00000	0.00005
IGSF11	0.26552	0.08037	-1.72404	0.00000	0.00000
ST8SIA2	0.32876	0.10672	-1.62325	0.00338	0.01241
FILIP1	0.27805	0.11075	-1.32804	0.00009	0.00069
CFAP47	0.14219	0.06930	-1.03683	0.00881	0.02621
SH2D1B	0.26510	0.54926	1.05097	0.00000	0.00000
DDIT4L	1.02262	0.46234	-1.14524	0.00317	0.01183
CLEC9A	0.07554	0.17252	1.19139	0.00004	0.00032
VCAN	6.34328	2.97640	-1.09166	0.00037	0.00215
STK32B	0.52881	0.15195	-1.79912	0.00095	0.00461
KLRC1	0.291194	0.712573	1.291058	3.85E-14	3.95E-12

Gene	conMean	treatMean	logFC	pValue	FDR
NEXMIF	0.452587	0.188198	-1.26594	3.67E-08	8.78E-07
TMIGD2	0.5373	1.489874	1.47139	3.18E-15	4.47E-13
TCF23	0.176079	0.016369	-3.42715	0.006892	0.021629
TESPA1	0.293613	0.638124	1.119923	5.33E-12	3.15E-10
CASQ2	0.162371	0.079769	-1.0254	4.01E-06	5.25E-05
C4A	0.525394	1.124708	1.098079	0.001241	0.005695
TMEM252	0.333485	0.058177	-2.51911	0.006872	0.021584
CD2	6.191237	17.24026	1.477482	1.25E-14	1.54E-12
ZAP70	1.317171	3.093177	1.231647	8.53E-13	5.91E-11
ENDOU	1.321408	0.439645	-1.58767	0.023977	0.057447
KCNA3	0.153014	0.328266	1.101199	1.65E-06	2.44E-05
C10orf67	0.193224	0.393201	1.024992	4.93E-06	6.23E-05
KCNH8	0.173323	0.050186	-1.78812	5.63E-06	6.98E-05
APOL3	4.316227	12.12766	1.490459	3.07E-23	1.05E-19
LAIR2	0.364092	1.061271	1.543417	5.91E-08	1.34E-06
GAP43	0.222244	0.100295	-1.14789	0.004015	0.014179
SLC30A3	0.382477	0.166158	-1.20282	0.048916	0.099304
NCAM1	0.342681	0.16313	-1.07084	0.002921	0.011095
KLRC4	0.08484	0.236167	1.476996	1.04E-10	4.57E-09
ARG1	0.235279	0.097513	-1.27071	3.10E-05	0.000291
AGT	2.888156	0.83468	-1.79085	0.004139	0.01452
IDO1	15.36955	65.85267	2.099167	3.15E-15	4.47E-13
SULT1C4	0.539777	0.219229	-1.29993	0.000773	0.0039
OXGR1	1.057735	0.284273	-1.89563	8.93E-08	1.93E-06
CD48	2.150199	5.636849	1.390418	6.01E-13	4.39E-11

Gene	conMean	treatMean	logFC	pValue	FDR
SAMD3	0.109118	0.257032	1.236066	1.47E-13	1.26E-11
ULBP1	1.543568	0.700123	-1.14059	7.04E-05	0.000575
ACSBG1	0.159938	0.065035	-1.29823	0.002909	0.011073
CAPS	25.61021	6.899282	-1.8922	1.21E-05	0.000132
HLA-DQA2	11.60736	34.17237	1.557791	1.64E-06	2.44E-05
SLC7A3	0.259845	0.080461	-1.6913	0.000121	0.000885
IFNL1	0.097769	0.26007	1.41145	1.34E-10	5.80E-09
KLHDC7B	18.89172	65.85943	1.801636	1.96E-17	5.34E-15
KIAA1549	1.057811	0.447648	-1.24065	3.12E-07	5.68E-06
EPHA7	0.516983	0.076705	-2.75273	0.000299	0.00184
PDZK1IP1	71.02724	145.8316	1.037859	9.62E-09	2.65E-07
PARP15	0.310407	0.669886	1.109755	9.83E-10	3.41E-08
C1QA	61.7985	150.94	1.288332	1.06E-09	3.64E-08
TNF	1.551587	3.911606	1.334016	1.12E-14	1.39E-12
SIGLEC14	0.523524	1.207391	1.205566	5.09E-09	1.51E-07
GPR78	0.29341	0.60682	1.048353	0.001176	0.005438
LDLRAD1	1.227079	0.496218	-1.30618	3.54E-05	0.000325
ITGBL1	0.341521	0.141498	-1.27119	0.000516	0.002814
INSL4	0.220989	0.88445	2.000808	0.036516	0.079907
KLRD1	0.129511	0.464021	1.841119	1.43E-15	2.24E-13
SPIB	0.326244	2.510961	2.944216	1.71E-05	0.000176
TAGAP	1.040757	2.135462	1.036914	4.10E-11	1.95E-09
SERTM2	0.360396	0.07731	-2.22086	0.000704	0.003633
GRAP2	0.319353	0.791188	1.308868	7.55E-13	5.34E-11
STAT1	47.81626	98.8924	1.048358	2.30E-18	8.12E-16

Gene	conMean	treatMean	logFC	pValue	FDR
GALNT16	0.594379	0.177681	-1.7421	3.61E-05	0.000329
SBK1	6.148892	2.22185	-1.46856	7.41E-13	5.26E-11
HLA-DPA1	27.56608	70.83473	1.361563	1.99E-14	2.21E-12
MAPK15	5.802018	2.678982	-1.11487	0.044772	0.092979
PRR15	13.30752	5.527864	-1.26745	4.46E-05	0.000391
FASLG	0.553596	2.1182	1.935935	4.32E-20	2.95E-17
LINGO1	1.119575	0.51277	-1.12657	0.00104	0.004952
TUBA1A	33.54401	16.32773	-1.03873	7.60E-06	8.93E-05
IGF1	0.785848	0.240761	-1.70665	0.000323	0.001954
CD74	483.9716	1157.923	1.258545	1.23E-15	2.02E-13
ADAMTS18	0.44568	0.052248	-3.09255	8.82E-05	0.000684
MSX2	5.692943	1.425407	-1.9978	0.005231	0.017421
SP140	0.514536	1.362914	1.40535	1.80E-12	1.17E-10
RPRML	0.388061	0.160408	-1.27454	3.53E-05	0.000324
CD7	3.67307	10.275	1.48408	5.62E-20	3.49E-17
NCMAP	1.337735	0.564064	-1.24586	0.007534	0.023246
ADAMTS5	0.404441	0.197751	-1.03224	0.000849	0.004195
CLEC18B	0.198417	0.048483	-2.03298	0.001652	0.007105
PDCD1	1.220431	2.94425	1.27051	1.70E-11	8.78E-10
OMD	0.367901	0.158566	-1.21423	0.010188	0.029277
HLA-DQB1	21.36404	53.54669	1.325613	3.63E-13	2.78E-11
DNAI1	0.332805	0.082648	-2.00962	0.042737	0.089785
KLHDC7A	1.979996	0.964634	-1.03744	0.004103	0.014413
NCR3	0.405774	0.924597	1.188148	7.06E-13	5.10E-11
SEMA3E	0.452805	0.090341	-2.32543	5.32E-05	0.000454

Gene	conMean	treatMean	logFC	pValue	FDR
HRH2	0.254742	0.588222	1.207323	0.003033	0.011402
PCSK2	0.29093	0.023836	-3.60944	2.46E-05	0.00024
DMRT3	1.313511	0.144534	-3.18395	1.09E-06	1.70E-05
PGR	1.261977	0.354857	-1.83037	0.01901	0.047748
IL16	0.593838	1.208804	1.025439	2.91E-10	1.15E-08
PCDH18	1.540858	0.646437	-1.25315	0.042483	0.089354
CLEC2L	0.573937	0.223792	-1.35873	2.91E-06	3.98E-05
SPOCK1	3.57564	1.606254	-1.1545	1.38E-07	2.80E-06
ZNF135	0.234571	0.097015	-1.27374	0.004293	0.014924
AKR1C1	15.74168	3.714967	-2.08317	0.011277	0.031704
SIGLEC12	0.365677	0.81207	1.151033	1.06E-05	0.000119
MAP3K19	0.250496	0.02822	-3.15001	0.046752	0.095968
OLR1	3.135224	9.469644	1.594741	3.04E-07	5.53E-06
SNAI3	0.748458	2.044073	1.449453	8.98E-05	0.000694
SCGB1A1	19.08002	4.541573	-2.0708	4.04E-05	0.000362
VSTM1	0.051454	0.187926	1.868796	0.001051	0.004996
ARX	1.124785	0.046781	-4.58757	0.004143	0.014531
PDK4	3.047706	1.360295	-1.1638	6.35E-05	0.000527
MAP1A	0.888207	0.433388	-1.03524	2.59E-09	8.21E-08
KLK14	7.304057	1.837146	-1.99123	0.029454	0.067462
DACH1	1.641907	0.713848	-1.20168	8.63E-06	9.96E-05
ALKAL2	1.493541	0.342248	-2.12562	0.005107	0.0171
SLC29A4	1.503658	0.447315	-1.74911	1.45E-06	2.18E-05
CCDC114	2.575321	0.78633	-1.71155	6.35E-05	0.000527
FOXJ1	26.40253	11.71082	-1.17283	0.011656	0.032471

Gene	conMean	treatMean	logFC	pValue	FDR
GBP5	3.683643	16.12466	2.130064	2.51E-14	2.67E-12
SLA	1.465619	3.10619	1.083636	1.31E-09	4.43E-08
SLA2	0.946598	2.855351	1.592844	1.22E-18	4.76E-16
MDH1B	0.631176	0.304469	-1.05174	1.57E-05	0.000164
NKD2	4.418931	2.169396	-1.0264	0.028504	0.06585
SLAMF1	0.436317	0.959146	1.136374	6.49E-10	2.35E-08
THBS4	1.128887	0.506325	-1.15676	1.95E-05	0.000197
NLRC3	0.646649	1.392943	1.107081	3.38E-11	1.65E-09
BMPR1B	2.189571	0.827115	-1.40449	0.000246	0.001587
KHDRBS3	1.214542	0.563394	-1.1082	6.23E-07	1.05E-05
ARHGAP9	1.634051	3.948258	1.272764	1.60E-17	4.56E-15
SERPING1	36.79998	78.9407	1.101064	7.41E-13	5.26E-11
CMKLR1	1.007854	2.02127	1.003976	2.49E-07	4.68E-06
HUNK	1.764301	0.849108	-1.05508	3.59E-05	0.000328
CFAP100	0.685834	0.07702	-3.15456	0.008133	0.024664
CD160	0.12542	0.277285	1.144595	5.27E-06	6.59E-05
CCL5	25.08329	86.53985	1.786638	1.43E-18	5.44E-16
SLC25A21	0.25581	0.119425	-1.09897	1.65E-07	3.27E-06
C9orf24	3.22224	0.682982	-2.23815	4.09E-06	5.33E-05
GLDC	1.337096	5.407539	2.015869	5.24E-09	1.54E-07
FGFBP2	1.368896	0.170754	-3.00302	0.031612	0.071378
PDCD1LG2	1.07053	2.645305	1.305109	1.42E-12	9.33E-11
ACTG2	10.84945	1.956106	-2.47157	0.000182	0.001239
RBP2	0.3678	0.128226	-1.52023	0.000453	0.002531
GRM8	0.499402	0.161856	-1.62549	0.026462	0.062111

Gene	conMean	treatMean	logFC	pValue	FDR
ZSCAN18	1.152575	0.556082	-1.05149	0.012912	0.035079
HLA-DQA1	10.52964	31.15788	1.565141	8.69E-12	4.86E-10
MEDAG	2.212705	1.009242	-1.13254	0.024631	0.05861
SAMD9L	3.082087	7.462756	1.275801	4.94E-19	2.11E-16
C2orf70	0.7768	0.345333	-1.16956	0.004019	0.01418
CCDC151	0.678663	0.275606	-1.30009	0.000427	0.00242
PPP1R9A	0.763039	0.35692	-1.09616	0.036201	0.079321
CST7	5.551558	13.20141	1.249727	4.55E-11	2.14E-09
TF	1.932484	0.811599	-1.25162	0.019075	0.047867
GZMK	1.388428	4.419574	1.670455	2.48E-08	6.20E-07
TNFSF13B	1.699474	3.877515	1.190044	1.02E-10	4.51E-09
PRKD1	1.145224	0.417107	-1.45714	1.54E-05	0.000162
LRRC10B	1.887626	0.563652	-1.7437	1.75E-05	0.000179
ADAM8	9.046864	18.5407	1.035206	6.24E-17	1.42E-14
CDHR3	0.482859	0.220299	-1.13214	0.001567	0.006836
SERPINI2	0.286035	0.04376	-2.70851	0.000981	0.004725
PCDHB2	0.911213	0.321336	-1.50371	0.026419	0.062019
IL10RA	1.873556	4.064779	1.117398	1.38E-10	5.89E-09
TMEM74	0.234232	0.092582	-1.33913	0.000157	0.001094
SMOC2	5.176566	1.950912	-1.40785	3.63E-06	4.83E-05
ADAMDEC1	2.010918	5.103835	1.343728	1.89E-07	3.65E-06
EFHD1	7.764386	2.750917	-1.49696	0.00046	0.002561
TMEM156	0.396296	0.832036	1.070068	8.38E-12	4.73E-10
SFRP4	32.81384	11.52787	-1.50918	0.000224	0.001462
CNN1	8.845747	2.308077	-1.93829	1.83E-06	2.67E-05

Gene	conMean	treatMean	logFC	pValue	FDR
TFAP2B	0.268584	0.046281	-2.53687	1.60E-05	0.000167
TAS1R1	0.197624	0.0872	-1.18036	0.049321	0.099973
LRRTM1	0.736458	0.028698	-4.68159	0.004232	0.014769
SLC38A8	0.764545	0.151819	-2.33225	0.000102	0.000772
CSRNP3	0.275107	0.13382	-1.03969	0.000332	0.001995
SAA2-SAA4	1.258326	3.746332	1.573973	3.03E-10	1.18E-08
AADACL2	0.190792	0.029718	-2.68258	0.001195	0.005517
CRTAC1	0.733282	0.305093	-1.26512	0.009697	0.028233
WARS	34.05128	109.0059	1.678626	1.17E-15	1.94E-13
ITGAL	1.830052	4.807364	1.393361	3.33E-13	2.57E-11
AXIN2	3.784795	0.81587	-2.2138	0.00018	0.001223
RSPH14	0.243712	0.115971	-1.07141	1.90E-05	0.000193
CRHR1	0.337915	0.134161	-1.3327	3.72E-07	6.63E-06
CD84	0.646072	1.357444	1.071125	2.01E-08	5.16E-07
SIGLEC10	1.273368	2.909559	1.192151	6.47E-08	1.45E-06
SNX20	0.838698	1.9173	1.192853	1.39E-12	9.25E-11
CHI3L2	1.058394	2.276826	1.105147	3.03E-06	4.11E-05
TMEM35A	1.007248	0.17171	-2.55237	1.62E-10	6.74E-09
LMOD1	3.669345	1.582243	-1.21355	1.07E-05	0.00012
LRP4	3.008552	1.371981	-1.13281	1.43E-05	0.000152
CDHR4	0.456065	0.151673	-1.58828	0.005252	0.017469
SOBP	1.121221	0.360676	-1.63629	3.38E-09	1.04E-07
TYRP1	0.201852	0.084842	-1.25045	0.001947	0.008099
RXRG	0.334663	0.02892	-3.53259	0.000517	0.002815
IL15RA	4.202949	9.265822	1.140517	1.93E-28	2.64E-24

Gene	conMean	treatMean	logFC	pValue	FDR
ARMC3	0.873616	0.228876	-1.93243	0.004567	0.015674
C1GALT1C1L	0.921076	0.406646	-1.17955	0.000187	0.001267
LRRIQ4	0.489847	0.152099	-1.68732	0.009875	0.0286
PPM1L	2.345795	0.91362	-1.36041	1.47E-14	1.75E-12
CXCL13	8.958438	26.19142	1.547775	4.34E-08	1.02E-06
CTXN1	11.23595	5.013862	-1.16413	1.50E-06	2.25E-05
PKNOX2	0.262011	0.099948	-1.39038	1.82E-05	0.000186
STMN2	1.539176	0.411879	-1.90186	2.94E-05	0.000278
SIRPG	0.895973	2.944777	1.716632	1.61E-16	3.38E-14
CCR2	0.385621	0.921332	1.256535	3.00E-07	5.47E-06
PTPRD	0.365204	0.146131	-1.32144	0.000415	0.002364
BMX	0.390378	0.133688	-1.546	0.01055	0.030132
GALNTL6	0.269797	0.046498	-2.53664	9.40E-06	0.000107
IGLL1	0.072339	0.164204	1.182639	0.001961	0.008139
TMEM233	0.326561	0.091455	-1.83623	0.022717	0.055124
C7orf57	1.310834	0.402869	-1.7021	0.001999	0.008256
TFPI2	4.484212	10.36696	1.209066	0.005231	0.017421
MYOCD	0.152812	0.052629	-1.53782	0.005336	0.017681
FABP7	0.149182	0.061065	-1.28867	0.000797	0.003986
ISM2	0.43316	0.178163	-1.2817	0.000863	0.004254
EPYC	1.530778	0.453254	-1.75587	0.004805	0.016284
SAMSN1	1.801699	3.636561	1.013217	1.17E-11	6.30E-10
PAX9	5.71052	2.262476	-1.33572	0.00035	0.00208
ISLR2	0.349613	0.157083	-1.15423	0.000218	0.001436
FAM163B	0.114186	0.535428	2.229312	1.29E-05	0.000139

Gene	conMean	treatMean	logFC	pValue	FDR
IL32	24.3986	61.90925	1.343357	6.73E-20	3.99E-17
CCDC33	0.240616	0.111247	-1.11296	0.001179	0.005451
GNAZ	1.911045	0.94304	-1.01897	3.91E-07	6.88E-06
TUBB2B	5.832745	1.336656	-2.12555	0.00156	0.006824
EVI2B	3.118456	6.875248	1.14058	9.47E-11	4.25E-09
CDH12	0.248439	0.104536	-1.2489	0.046595	0.095744
STXBP6	1.896888	0.514649	-1.88197	0.000762	0.003863
ICOS	0.578279	1.474813	1.350695	5.24E-14	4.93E-12
FCER1G	21.04614	43.04642	1.032338	3.62E-11	1.75E-09
COL21A1	2.000229	0.729499	-1.45519	4.60E-06	5.91E-05
SBSPON	1.878625	0.79144	-1.24713	3.86E-07	6.80E-06
SECTM1	12.98434	29.30958	1.1746	4.75E-15	6.29E-13
IGFBPL1	2.954419	1.039495	-1.50699	0.029096	0.066877
STOML3	0.263757	0.033097	-2.99443	8.56E-05	0.000668
PLN	1.213427	0.430259	-1.49581	7.38E-05	0.000594
C1QTNF3	3.176391	1.274137	-1.31787	0.000155	0.00108
TMEM59L	0.529385	0.174825	-1.5984	1.94E-07	3.74E-06
VWA5B2	0.448457	0.066312	-2.75762	5.88E-09	1.71E-07
ATP13A5	0.732112	0.182136	-2.00705	0.000875	0.004308
IFIT3	14.95845	44.91716	1.586306	5.85E-22	9.98E-19
SEC14L5	0.322231	0.128406	-1.32738	0.002252	0.009033
CARD17	0.893295	2.184374	1.290011	4.10E-14	4.14E-12
NDP	0.706119	0.187796	-1.91074	0.007607	0.023409
FCRL3	0.165668	0.417496	1.333464	1.05E-07	2.22E-06
TNNC2	6.015798	2.108542	-1.51251	5.82E-05	0.000489

Gene	conMean	treatMean	logFC	pValue	FDR
RUFY4	0.368162	0.791019	1.103372	7.45E-08	1.64E-06
MEFV	0.162425	0.332875	1.03521	2.51E-09	8.00E-08
SALL1	1.430097	0.500405	-1.51494	7.76E-05	0.000618
C6orf118	0.584941	0.073704	-2.98848	0.00035	0.002082
CD3E	4.403351	12.40657	1.494431	4.03E-15	5.39E-13
ADCY2	0.292691	0.06012	-2.28346	0.000203	0.00136
SPN	0.9309	2.111221	1.181379	9.74E-14	8.63E-12
LCN15	2.249849	0.315339	-2.83485	0.002676	0.010327
PRPH2	0.288115	0.143601	-1.00458	8.69E-05	0.000676
DES	16.84903	4.11018	-2.03539	0.001499	0.006613
MAP2	1.710232	0.81733	-1.0652	0.000281	0.001754
BEX1	1.396015	0.631004	-1.14559	0.003341	0.012293
SIX3	2.11167	0.261459	-3.01373	0.011222	0.031626
EFNB3	0.967908	0.34556	-1.48593	0.001197	0.005522
DOK2	2.58945	5.673647	1.131631	4.89E-14	4.67E-12
FGF7	0.699699	0.34095	-1.03717	0.00552	0.018171
CCL18	8.942867	20.19586	1.17525	2.49E-06	3.49E-05
CYBB	5.003131	10.93087	1.127506	1.13E-08	3.07E-07
STAP1	0.302922	0.610403	1.010817	7.51E-06	8.85E-05
ADAMTSL1	0.428104	0.098066	-2.12613	0.048474	0.098625
CXCL14	131.797	44.86636	-1.55461	0.003098	0.011599
TCAP	3.357747	0.483223	-2.79673	6.61E-07	1.10E-05
ADRA2B	0.894903	0.311989	-1.52023	8.51E-05	0.000666
MX1	19.16245	40.56604	1.081991	1.85E-13	1.56E-11
RND2	1.172933	0.473566	-1.30848	0.001877	0.007858

Gene	conMean	treatMean	logFC	pValue	FDR
PLCB4	2.923658	0.812614	-1.84713	3.73E-05	0.000339
ZCCHC12	0.665887	0.103308	-2.68832	1.43E-08	3.77E-07
BLK	0.103245	0.218614	1.082307	0.000467	0.00259
EFCAB12	0.252948	0.083428	-1.60023	0.001068	0.005051
CD274	2.956295	6.979429	1.239319	1.55E-12	1.01E-10
PMP2	0.177051	0.023846	-2.89236	9.35E-05	0.000718
RPE65	0.164965	0.0493	-1.74249	0.002636	0.010207
TLR8	0.258974	0.752441	1.538773	5.52E-08	1.26E-06
SLC25A48	0.35328	0.067322	-2.39166	0.000169	0.001164
TCEAL5	0.307487	0.111033	-1.46953	0.000292	0.001803
NCKAP1L	1.265453	2.616861	1.048184	3.30E-10	1.28E-08
CABCOCO1	0.337454	0.108323	-1.63936	0.000264	0.001667
MYH7B	2.013854	0.240419	-3.06634	9.80E-08	2.10E-06
APOBEC3A	7.222622	15.39567	1.09193	0.000117	0.00086
GNLY	5.746498	15.71254	1.451161	5.90E-18	1.87E-15
FAM81B	1.156723	0.266055	-2.12024	0.02264	0.054976
HS3ST6	3.761969	1.099181	-1.77506	0.000775	0.003912
RNF180	0.317943	0.110466	-1.52517	1.35E-05	0.000145
CORIN	0.157573	0.065432	-1.26795	0.000288	0.001786
ADRA2C	5.991443	2.14956	-1.47886	0.00286	0.010924
GALNT17	0.259697	0.119012	-1.12572	0.002226	0.008949
CD209	0.414886	1.061605	1.355461	6.13E-10	2.22E-08
CXCL11	4.634704	31.65799	2.772021	1.31E-19	6.62E-17
WT1	1.18435	0.22407	-2.40207	0.00081	0.004033
LYPD8	0.076998	0.733297	3.251499	0.045919	0.094755

Gene	conMean	treatMean	logFC	pValue	FDR
G0S2	20.71791	47.37173	1.193148	0.001299	0.005902
MYEF2	0.789113	0.203601	-1.95449	0.021515	0.05274
NME5	0.586632	0.1852	-1.66337	0.000333	0.002002
FYB1	2.334689	4.832451	1.049525	4.55E-11	2.14E-09
OR2I1P	3.743256	11.69244	1.643211	1.16E-15	1.94E-13
CBARP	0.426829	0.196835	-1.11667	4.67E-07	8.06E-06
SLC35D3	0.214274	0.091716	-1.22421	8.89E-08	1.93E-06
SHISA9	0.396786	0.076519	-2.37447	1.33E-05	0.000142
CTSS	17.81972	36.69746	1.042206	1.18E-11	6.33E-10
CCR1	1.744056	3.512075	1.009877	1.01E-10	4.45E-09
RBM24	0.334694	0.115811	-1.53107	9.52E-05	0.000727
PEG10	9.368488	3.399231	-1.46261	2.83E-06	3.89E-05
TBX21	0.327901	1.143982	1.80273	6.11E-17	1.41E-14
PPP1R1B	24.6912	7.065922	-1.80505	7.48E-05	0.0006
FGF18	1.653745	0.4228	-1.96769	8.80E-07	1.42E-05
PGM5	0.902487	0.238063	-1.92257	0.000184	0.00125
MYO1F	1.553418	3.149509	1.019681	4.70E-13	3.50E-11
CAMK2B	1.379855	0.396449	-1.79931	0.026595	0.062293
GPR174	0.233382	0.806739	1.789408	8.91E-14	8.05E-12
BCHE	0.531949	0.168775	-1.65619	3.52E-05	0.000323
TDGF1	0.407873	0.07432	-2.4563	0.013955	0.037379
MEP1A	0.181215	0.860286	2.247117	4.05E-05	0.000362
CYP2A13	0.761548	0.079235	-3.26472	0.000526	0.00286
TAC1	0.837953	0.040899	-4.35673	0.000296	0.001827
DPYSL3	18.0221	6.617535	-1.4454	1.31E-09	4.43E-08

Gene	conMean	treatMean	logFC	pValue	FDR
PCDH19	0.878734	0.294162	-1.57882	8.69E-13	5.99E-11
IGFL2	1.678604	0.689216	-1.28423	0.018944	0.047618
RXFP4	0.165941	0.07514	-1.14302	0.036553	0.07995
TGM3	9.55489	1.613534	-2.56602	0.004462	0.015396
FAM171A2	2.958583	1.298021	-1.18859	1.45E-06	2.18E-05
FOXN4	0.458173	0.045934	-3.31827	1.41E-06	2.13E-05
LGR5	2.260448	0.532593	-2.0855	0.000284	0.001765
TCL1A	0.143728	0.386253	1.426202	5.77E-05	0.000487
GRIK2	0.161365	0.061773	-1.38528	4.08E-05	0.000364
GSTA4	11.03167	5.203211	-1.08418	1.54E-07	3.08E-06
GPA33	0.629255	2.425972	1.946846	0.000159	0.001109
ROS1	0.554807	0.235519	-1.23614	0.006322	0.020235
FPR2	0.154439	0.32532	1.074818	0.00319	0.011866
PALM3	3.444929	1.544721	-1.15713	0.010126	0.029118
TCEAL2	0.892684	0.258638	-1.78722	0.000567	0.003044
ALOX15	11.40115	1.466282	-2.95894	0.000707	0.003648
SIT1	1.26252	3.358815	1.411647	2.72E-13	2.17E-11
NOV	4.444801	1.920166	-1.21089	1.25E-06	1.93E-05
PCDHB8	0.473145	0.23117	-1.03333	0.007654	0.02349
KCNJ10	0.16526	0.400547	1.277232	4.33E-06	5.59E-05
FZD8	5.356032	2.496171	-1.10145	4.64E-05	0.000406
PCP4	4.968776	0.576329	-3.10793	0.000368	0.002162
ANKRD45	0.559339	0.080396	-2.79853	2.99E-06	4.08E-05
UPK1B	10.86521	2.751228	-1.98157	4.47E-07	7.78E-06
ENPP1	0.828731	0.402275	-1.04272	3.94E-06	5.17E-05

Gene	conMean	treatMean	logFC	pValue	FDR
RAC2	14.18712	30.40653	1.0998	9.50E-20	5.19E-17
IGFL3	4.123209	0.381444	-3.43422	0.00109	0.005136
KIF1A	1.960051	0.562416	-1.80118	0.006311	0.020222
CACNA1B	0.400225	0.158611	-1.33532	0.008765	0.026101
TBC1D10C	1.373152	3.390021	1.303802	3.35E-14	3.52E-12
KIAA1324	11.35262	4.648182	-1.28829	0.000219	0.001443
SCIMP	0.403733	0.882217	1.127733	2.26E-07	4.31E-06
NOTUM	14.12782	0.751422	-4.23277	0.004311	0.014981
FAM171B	1.053336	0.502557	-1.06761	7.48E-05	0.0006
WNT16	0.321997	0.120513	-1.41786	0.001836	0.007721
USP6	0.139203	0.068494	-1.02315	0.032364	0.072723
SLC10A4	0.739818	0.295325	-1.32487	0.018812	0.047367
STMND1	0.310782	0.094857	-1.71207	0.019109	0.047937
CCRL2	0.617443	1.255537	1.023927	3.05E-12	1.89E-10
PAX7	2.309165	0.860318	-1.42443	0.00051	0.002786
ZNF667	0.371654	0.180148	-1.04477	6.93E-05	0.000567
ALX1	0.796526	0.387149	-1.04083	0.000426	0.002416
FZD7	8.069833	3.669287	-1.13704	2.81E-08	6.92E-07
TPPP3	15.5435	6.963815	-1.15836	1.08E-08	2.95E-07
MSI1	3.122773	0.788433	-1.98577	1.86E-06	2.70E-05
FCGR3A	9.665443	22.43742	1.214999	1.65E-09	5.45E-08
LY6G6D	1.607146	0.042599	-5.23754	0.00015	0.00105
SCUBE2	1.563666	0.693967	-1.17199	6.18E-05	0.000516
ZNF474	0.619496	0.170069	-1.86498	0.00013	0.000935
METTL24	0.286136	0.064838	-2.14178	0.000415	0.002364

Gene	conMean	treatMean	logFC	pValue	FDR
PACRG	0.478764	0.117029	-2.03244	1.53E-05	0.00016
SIRPB1	0.151694	0.348887	1.201598	2.21E-10	8.97E-09
ERBB4	0.327875	0.159419	-1.04032	0.000215	0.001421
HLA-F	28.03395	61.47502	1.132825	2.60E-19	1.26E-16
FGF13	0.160709	0.045697	-1.81427	1.94E-05	0.000196
KYNU	1.495735	3.00967	1.008751	4.10E-08	9.73E-07
FMO2	2.42776	0.78255	-1.63337	0.009791	0.028418
IFIT1	6.794643	15.66303	1.204894	5.58E-12	3.25E-10
BAALC	3.039649	1.236609	-1.29752	7.68E-05	0.000613
FAM227A	0.445605	0.217858	-1.03238	5.18E-05	0.000444
CLNK	0.080383	0.207563	1.368579	7.39E-12	4.23E-10
DMRT1	0.492179	0.099539	-2.30585	1.87E-06	2.71E-05
EFHC2	1.236563	0.480847	-1.36269	0.011872	0.032951
C20orf141	0.100575	0.47633	2.243695	2.00E-06	2.87E-05
CITED1	1.046802	0.173867	-2.58994	7.36E-05	0.000592
THSD7B	0.183274	0.046387	-1.98221	3.20E-06	4.32E-05
CHRDL1	0.753471	0.232539	-1.69608	0.000951	0.004605
C1orf194	2.404215	0.682097	-1.81752	7.14E-06	8.45E-05
SLFN12L	0.229497	0.556578	1.27811	1.65E-15	2.56E-13
PPEF1	0.198916	0.069353	-1.52013	4.12E-08	9.77E-07
PRSS35	0.205544	0.075527	-1.44439	1.43E-05	0.000152
CLEC10A	1.010625	2.232518	1.143424	1.79E-05	0.000183
CCL4L2	2.56018	5.922525	1.209967	8.71E-09	2.42E-07
NTRK2	4.984757	1.844033	-1.43466	0.027129	0.06324
SAA1	38.90768	117.6027	1.595794	3.10E-11	1.53E-09

Gene	conMean	treatMean	logFC	pValue	FDR
DSG4	0.358716	0.150292	-1.25507	0.022693	0.055093
CXCL9	15.12839	58.05872	1.940255	2.79E-11	1.39E-09
PLA2G2D	0.833703	3.199887	1.940415	0.000304	0.001866
SPDYC	1.809995	0.618242	-1.54974	0.04371	0.091302
OASL	7.348736	15.9724	1.120013	3.92E-13	2.97E-11
TBX2	1.699362	0.778166	-1.12684	0.001358	0.006123
UBD	7.231495	21.12984	1.546916	1.99E-14	2.21E-12
ASPRV1	5.773996	0.98001	-2.5587	0.026595	0.062293
WAS	3.140101	6.437185	1.035619	1.23E-11	6.55E-10
OTOF	0.061954	0.145144	1.228213	4.36E-10	1.63E-08
IFIT2	4.480862	11.52559	1.362993	2.53E-17	6.64E-15
CD38	1.049503	2.401974	1.194514	1.83E-07	3.57E-06
BIRC3	6.502175	22.96755	1.820603	8.77E-12	4.88E-10
POU3F2	0.623002	0.03041	-4.35662	4.87E-05	0.000422
ETV4	9.627612	3.632995	-1.40602	5.40E-08	1.24E-06
CHIT1	0.934414	2.572745	1.461174	0.039174	0.084013
HAND2	0.70365	0.256711	-1.45471	0.005294	0.017584
DLX6	1.961663	0.636156	-1.62463	0.000113	0.000836
NRCAM	2.03861	0.617939	-1.72205	0.003021	0.011364
CXXC4	0.391383	0.186256	-1.0713	2.01E-05	0.000202
TMEM150B	0.442226	1.540327	1.800381	4.71E-10	1.74E-08
MMP16	0.181783	0.078181	-1.21733	0.000288	0.001786
CLEC4E	0.479028	1.231847	1.36264	3.92E-08	9.35E-07
LAX1	0.410935	0.963435	1.229279	4.45E-07	7.74E-06
TNNI2	6.505012	18.55581	1.512247	0.000245	0.001581

Gene	conMean	treatMean	logFC	pValue	FDR
COL14A1	3.179772	1.331073	-1.25633	0.001459	0.006479
ZNF683	1.3242	5.876746	2.149896	8.33E-19	3.45E-16
COL10A1	4.525136	1.869759	-1.27511	0.000121	0.000883
KLRK1	0.169052	0.370836	1.133315	7.82E-09	2.20E-07
CCL25	0.359669	2.040294	2.504037	0.000145	0.001021
CD27	2.42841	5.64545	1.217077	1.81E-09	5.94E-08
EOMES	0.372794	0.969492	1.378852	4.13E-07	7.23E-06
LEFTY2	1.088074	0.307268	-1.82421	1.51E-05	0.00016
NTF3	0.486488	0.150065	-1.69681	3.39E-05	0.000313
TMEM211	1.209877	0.459949	-1.39532	8.33E-08	1.82E-06
ODC1	77.81315	37.38217	-1.05766	0.002673	0.010319
RASD1	22.8172	10.10093	-1.17563	0.001961	0.008139
FCGR1B	0.126204	0.274945	1.123387	4.64E-08	1.08E-06
ETV5	5.44107	2.627252	-1.05034	0.000492	0.002704
ZNF831	0.08958	0.234464	1.388117	7.16E-08	1.59E-06
FREM2	0.710404	0.170146	-2.06187	7.17E-07	1.19E-05
IQUB	0.144249	0.066013	-1.12773	0.002079	0.008499
CRTAM	0.326914	1.081025	1.725418	2.22E-11	1.12E-09
TOX3	2.658653	0.759025	-1.80848	0.000117	0.000858
DIRAS2	0.646706	0.084609	-2.93423	0.000558	0.003001
SHH	1.690297	0.518427	-1.70506	0.008301	0.025007
BCL2A1	3.383471	7.33095	1.115496	4.87E-10	1.80E-08
IRF4	0.672084	1.556009	1.211137	1.81E-08	4.71E-07
NEK5	0.342161	0.151777	-1.17272	0.000336	0.002014
TTPA	0.287137	0.101878	-1.49489	7.77E-06	9.10E-05

Gene	conMean	treatMean	logFC	pValue	FDR
FAM3B	8.948917	4.398759	-1.02462	3.80E-05	0.000344
CCL23	0.271848	0.618091	1.185022	0.000206	0.001376
KAAG1	0.161537	0.062736	-1.3645	0.004717	0.016041
CCL3	2.983408	6.637427	1.153663	4.21E-12	2.50E-10
ACHE	0.865448	2.483364	1.520776	2.60E-05	0.000251
CARD18	0.788308	0.233958	-1.75251	0.020103	0.049998
IGFBP2	56.05919	20.8458	-1.42719	5.94E-12	3.45E-10
PSMB9	27.33596	58.76397	1.104132	3.32E-19	1.56E-16
GPR18	0.242061	0.72186	1.576349	1.03E-15	1.78E-13
LRP2	0.229649	0.105767	-1.11853	0.023161	0.055961
ICAM1	11.9338	27.63994	1.211702	2.53E-15	3.79E-13
BTLA	0.15044	0.309994	1.04305	4.90E-06	6.19E-05
CA8	1.46251	0.26672	-2.45505	0.005842	0.019008
ADAMTS19	0.451453	0.015814	-4.83526	2.54E-06	3.55E-05
FAM180A	0.225848	0.108602	-1.0563	0.000501	0.002747
ISL2	1.68106	0.786673	-1.09554	0.003124	0.011665
TNFRSF17	0.892062	1.849572	1.051976	0.000248	0.001592
LEP	0.119656	0.58663	2.293553	0.004664	0.015904
ID4	9.604498	3.715736	-1.37006	7.58E-10	2.70E-08
IL1A	4.580821	10.27244	1.165101	0.004867	0.016445
PDLIM3	1.364673	0.62926	-1.11683	0.000317	0.001924
HK3	1.212699	2.675014	1.141325	3.38E-11	1.65E-09
PROX1	0.557823	0.105332	-2.40486	0.000784	0.003936
RERGL	0.450872	0.218327	-1.04623	0.000514	0.002806
GLYATL2	5.591524	1.40523	-1.99244	7.08E-05	0.000577

Gene	conMean	treatMean	logFC	pValue	FDR
DRD2	0.365317	0.150727	-1.27721	7.69E-06	9.02E-05
CSF2RB	1.512017	3.546724	1.230012	5.27E-07	8.98E-06
TEKT2	3.423768	0.933359	-1.87508	0.039918	0.085113
COMP	17.55516	6.585867	-1.41445	7.28E-05	0.00059
STAT4	0.714144	1.562748	1.129799	1.28E-16	2.73E-14
COL3A1	194.7028	95.77704	-1.02352	0.000399	0.002301
RASAL3	1.613644	3.803189	1.236887	1.50E-14	1.75E-12
BMP5	0.405405	0.039124	-3.37322	0.002504	0.009793
MMP12	12.83658	28.38776	1.145009	4.02E-07	7.05E-06
FGF9	0.90986	0.123793	-2.87772	5.06E-06	6.36E-05
SMAD9	1.340356	0.474845	-1.49709	4.78E-06	6.08E-05
SPOCK2	3.920205	8.913415	1.185049	2.40E-06	3.38E-05
NDRG4	5.881862	2.412942	-1.28548	8.07E-06	9.41E-05
ACSS3	0.365587	0.084199	-2.11834	0.002139	0.00869
LIN7A	0.465121	0.134175	-1.79349	0.002326	0.009281
PRR36	3.178401	0.889891	-1.8366	2.32E-10	9.30E-09
CDH26	2.237914	0.85392	-1.38998	3.84E-05	0.000347
LCK	3.007513	6.071531	1.013489	1.58E-10	6.58E-09
CAPN6	3.621615	0.673316	-2.42728	0.000253	0.001621
RNASE2	0.633172	1.308395	1.047128	1.24E-09	4.23E-08
C3	35.70686	71.84968	1.008781	8.73E-06	0.000101
ICAM3	0.353473	0.815207	1.205566	5.43E-12	3.18E-10
SLAMF7	2.581105	6.674062	1.370576	6.00E-11	2.76E-09
DIRAS1	0.878241	0.396494	-1.14732	0.001011	0.004836
ASPN	5.204186	2.512731	-1.05042	0.003124	0.011665

Gene	conMean	treatMean	logFC	pValue	FDR
DNAJB13	0.632325	0.303204	-1.06038	0.01245	0.034125
ITGA11	1.752819	0.810622	-1.11258	9.68E-05	0.000737
GSTA1	19.72777	1.254358	-3.97521	2.12E-05	0.000211
LGALS2	3.101841	7.031363	1.18068	8.28E-05	0.000651
SEMA3D	0.40355	0.131519	-1.61747	0.000351	0.002086
CD247	1.364141	3.576199	1.390435	2.32E-18	8.12E-16
HSD17B13	0.542603	0.135112	-2.00574	0.04609	0.095035
SAA2	3.169806	8.013245	1.337992	6.86E-09	1.96E-07
FAM78A	0.926926	1.885252	1.024231	5.00E-11	2.33E-09
GIMAP5	0.095269	0.215358	1.176666	1.77E-08	4.62E-07
DNALI1	5.969957	2.288513	-1.38331	0.000588	0.00313
BMP4	5.643238	1.685615	-1.74325	0.001275	0.005806
TYMP	66.78722	136.012	1.02609	2.04E-16	4.15E-14
SPEF1	0.943842	0.291947	-1.69284	7.50E-05	0.000601
MORN5	0.991122	0.192728	-2.3625	2.69E-05	0.000258
PRLR	1.037723	0.451914	-1.1993	0.003908	0.013836
SFRP2	46.77034	19.88843	-1.23366	0.03289	0.073493
PCDHB6	0.242646	0.082298	-1.55992	0.002829	0.010837
C11orf88	0.186518	0.039392	-2.24334	0.000602	0.003192
EMX2	3.7064	1.015123	-1.86836	0.002836	0.010854
FAM181A	0.807866	0.048181	-4.06757	0.000406	0.002322
LTB	7.192876	18.187	1.338267	3.57E-12	2.16E-10
CAMP	2.781065	0.505946	-2.45858	0.031114	0.07053
CLPSL2	0.426837	0.19915	-1.09983	0.019009	0.047748
CD37	2.427708	4.910445	1.016259	4.81E-09	1.43E-07

Gene	conMean	treatMean	logFC	pValue	FDR
DOCK2	0.679201	1.467338	1.11129	1.43E-10	6.06E-09
IGFL4	0.210321	0.042291	-2.31418	1.63E-05	0.000169
MAP1B	2.55026	1.222165	-1.06121	0.001704	0.00728
C5orf46	3.208891	0.853456	-1.91069	0.004572	0.015684
NAT8	0.052544	0.179383	1.771437	0.048292	0.098416
SPTSSB	7.468454	2.874117	-1.37769	2.52E-06	3.52E-05
GIMAP4	5.113274	10.44509	1.030506	1.26E-08	3.39E-07
PDE6A	0.179059	0.066841	-1.42163	0.000221	0.001451
MMP28	8.616603	17.89898	1.054687	1.30E-07	2.68E-06
SEMA6D	0.887033	0.309748	-1.51789	0.015842	0.041265
TFEC	0.287667	0.634302	1.140767	2.65E-08	6.56E-07
SLC9A4	0.328456	0.053861	-2.60838	9.61E-08	2.06E-06
DSC1	0.248657	0.08519	-1.54541	0.005622	0.018421
HLA-DOB	1.934362	4.224704	1.126993	1.29E-13	1.12E-11
SLITRK6	5.608246	1.622656	-1.78919	0.003892	0.0138
CAPSL	1.39716	0.212412	-2.71756	0.047063	0.096386
ACTC1	0.332194	0.092397	-1.84611	0.0001	0.000758
CXCR6	1.165205	3.543107	1.604431	2.78E-15	4.03E-13
FZD3	0.86327	0.376469	-1.19728	1.41E-06	2.13E-05
TIGIT	0.816769	2.269642	1.474464	6.08E-15	7.97E-13
KCNH3	1.594964	0.461107	-1.79035	0.000944	0.00458
HS6ST3	0.186534	0.074678	-1.32067	8.24E-06	9.57E-05
CD6	1.424909	2.856162	1.003208	3.46E-09	1.06E-07
FBXO15	0.152774	0.076273	-1.00216	0.001505	0.006636
ARHGAP6	0.323409	0.140355	-1.20428	0.000241	0.001561

Gene	conMean	treatMean	logFC	pValue	FDR
DRC1	1.495065	0.392757	-1.9285	0.046753	0.095968
XCL2	0.995136	2.653458	1.414909	1.12E-14	1.39E-12
ADAMTS16	0.343991	0.14905	-1.20657	0.000219	0.001442
IL15	1.16654	2.335514	1.001504	2.09E-14	2.31E-12
OGN	1.148799	0.239354	-2.26291	0.002004	0.00827
IRF1	12.41263	27.33399	1.138887	4.19E-24	2.86E-20
CST1	16.2933	6.531786	-1.31873	0.017066	0.043793
CD8A	3.148888	9.584449	1.605853	3.06E-13	2.40E-11
CXCL10	35.87419	177.7255	2.308633	3.92E-16	7.65E-14
FAM69B	2.482292	0.915901	-1.43841	0.001236	0.005671
PIANP	0.650422	0.227708	-1.51419	4.69E-06	6.00E-05
PLEK	3.633924	8.363366	1.202556	5.36E-09	1.57E-07
ART5	0.456933	0.17132	-1.41529	3.37E-06	4.52E-05
SOX5	0.294881	0.080103	-1.88021	1.71E-07	3.35E-06
C1QL4	0.83417	0.234586	-1.83023	7.75E-06	9.08E-05
UNC93A	0.757418	0.268908	-1.49398	0.00069	0.003576
EBI3	1.17615	2.368492	1.009897	4.05E-09	1.23E-07
LCP2	2.012964	4.400183	1.128242	3.56E-14	3.68E-12
ZIC1	0.303028	0.116698	-1.37668	0.027594	0.06405
KIT	2.500969	0.968669	-1.36841	0.003735	0.013355
KRT40	0.486526	0.054933	-3.14678	0.001693	0.007252
NOG	0.640818	0.227601	-1.49341	0.002451	0.00965
PREX2	0.47858	0.235976	-1.02012	0.029262	0.067145
CLSTN2	0.510464	0.20403	-1.32303	1.06E-06	1.67E-05
FREM1	0.421235	0.127658	-1.72234	4.85E-05	0.000421

Gene	conMean	treatMean	logFC	pValue	FDR
CIITA	2.252468	5.135471	1.18899	2.04E-13	1.69E-11
HLA-DMA	31.48118	63.22623	1.006033	8.31E-12	4.70E-10
STK33	0.303497	0.12789	-1.24678	0.002052	0.008414
LILRB2	0.919021	2.038327	1.149216	1.35E-11	7.13E-10
KIAA0319	0.851697	0.239295	-1.83155	2.07E-07	3.97E-06
CCL13	1.431538	3.362398	1.231924	2.32E-08	5.84E-07
VPS37D	1.847826	0.792709	-1.22097	1.21E-06	1.86E-05
CASKIN1	0.247522	0.111787	-1.14681	3.76E-06	4.97E-05
CRYM	2.793457	0.76004	-1.8779	2.71E-06	3.74E-05
CASP14	34.75189	90.43533	1.379795	3.18E-07	5.78E-06
NUGGC	0.14984	0.461823	1.623922	7.58E-10	2.70E-08
PLEKHG4B	1.474333	0.712311	-1.04948	0.00386	0.01372
LONRF2	0.377835	0.099555	-1.92419	5.15E-05	0.000441
KIR2DL4	0.535552	1.624966	1.601311	3.65E-19	1.66E-16
GBP1	18.0899	49.22861	1.444313	1.79E-15	2.75E-13
TRAT1	0.268738	0.814277	1.599318	3.79E-11	1.82E-09
IL12RB1	0.642901	1.851731	1.526206	3.01E-20	2.42E-17
NKD1	2.945604	0.244754	-3.58916	0.045048	0.093453
RNFT2	1.179272	0.571171	-1.0459	4.96E-07	8.52E-06
CD3G	0.648792	1.944898	1.583867	4.26E-14	4.21E-12
SIGLEC1	1.10338	2.59583	1.234265	2.67E-11	1.33E-09
NGB	3.019934	1.137936	-1.4081	0.000268	0.001689
UTS2	0.156223	0.358907	1.200002	1.50E-05	0.000159
BIRC2	12.39273	36.00026	1.538513	0.000662	0.003454
CYTIP	2.107226	4.431176	1.072344	4.45E-10	1.66E-08

Gene	conMean	treatMean	logFC	pValue	FDR
CCL8	1.161292	3.887261	1.743023	4.47E-11	2.12E-09
GPR25	0.252342	0.813882	1.689438	8.08E-14	7.40E-12
PLCXD3	0.198252	0.018436	-3.42673	0.001582	0.006895
ATP6V1C2	5.425806	2.47463	-1.13262	0.000292	0.001803
FYB2	0.35595	0.157387	-1.17736	0.002068	0.008465
RGS17	0.490517	0.222713	-1.13912	3.12E-08	7.56E-07
DISP3	0.662229	0.286987	-1.20635	0.001421	0.006348
RIIAD1	0.152211	0.047715	-1.67357	0.031584	0.071334
MYO7A	0.799615	1.6053	1.005466	1.55E-11	8.10E-10
CDO1	0.497032	0.180008	-1.46528	0.001615	0.006985
HLA-DRA	424.2822	976.2032	1.202157	1.29E-13	1.12E-11
KLHL14	1.184495	0.435365	-1.44397	0.001991	0.008242
ACAP1	1.499036	3.070083	1.034243	1.22E-12	8.15E-11
LRIG1	11.88331	5.650559	-1.07247	4.39E-07	7.65E-06
TMSB15A	10.127	3.201392	-1.66144	5.93E-08	1.34E-06
RSAD2	5.038559	11.9928	1.251086	1.42E-12	9.33E-11
PTH1R	0.476284	0.237142	-1.00607	0.000745	0.003793
UBASH3A	0.447685	1.278209	1.513567	5.03E-18	1.65E-15
CD19	0.408235	0.881741	1.110955	0.000291	0.0018
TIFAB	0.081765	0.202408	1.307707	1.57E-07	3.13E-06
ARHGAP15	0.413573	0.864283	1.063362	2.98E-09	9.36E-08
IFIH1	7.428755	15.307	1.042999	3.89E-21	4.82E-18
NUDT10	0.548559	0.232329	-1.23948	0.008776	0.026127
DLEC1	0.412147	0.106217	-1.95614	0.046735	0.095959
GPR173	0.740892	0.355703	-1.05859	0.001214	0.00559

Gene	conMean	treatMean	logFC	pValue	FDR
CABYR	1.683276	0.829984	-1.02012	3.13E-05	0.000294
SCN5A	0.166899	0.06668	-1.32366	0.021078	0.05195
ZBP1	0.642297	2.031893	1.661512	2.17E-18	7.99E-16
ITGA2B	1.625677	0.784454	-1.05128	0.002765	0.010626
FGF17	0.30055	0.118751	-1.33967	3.08E-05	0.00029
C1S	23.48103	47.63949	1.020662	2.09E-08	5.33E-07
COLGALT2	0.610762	0.239744	-1.34911	1.37E-05	0.000147
ETV7	6.531705	15.00726	1.200129	2.70E-20	2.30E-17
CES1	28.58585	9.379421	-1.60773	0.011786	0.032743
IL18RAP	0.358593	0.872462	1.282746	3.81E-17	9.11E-15
SCGB3A2	0.237963	0.061318	-1.95634	9.75E-06	0.000111
POSTN	25.46021	6.306544	-2.01332	2.57E-05	0.000249
CALB2	6.575882	2.461644	-1.41756	0.000872	0.004296
INHBB	5.279989	2.106521	-1.32567	0.000223	0.001456
AIM2	7.192779	22.90124	1.670804	4.21E-14	4.21E-12
AQP5	62.49345	27.05939	-1.20758	0.002075	0.008483
CALB1	6.326681	2.869782	-1.14051	0.039415	0.084343
C1QC	51.62397	128.6761	1.317631	2.01E-11	1.03E-09
CFAP52	0.319868	0.131913	-1.27789	0.008878	0.026385
UMODL1	0.230265	0.049889	-2.2065	0.001717	0.007324
PLA2G5	0.146491	0.050717	-1.53027	0.013341	0.035988
TCF21	0.119431	0.052385	-1.18896	0.018263	0.046212
IGLL5	12.52671	27.72725	1.146297	0.000852	0.004211
SLC1A3	3.626427	8.346191	1.202569	1.88E-06	2.72E-05
FAM174B	6.021047	2.764209	-1.12315	2.17E-06	3.08E-05

Gene	conMean	treatMean	logFC	pValue	FDR
NCF1	0.536455	1.257399	1.228913	2.47E-13	2.00E-11
RIMBP2	0.551714	0.238304	-1.21112	0.000323	0.001954
SCN9A	0.50029	0.174463	-1.51984	0.001727	0.007358
AOAH	1.373964	2.936822	1.095911	3.30E-09	1.02E-07
MICU3	0.284545	0.106957	-1.41163	0.012702	0.034662
CERS1	0.263165	0.079956	-1.7187	0.006501	0.020638
UGT2A1	0.603813	0.015458	-5.28768	1.35E-07	2.75E-06
PI16	1.091944	0.484018	-1.17377	0.034505	0.076429
ANKRD35	7.183325	3.118969	-1.20358	7.89E-05	0.000625
SCG2	0.488728	0.161977	-1.59324	0.000291	0.0018
MAATS1	0.993899	0.389606	-1.35108	0.012633	0.034515
ASCL5	0.572686	0.111778	-2.3571	7.29E-05	0.00059
ZBBX	0.636861	0.081584	-2.96463	0.003645	0.013087
IL9R	0.131762	1.241267	3.235812	7.09E-15	9.21E-13
DNAH6	0.206251	0.091947	-1.16552	0.005315	0.017624
TAP2	10.13528	20.51371	1.017203	1.38E-22	2.69E-19
BATF2	2.992387	8.397899	1.488732	8.63E-21	7.85E-18
CD72	0.770256	1.60147	1.055986	1.17E-06	1.82E-05
GIMAP7	3.765061	8.118724	1.10858	5.12E-09	1.51E-07
WDR49	0.563419	0.095583	-2.55939	9.29E-05	0.000714
GPRC5B	3.732901	1.633213	-1.19258	7.98E-05	0.00063
MYLK	3.20937	1.560395	-1.04038	0.003298	0.012173
P2RY13	0.582912	1.295015	1.151618	9.13E-08	1.97E-06
MYH11	5.661791	1.444883	-1.97031	1.61E-05	0.000167
IGSF10	0.275676	0.12331	-1.16069	3.47E-07	6.21E-06

Gene	conMean	treatMean	logFC	pValue	FDR
CYP2F1	0.339882	0.124097	-1.45357	0.000104	0.000782
L1CAM	1.492284	4.862069	1.704048	0.012541	0.034333
HLA-DPB1	62.77396	129.8346	1.048437	4.43E-11	2.11E-09
BMP3	1.889746	0.916866	-1.04341	0.001333	0.006035
CTTNBP2	0.619003	0.287195	-1.10792	4.54E-07	7.87E-06
CCDC160	1.564273	0.642148	-1.28451	0.02361	0.056813
DPP6	0.115831	0.030785	-1.91172	0.010222	0.02935
HERC6	5.159074	11.75689	1.188323	4.26E-14	4.21E-12
TMEM215	0.341383	0.131339	-1.3781	0.015112	0.039789
EYA1	0.744159	0.109755	-2.76133	6.78E-06	8.05E-05
CPLX2	0.47125	0.018606	-4.66263	0.000373	0.002181
LAIR1	1.433451	2.932833	1.032802	2.74E-09	8.64E-08
LILRB4	1.419813	3.373459	1.248528	9.94E-08	2.13E-06
TUBA4B	0.631335	0.19477	-1.69663	0.047592	0.097233
SGCD	0.584913	0.184916	-1.66135	5.09E-07	8.74E-06
ARHGEF26	3.380718	1.689489	-1.00074	2.96E-08	7.25E-07
TTC6	0.458603	0.225963	-1.02116	0.003614	0.013003
CLEC12A	0.340513	0.75466	1.148115	2.32E-07	4.42E-06
MFSD6L	1.507491	0.582144	-1.3727	0.017179	0.044005
CRMP1	3.699981	1.803784	-1.03649	0.000362	0.002134
GZMH	3.147316	11.06334	1.813593	3.67E-13	2.79E-11
EXOC3L4	1.527835	3.493417	1.19315	9.54E-09	2.64E-07
NTS	154.8042	57.09218	-1.43908	0.000189	0.001274
DYNC1I1	0.855716	0.175892	-2.28244	2.65E-06	3.69E-05
P2RY10	0.446283	1.054428	1.24043	4.13E-10	1.56E-08

Gene	conMean	treatMean	logFC	pValue	FDR
PGAP3	26.30526	11.88683	-1.14599	3.69E-05	0.000336
PLEKHD1	0.343431	0.083491	-2.04033	9.84E-07	1.56E-05
CNGB1	0.261749	0.644455	1.299898	0.000447	0.002505
EMID1	3.904285	1.381862	-1.49844	0.012496	0.034236
BOC	2.116509	0.93272	-1.18217	1.02E-07	2.16E-06
RCOR2	4.229942	1.190782	-1.82873	7.31E-08	1.62E-06
ACTA2	49.9347	21.1601	-1.2387	2.73E-05	0.000262
GZMB	8.465743	21.32419	1.332782	7.91E-16	1.42E-13
ADRA1B	0.342829	0.143675	-1.25468	0.001144	0.005338
GBP4	7.935943	22.76427	1.520298	2.99E-17	7.27E-15
SLC26A5	0.197421	0.073635	-1.42282	2.86E-11	1.42E-09
ENPP6	0.119397	0.303534	1.346097	0.000243	0.001567
RAB3B	0.669256	0.228441	-1.55074	0.011465	0.032082
CHRNA9	0.502264	0.201464	-1.31792	0.000828	0.004105
PTPRC	2.663742	6.27308	1.23572	2.53E-11	1.27E-09
LDB3	0.487042	0.122539	-1.99081	0.003298	0.012173
HAVCR2	2.405392	5.290645	1.137172	1.61E-12	1.05E-10
HES6	32.98473	7.154163	-2.20494	4.25E-09	1.28E-07
CCDC40	0.845813	0.371467	-1.18711	0.002909	0.011073
STK32A	0.171834	0.049159	-1.80548	3.25E-05	0.000302
SLC38A3	0.434472	0.215321	-1.01277	0.012679	0.034613
WIF1	4.45463	0.153387	-4.86006	2.16E-05	0.000214
HOXC12	0.954328	0.15266	-2.64416	0.014481	0.03849
SLC9A2	1.075452	0.400102	-1.4265	2.65E-06	3.69E-05
TNFRSF19	4.522415	1.432911	-1.65814	1.24E-05	0.000135

Gene	conMean	treatMean	logFC	pValue	FDR
GDNF	0.352761	0.145869	-1.27402	1.49E-06	2.23E-05
STUM	0.211108	0.060269	-1.80849	0.002399	0.009501
TUBA3E	0.710847	0.016225	-5.45321	4.20E-05	0.000372
DPY19L2	0.449272	0.217489	-1.04665	0.001519	0.006681
SYT1	0.807561	0.391434	-1.0448	0.037485	0.081414
MARCO	2.584523	5.797446	1.165519	0.000157	0.001098
CFAP77	0.675682	0.142894	-2.2414	0.00136	0.006128
MEX3A	3.994915	1.560428	-1.35622	4.19E-08	9.86E-07
PCYT1B	0.312632	0.103363	-1.59675	1.73E-07	3.38E-06
CBLN1	0.805946	0.372328	-1.11411	0.020049	0.049917
DNAAF1	0.265812	0.109103	-1.28472	9.83E-05	0.000746
CXCL2	7.363816	15.74356	1.096237	0.000144	0.00102
C1QTNF7	0.226716	0.100434	-1.17465	0.001334	0.006035
HLA-DRB5	80.02776	230.1504	1.524005	4.61E-14	4.46E-12
TMEM178A	3.760497	0.285764	-3.71803	4.49E-05	0.000393
LY6G6F-LY6G6D	0.710754	0.027629	-4.68508	0.000385	0.002238
HLA-DRB1	257.3869	635.0574	1.302948	2.66E-14	2.82E-12
CHGA	4.352971	1.0246	-2.08694	0.024818	0.058962
GREM2	0.395921	0.090594	-2.12772	0.009824	0.028506
FGFR1	5.73788	1.904354	-1.59122	5.44E-08	1.24E-06
WFDC1	0.840748	0.366774	-1.19678	0.000125	0.000904
WNT11	4.088836	1.451157	-1.49449	0.000214	0.001415
FGF12	0.392297	0.178751	-1.13399	3.76E-07	6.67E-06
GDF5	0.525737	0.216238	-1.28172	0.020204	0.050222

Gene	conMean	treatMean	logFC	pValue	FDR
DACT1	1.381269	0.665106	-1.05434	2.66E-05	0.000257
C5orf49	2.055269	0.60556	-1.76299	0.016491	0.042639
RDH12	1.932757	0.63512	-1.60556	0.01709	0.043848
TRAF3IP3	0.701	1.445444	1.044027	2.89E-12	1.82E-10
PRF1	3.498229	12.28794	1.812547	3.56E-20	2.56E-17
PNMT	49.34789	2.134445	-4.53106	3.58E-05	0.000327
KIF26B	1.499425	0.731258	-1.03596	3.19E-05	0.000297
CFAP45	3.831958	1.114292	-1.78195	3.56E-07	6.36E-06
TMEM100	1.468424	0.121398	-3.59645	7.31E-05	0.000591
COCH	4.098478	2.044125	-1.00361	0.006866	0.02157
SLC51B	0.685718	0.303051	-1.17805	0.025195	0.05967
ELF5	2.252458	0.875432	-1.36343	8.21E-07	1.33E-05
CASP5	0.17292	0.393858	1.18757	2.75E-15	4.03E-13
DUOXA2	4.557249	10.08209	1.14556	5.89E-06	7.21E-05
CD53	9.696146	21.55951	1.152841	3.95E-12	2.35E-10
PTGER3	0.233326	0.105347	-1.1472	7.16E-05	0.000582
CCR5	1.476293	4.344082	1.557072	9.24E-15	1.17E-12
ZNF610	0.40081	0.161553	-1.31091	0.012959	0.0352
LY9	0.15661	0.406001	1.374306	1.93E-08	4.96E-07
RIMKLA	1.062431	0.31203	-1.76761	1.39E-07	2.81E-06
KIAA1257	0.440893	0.187486	-1.23365	1.88E-05	0.000191
PPIL6	0.891102	0.367303	-1.27862	0.000203	0.001361
ABCD2	0.094596	0.194243	1.038012	1.05E-07	2.22E-06
DRC7	0.294894	0.06275	-2.23252	0.04837	0.098502
PPM1H	3.389904	1.459187	-1.21608	2.48E-08	6.20E-07

Gene	conMean	treatMean	logFC	pValue	FDR
FCRLA	0.205215	0.458505	1.159799	9.32E-06	0.000107
SCUBE1	0.054513	0.223951	2.038505	0.000588	0.00313
METTL7A	26.01936	11.13428	-1.22458	1.31E-07	2.69E-06
TRIM22	8.675175	19.44489	1.164426	9.79E-18	2.91E-15
NKG7	8.975489	30.38668	1.759377	2.73E-17	6.90E-15
HCST	5.493461	11.78993	1.101768	3.45E-14	3.60E-12
SLC5A5	0.849886	1.792561	1.07668	0.02414	0.057784
CD96	0.950475	2.620221	1.462969	1.16E-15	1.94E-13
CILP	0.752057	0.341998	-1.13686	0.009682	0.028196
SHISA8	0.682495	0.222452	-1.61733	5.90E-06	7.22E-05
TAP1	45.50997	105.0813	1.207252	7.29E-24	3.32E-20
RPRM	1.339365	0.559333	-1.25977	5.48E-09	1.60E-07
RSPH4A	0.791446	0.203992	-1.95598	0.000777	0.003913
IGFBP5	48.68697	17.7152	-1.45855	2.14E-05	0.000213
MYCN	3.193351	1.38138	-1.20896	6.65E-05	0.000548
PIK3C2G	0.36787	0.179963	-1.03149	0.000104	0.00078
ALDH1A1	62.61407	17.77323	-1.81678	1.75E-07	3.41E-06
GZMA	8.727598	29.90476	1.776718	7.05E-20	4.01E-17
FXYD2	0.095848	0.336911	1.813545	0.001853	0.007772
RARRES3	31.35527	68.38737	1.125022	3.20E-12	1.96E-10
CASC1	0.6833	0.226512	-1.59293	0.000211	0.001398
DLX2	0.558384	0.088435	-2.65857	0.001642	0.007079
CCKBR	0.388533	0.076913	-2.33673	0.009666	0.028187
ITGB7	0.763413	1.612855	1.079081	2.48E-14	2.67E-12
DKK2	0.617395	0.139134	-2.14972	0.035965	0.078933

Gene	conMean	treatMean	logFC	pValue	FDR
KLHDC8A	1.186595	0.338085	-1.81137	2.19E-10	8.93E-09
HCAR3	1.33444	2.894254	1.116957	1.17E-07	2.44E-06
CTSW	3.979725	11.19559	1.49219	1.05E-16	2.32E-14
TSPAN12	5.634998	2.661214	-1.08233	2.72E-05	0.000261
IL1B	3.197625	7.864739	1.298398	1.03E-05	0.000116
CMPK2	3.602383	7.987582	1.148807	9.67E-16	1.71E-13
PTPN7	1.276432	3.055143	1.259123	1.87E-16	3.87E-14
KCNK2	0.576997	0.178758	-1.69056	0.002764	0.010626
AKAP12	3.945182	1.75594	-1.16785	0.000178	0.001213
TDRD1	0.308632	0.009008	-5.09856	0.007183	0.022411
IL27	0.088394	0.207112	1.22839	3.56E-09	1.09E-07
PTPRN	0.761992	0.092623	-3.04033	0.021735	0.053136
NR2F1	5.298801	1.794523	-1.56207	5.25E-08	1.21E-06
CORO1A	8.430665	18.42616	1.128038	1.02E-15	1.78E-13
CCL4	3.233323	9.542158	1.561298	7.12E-16	1.33E-13
NLRP7	1.973649	4.733076	1.261913	2.35E-06	3.32E-05
ROPN1B	0.32831	0.163052	-1.00972	2.90E-05	0.000276
SIGLEC8	0.221405	0.622122	1.490508	4.96E-05	0.000429
LILRB1	0.622061	1.372872	1.142068	6.54E-10	2.36E-08
LMO3	1.004569	0.212807	-2.23896	5.51E-05	0.000467
SOX2	48.63842	20.6392	-1.23671	3.98E-05	0.000358
TTC24	0.077066	0.25508	1.726786	1.63E-14	1.87E-12
TRIML2	0.057177	0.4899	3.098985	0.000628	0.003306
ZMYND10	2.928156	0.834298	-1.81136	0.000133	0.00095
TCEAL6	0.031168	0.013872	-1.16791	0.037912	0.082132

Gene	conMean	treatMean	logFC	pValue	FDR
DLX5	11.1172	2.541395	-2.1291	5.17E-06	6.48E-05
CLDN8	2.572733	1.005053	-1.35603	0.039187	0.084027
SYPL2	0.234512	0.093048	-1.33361	1.40E-05	0.00015
LYG2	0.200623	0.081848	-1.29347	0.012619	0.034515
IL2RG	9.796628	27.07451	1.466578	1.28E-15	2.09E-13
OCA2	1.200257	0.558525	-1.10365	0.044699	0.092883
GPR65	0.45469	0.980792	1.109063	9.03E-13	6.19E-11
APOL6	6.800565	14.4648	1.08882	7.09E-23	1.61E-19
SLAMF6	1.015656	2.732545	1.427834	3.89E-11	1.86E-09
CXCR3	1.312771	4.003353	1.608593	8.52E-15	1.10E-12
FCRL6	0.204113	0.630902	1.628049	9.07E-10	3.18E-08
ST6GAL2	0.603482	0.140287	-2.10493	1.10E-05	0.000121
SEMA5A	1.936061	0.962957	-1.00758	0.000267	0.001685
